# Single cell and genetic analyses reveal conserved populations and signaling mechanisms of gastrointestinal stromal niches

**DOI:** 10.1038/s41467-019-14058-5

**Published:** 2020-01-17

**Authors:** Ji-Eun Kim, Lijiang Fei, Wen-Chi Yin, Sabrina Coquenlorge, Abilasha Rao-Bhatia, Xiaoyun Zhang, Sammy Shun Wai Shi, Ju Hee Lee, Noah A. Hahn, Wasi Rizvi, Kyoung-Han Kim, Hoon-Ki Sung, Chi-chung Hui, Guoji Guo, Tae-Hee Kim

**Affiliations:** 1 Program in Developmental & Stem Cell Biology, The Hospital for Sick Children, Toronto, ON, M5G 0A4 Canada; 2 Department of Molecular Genetics, University of Toronto, Toronto, ON, M5S 1A8 Canada; 3 Center for Stem Cell and Regenerative Medicine, Zhejiang University of School of Medicine, Hangzhou, 310058 China; 4 Translation Medicine Program, The Hospital for Sick Children, Toronto, ON, M5G 0A4 Canada; 5 Department of Laboratory Medicine and Pathobiology, University of Toronto, ON, M5S 1A8 Canada; 6 University of Ottawa Heart Institute, Ottawa, ON, K1Y 4W7 Canada; 7 Department of Cellular and Molecular Medicine, University of Ottawa, Ottawa, ON, K1H 8M5 Canada

**Keywords:** Stem-cell niche, Stem-cell niche

## Abstract

Stomach and intestinal stem cells are located in discrete niches called the isthmus and crypt, respectively. Recent studies have demonstrated a surprisingly conserved role for Wnt signaling in gastrointestinal development. Although intestinal stromal cells secrete Wnt ligands to promote stem cell renewal, the source of stomach Wnt ligands is still unclear. Here, by performing single cell analysis, we identify gastrointestinal stromal cell populations with transcriptome signatures that are conserved between the stomach and intestine. In close proximity to epithelial cells, these perictye-like cells highly express telocyte and pericyte markers as well as Wnt ligands, and they are enriched for Hh signaling. By analyzing mice activated for Hh signaling, we show a conserved mechanism of GLI2 activation of Wnt ligands. Moreover, genetic inhibition of Wnt secretion in perictye-like stromal cells or stromal cells more broadly demonstrates their essential roles in gastrointestinal regeneration and development, respectively, highlighting a redundancy in gastrointestinal stem cell niches.

## Abstract (web summary)

Wnt signals for intestinal stem cell self-renewal originate from the stroma and Paneth cells, but the source in stomach is unclear. Here the authors identify a conserved population of stromal cells adjacent to stomach epithelia where Gli2 activates Wnt ligands to promote gastrointestinal regeneration and development.

## Introduction

Mesenchymal–epithelial cross talk plays a crucial role in organ specification and development^1,2^. Tissue grafting studies showed that tissue-specific mesenchymal signals influence gut endoderm differentiation^3^. Indeed, differential gene expression analysis of the mouse embryonic stomach and intestine identified a stomach mesenchyme-specific factor, *Barx1*, and its deletion caused a striking intestinal transformation, highlighting the significance of organ-specific stromal niche signals^4^. Consistent with these distinct organ niche components, adult stomach, and intestinal epithelial cells are maintained by the activity of stem cells located in discrete epithelial niches, the isthmus and crypt, respectively^5,6^. Wnt signaling plays a critical role in tumorigenesis, adult stem cell homeostasis, and hind gut specification during development^7^. Although thought to be restricted to the intestine and suppressed in the stomach^4,8^, Wnt signaling has recently been shown to perform a surprisingly conserved function in stomach stem cells and their development^9^. However, how and where Wnt ligands in the stomach are produced is unknown^10^.

In contrast to stomach stem cell niches, intestinal stem cell niches have been extensively investigated. Intestinal organoids depend entirely upon Paneth cells, which secrete stem cell niche signals that include Wnt3a^11^. Despite this fact, *Wnt3a* deletion and ablation of Paneth cells in vivo yielded, surprisingly, no obvious stem cell defects, suggesting another sources of stem cell niche signals^12–14^. Genetic inhibition of Wnt secretion from epithelial cells consistently failed to induce stem cell defects^15,16^, while its global inhibition led to the loss of stem cell proliferation^17^. Indeed, additional mouse genetic studies demonstrated that FoxL1+, Pdgfr-α+, and CD34+ mesenchymal cells constitute a critical intestinal stem cell niche that secretes Wnt ligands^18–20^. Most recently, it has been shown that FoxL1 expressing cells represent telocytes, which are in proximity to intestinal epithelial cells, while PDGF-α is more broadly expressed throughout the gut mesenchyme^21^. Interestingly, genetic inhibition of Wnt secretion from αSMA+ myofibroblasts led to no obvious stem cell defects^15^. Despite these significant and complex roles of intestinal stromal cells in stem cell homeostasis, their heterogeneity has not been fully investigated. Of note, Hedgehog (Hh) signaling is known to be active in pericryptal cells, and genetic inhibition of Wnt secretion from these Hh responsive cells arrested colonic stem cells, suggesting its role in the regulation of intestinal stem cell niche signals^17,22^. Interestingly, *Helicobacter pylori* promotes stomach epithelial proliferation by increasing the expression of *R-spondin 3*, a Wnt agonist^23^. Given the surprisingly conserved role of Wnt signaling in both the stomach and intestine, we hypothesized that stomach stromal cells also play a critical role as a stem cell niche.

To determine the role of stomach stromal stem cell niches and define the heterogeneity of both stomach and intestinal stromal cells, we performed single cell transcriptome analysis and identified conserved stromal cell populations that are Hh responsive and that express Wnt ligands and telocyte markers. Our genetic analysis demonstrated the critical role of these cell populations in gastrointestinal regeneration, while revealing their redundancy with other stromal cell populations, such as CD31-positive endothelial and Ly6c-positive stromal cells during development and adult homeostasis. Moreover, our transcriptional analysis shows Hh- and GLI2-mediated transcriptional activation of stem cell niche signals that is conserved in both the stomach and intestine.

## Results

### Conserved gastrointestinal stromal populations

**Fig. 1:**
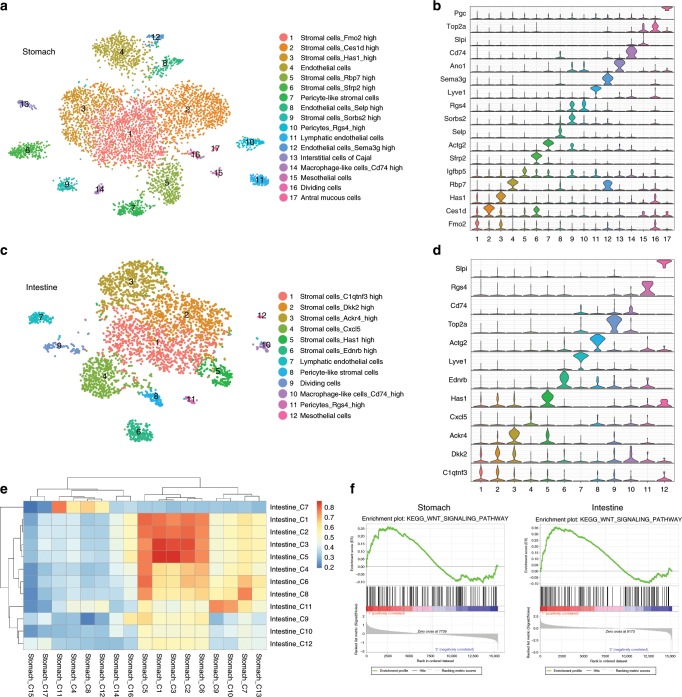
Identification of conserved stromal cell populations between the stomach ad intestine. **a**, **c** t-SNE plots identify 17 different clusters in 4946 stomach cells (**a**) and 12 different clusters in 3459 intestinal cells (**c**). Cells from two mice were pulled for stomach (**a**) and intestinal (**c**) single cell RNA-seq. **b**, **d** Violin plots show expression levels of represented markers within each cluster in the stomach (**b**) and intestine (**d**). **e** Unsupervised hierarchical clustering shows gene expression correlation between the stomach and intestinal stromal cell clusters. A correlation of 0.7 was used to define stromal cell populations conserved between the stomach and intestine. **f**, **g** The gene set enrichment analysis (GSEA) shows enrichment of the WNT signaling pathway in conserved populations compared to distinct populations.

To define the heterogeneity of both stomach and intestinal stromal stem cell niches, we isolated gastrointestinal stromal cells from mice with *Bapx1*^*+/Cre*^ and *Rosa26*^*tdTomato*^ alleles, which broadly label gastrointestinal stromal cells, though not those of the enteric nervous system^24^. We then analyzed their transcriptomes at the single cell level (4946 for stomach and 3459 for intestine each) by performing Drop-Seq (Supplementary Fig. 1)^25^. T-distributed stochastic neighbor embedding (t-SNE) analysis identified more than 10 different stromal cell clusters each for the stomach and intestine (Fig. 1a–d; Supplementary Figs. 1–4, Supplementary Data 1). Furthermore, our unsupervised hierarchical analysis identified stromal cell clusters expressing either conserved or distinct transcriptome signatures between the stomach and intestine (Fig. 1e; Supplementary Figs. 5 and 6). The populations of stromal fibroblast cells (St C1, 2, 3, 6, 9 and Int C1–6), pericyte-like stromal cells (telocyte; St C5, 7 and Int C8), lymphatic endothelial cells (St C11 and Int C7) and pericytes (St C10 and Int C11) expressed similar gene signatures; conversely, mesothelial cells (St C15 and Int C12) and macrophage-like cells (St C14 and Int C10) showed distinct gene expression patterns (Fig. 1a–e; Supplementary Data 1).

**Fig. 2:**
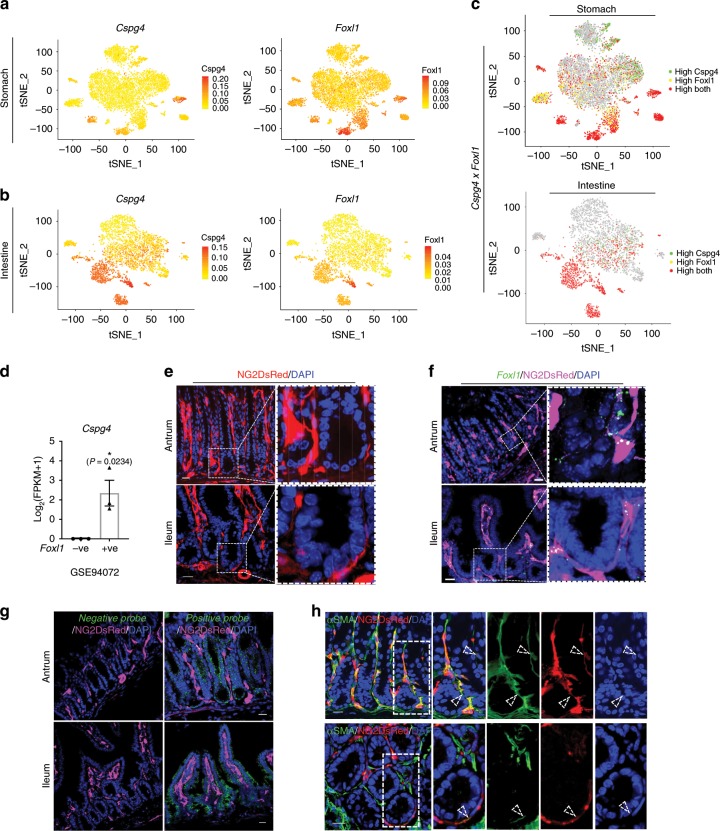
Co-expression of pericyte and telocyte markers by conserved stromal cells. **a**, **b** Feature plot (FP) of *Cspg4* (*Ng2*) and *Foxl1* in stomach (**a**) and intestine (**b**). **c** co-FP of *Cspg4* and *Foxl1* in stomach and intestine t-SNE plot. Red dots mean cells expressing both genes. **d** Comparison of *Cspg4* expression levels between *Foxl1*-positive and -negative cells from the GSE94072 dataset shows highly enriched expression of *Cspg4* in *Foxl1*-positive cells. *n* = 3, biologically independent samples. *P < 0.05 by unpaired student *t* test. Values are mean ± SEM. **e** Real-time Ng2 expression in antral glands and intestinal crypts of *Ng2*^*+/DsRed*^ mice shows pericyte-like stromal cells in proximity to stomach and intestinal epithelial cells. Scale bars indicate 20 μm. **f** Single-molecule FISH (smFISH) of *Foxl1*(green puncta) in *Ng2*^*+/DsRed*^ (magenta) mice. Scale bars indicate 20 μm. Area in dotted squares in left are magnified to right dotted square. **g** Images of negative (left) control with a probe targeting the *DapB* gene (accession # EF191515) from the *Bacillus subtilis* strain SMY, a soil bacterium, and of positive (right) control with probes targeting *Polr2a* (green) and *Ppib* (blue) in the antrum (upper panel) and ileum (lower panel) related to Fig. 2f. Scale bars indicate 20 μm. **h** IF staining of αSMA in *Ng2*^*+/DsRed*^ mice. Empty arrowhead indicates co-expressing cells; filled arrowhead indicates only DsRed positive cells; arrow indicates green color single positive cells. Sub-population of αSMA expressing cells are co-labeled with DsRed. Scale bars indicate 20 μm. Blue is DAPI.

To define the stromal cells expressing stem cell niche signals, we analyzed Wnt ligand expression by performing Markov affinity-based graph imputation of cells (MAGIC)^26^. Many different stromal cell populations expressed Wnt ligand and agonist (i.e., *Rspo*) genes, indicating potentially redundant sources of Wnt niche signals (Supplementary Figs. 7–9). We also found the expression of Wnt antagonist (i.e., *Dkk*) genes in gut stromal cell populations (Supplementary Fig. 10). Of note, the stromal cell populations conserved between the stomach and intestine were more highly enriched for the Wnt pathway, compared to the distinct cell populations (Fig. 1f, g), while some unconserved stromal populations also expressed Wnt ligands (Supplementary Fig. 11). In addition, these conserved cell populations showed robust expression of pericyte markers such as *Ng2* (*Cspg4*) and *Pdgfr-β* (Fig. 2a, b; Supplementary Fig. 12)^27^. Intestinal pericryptal cells located near the stem cells have been implicated in stromal stem cell niche function^17–20^. Our single cell analysis showed that known markers, such as *CD34*, *Pdpn*, and *Pdgfr-*α, of these intestinal pericryptal cells are broadly expressed in gastrointestinal stromal cells, which include the conserved populations (Supplementary Figs. 12 and 13). To validate *Ng2* expression in vivo, we utilized *Ng2*^*+/DsRed*^ and *Ng2-Cre*;*Rosa26*^*+/tdTomato*^ mice, and performed co-labeling immunofluorescence stainings. We found that pericyte markers (Desmin, PDGFRβ, and Nestin) and a villus core stromal cell marker, PDGFRα, but not glial cell markers (s100β and GFAP) are expressed in *Ng2*+ conserved stromal cell populations (Supplementary Figs. 14 and 15). While these cells still express some pericyte markers, they failed to colocalize with CD31+ endothelial cells (Supplementary Fig. 15) and expressed genes distinct from those of gastrointestinal pericytes (Supplementary Fig. 16). Therefore, we will refer to them as pericyte-like stromal cells.

Interestingly, a recent paper has shown that FoxL1 is expressed in telocytes, which constitute a key intestinal stem cell niche^21^, but their role in the stomach as a stem cell niche has been unexplored. Our analysis demonstrated a highly overlapping expression pattern of *Foxl1* and *Ng2* (*Cspg4*) in cell clusters conserved between the stomach and intestine (Fig. 2a–c). Corroborating this data, our analysis of the available data set from a recently published *Foxl1*+ telocyte paper confirmed exclusive expression of *Ng2* in *Foxl1*+ cells (Fig. 2d). Indeed, our analysis of *Ng2*^*+/DsRed*^ mice showed that *Ng2* is highly expressed in pericryptal cells in proximity to gastrointestinal epithelial cells (Fig. 2e). Moreover, single-molecule RNA fluorescence in situ hybridization (smFISH) analysis confirmed the overlapping expression of *Ng2* and *Foxl1* in vivo, indicating that our identified conserved pericyte-like cell populations also include telocytes (Fig. 2f, g). These cells also expressed *Actg2* and its paralog, αSMA (Figs. 1b, d, 2h).

### Wnt secretion by pericyte-like cells during regeneration

**Fig. 3:**
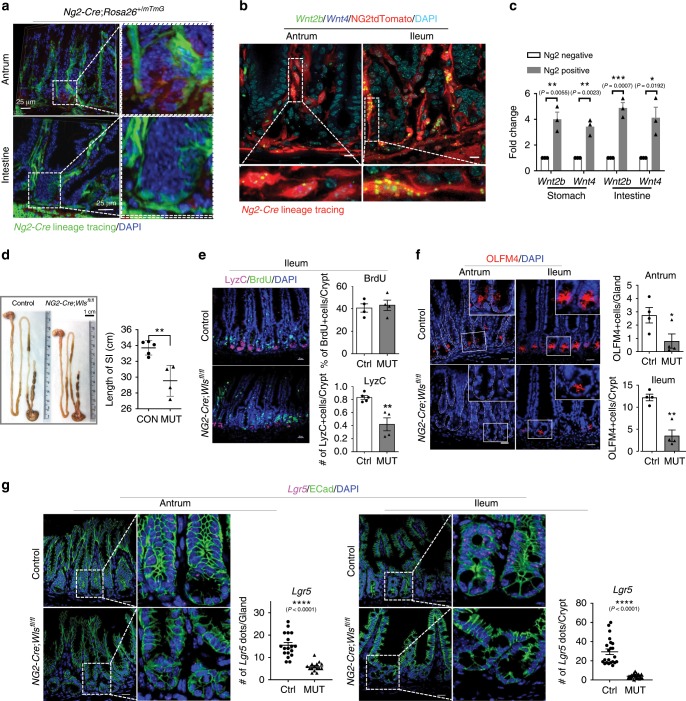
Wnt secretion from pericyte-like stromal cells is required for adult stem cells homeostasis. **a** 3D images of pericyte-like stromal cells surrounding gastrointestinal epithelial cells are visualized by lineage tracing in *Ng2-Cre*;*Rosa26*^*+/mTmG*^ mice. **b** smFISH in *Ng2-Cre*;*Rosa26*^*+/tdTomato*^ mice shows the expression of *Wnt2b* (green puncta) and *Wnt4* (blue puncta) in gastrointestinal pericyte-like stromal cells (tdTomato-red). **c** Quantitative RT-PCRs shows significantly increased levels of *Wnt2b* and *Wnt4* expression in Ng2-tdTomato positive stomach and intestinal stromal cells compared to Ng2-tdTomato negative cells (*n* = 3 per group). **d** The intestinal length is significantly reduced in *Ng2-Cre*;*Wls*^*fl/fl*^ mice compared to the controls (*n* = 5 per group). **e** Immunofluorescence staining (IF) of BrdU and Lysozyme C (a Paneth cell marker) shows no defects in intestinal proliferation but a mildly decreased number of Paneth cells in *Ng2-Cre*;*Wls*^*fl/fl*^ mice compared to the controls (*n* = 4 per group). **f** IF of OLFM4 in antrum and ileum shows significantly decreased numbers of OLFM4+ gastrointestinal stem cells in in *Ng2-Cre*;*Wls*^*fl/fl*^ mice compared to the controls (*n* = 4 per group). Scale bars indicate 20 μm. **g** smFISH of *Lgr5* in the antrum and ileum. Magenta dots indicate *Lgr5* transcripts. The number of *Lgr5* transcripts was significantly reduced in *Ng2-Cre*;*Wls*^*fl/fl*^ mice compared to the controls. Scale bars indicate 20 μm. *P < 0.05, **P < 0.01, ***P < 0.001, ****P < 0.0001. *P* values were determined using nonparametric unpaired Student’s *t* test. Values are mean ± SEM. Each *n* means biologically independent animals and experiments.

To investigate a role for pericyte-like cell populations as a gastrointestinal stem cell niche, we analyzed *Ng2-Cre*;*Rosa26*^*+/mTmG*^ mice and found GFP-positive stromal cells marked by *Ng2-Cre* in close proximity to gastrointestinal epithelial cells surrounding the gland and crypt. In addition to the expression of *Foxl1*^21^ (Fig. 2), immunogold transmission electron microscopy analysis demonstrated that these cells exhibit a long and very thin structure called a telopode (Fig. 3a; Supplementary Figs. 17 and 18). To examine the role of these pericyte-like cells as a source of Wnt niche signals, we performed smFISH in *Ng2-Cre*;*Rosa26*^*+/tdTomato*^ mice and quantitative RT-PCR, and confirmed the expression of Wnt ligands such as *Wnt2b* and *Wnt4* (Fig. 3b, c; Supplementary Figs. 19–21). To determine the significance of these Wnt secreting stromal cells as a stem cell niche, we conditionally deleted *Wntless* (*Wls*), a transmembrane protein essential for the secretion of lipid modified Wnts^28^. The isthmus of antral gastric glands, where Lgr5 is expressed has been also proposed as a potential Wnt niche^10,23,29,30^. Wnt signaling is known to promote intestinal secretory lineage differentiation^31–33^. Although *Ng2-Cre*;*Wls*^*fl/fl*^ mice exhibited a shortened gut length with reduced numbers of goblet cells and Paneth cells, suggesting a reduced level of Wnt signaling, we observed no significant change in gastrointestinal epithelial proliferation (Fig. 3d, e; Supplementary Fig. 22). However, the numbers of both stomach and intestinal stem cells marked by OLFM4 and *Lgr5* were significantly decreased (Fig. 3f, g).

**Fig. 4:**
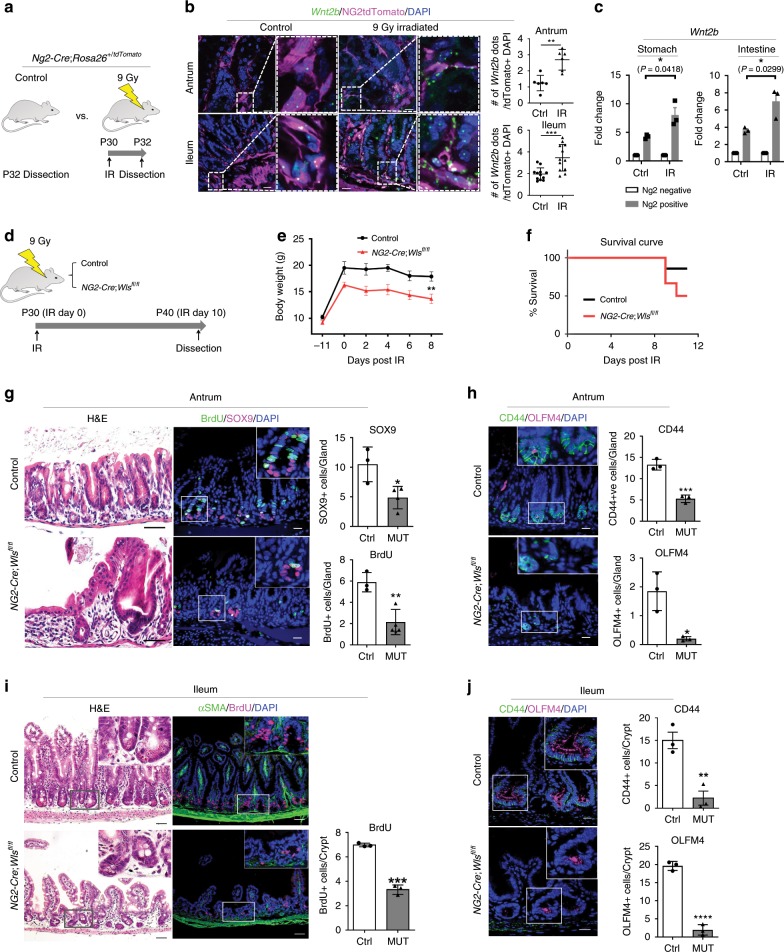
Ng2 + pericyte-like stromal cells constitute a critical stem cell niche during regeneration. **a** Schematic diagram for whole body irradiation and dissection at postnatal day 32 (P32). **b** smFISH of *Wnt2b* (Green) in *Ng2-Cre*;*Rosa26*^*+/tdTomato*^ mice 48 h after whole body irradiation (9 Gy) shows their significantly increased expression compared to the non-irradiation controls (*n* = 3 per group, 2–4 images per mice were counted for *Wnt2b* expression). **c** Quantitative RT-PCR shows increased levels of *Wnt2b* and *Wnt4* expression in Ng2-Tdtomato positive cells after 9 Gy irradiation, compared to non-irradiated Ng2-tdTomato positive cells (*n* = 3 per group). **d** Timeline of whole body irradiation. **e** Body weight change of mice after irradiation (*n* = 6 per group). **f** Survival curve during 10 days after 9 Gy of irradiation (*n* = 7 per group). **g** H&E staining (left), and IF of BrdU (green) and SOX9 (magenta) show a significantly decreased level of gastric epithelial proliferation (BrdU+ cells/gland) and decreased number of Sox9 progenitor cells (Sox9+ cells/gland) in antrum of *Ng2-Cre*;*Wls*^*fl/fl*^ mice compared to the controls (*n* = 3 per group). **h** IF of CD44 (green) and OLFM4 (magenta) show a significantly decreased number of CD44+ and OLFM4+ progenitors and stem cells, respectively, in the antrum of *Ng2-Cre*;*Wls*^*fl/fl*^ mice compared to the controls (*n* = 3 per group). **i** H&E staining (left), and IF of BrdU (magenta) and αSMA (green) show a significantly decreased number of BrdU+ cells in the ileum of *Ng2-Cre*;*Wls*^*fl/fl*^ mice compared to the controls (*n* = 3 per group). **j** IF of CD44 (green) and OLFM4 (magenta) show a significantly decreased number of CD44+ and OLFM4+ progenitors and stem cells, respectively, in the ileum of *Ng2-Cre*;*Wls*^*fl/fl*^ mice compared to the controls (*n* = 3–4 per group). For all, *P < 0.05, **P < 0.01, ***P < 0.001, ***P < 0.001, ****P < 0.0001. *P* values were determined using nonparametric unpaired Student’s *t* test. Scale bars indicate 20 μm. Values are mean ± SEM. Each *n* means biologically independent animals and experiments.

Despite the reduced numbers of stomach and intestinal stem cells in *Ng2-Cre*;*Wls*^*fl/fl*^ mice, no proliferation defects were found (Fig. 3e; Supplementary Fig. 22), and they were able to breed properly, suggesting additional sources of niche signals. Supporting this notion, other stromal cell populations not labeled by *Ng2-Cre* expressed Wnt ligands (Supplementary Fig. 11). Given the proximity of the pericyte-like stromal cells to gastrointestinal epithelial stem cells, their expression of Wnt ligands and the role of Wnt signaling in stem cell renewal^7^, we hypothesized that Wnt secretion from these cells is critical during regeneration. To address this question, we irradiated *Ng2-Cre*;*Rosa26*^*+/tdTomato*^ mice and examined the expression of Wnt ligands. Interestingly, we found significantly increased levels of *Wnt2b* expression in both the *Ng2*-labeled pericyte-like stromal cells (tdTomato+) cells and epithelial cells, suggesting a potential role for pericyte-like stromal cells as a critical niche during regeneration (Fig. 4a–c). To address these stomach and intestinal stroma-specific roles in regeneration, we subjected both control and *Ng2-Cre*;*Wls*^*fl/fl*^ mice to irradiation and analyzed them for regeneration 10 days later (Fig. 4d). When compared to the controls, the survival and proliferation of both stomach and intestinal regenerative progenitors and stem cells were compromised in *Ng2-Cre*;*Wls*^*fl/fl*^ mice, demonstrating the critical role of the pericyte-like stromal cells as a stem cell niche during regeneration (Fig. 4e–j). While *Ng2-Cre* lineage labeled pericryptal cells rarely proliferate in normal adult homeostasis, their proliferation was significantly increased after irradiation (Supplementary Fig. 23a). Consistent with this data, we also found that *Ng2-Cre* lineage labeled pericryptal cells are radioresistant, compared to epithelial cells (Supplementary Fig. 23b). Interestingly, it has been reported that the expression of *Shh* increases after irradiation and promotes *Shh*-mediated tumorigenesis in brain^34^. Indeed, we found an increased level of its expression in epithelial cells (Supplementary Fig. 24). Although this result would likely be influenced by defective stem homeostasis found in *Ng2-Cre*;*Wls*^*fl/fl*^ mice, it still demonstrates the requirement for pericyte-like stromal cells as a Wnt stem cell niche in regeneration.

### Hh-Wnt signaling axis in pericyte-like stromal cells

**Fig. 5:**
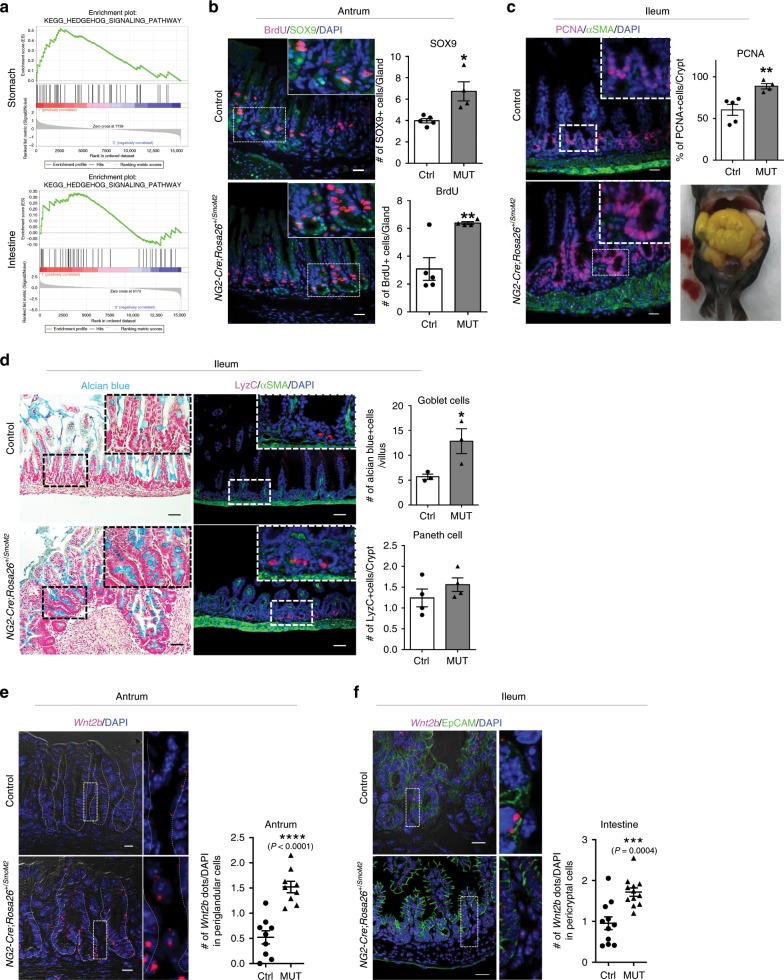
Hh signaling in pericyte-like stromal cells activates epithelial Wnt signaling. **a** GSEA plot demonstrates enrichment of the Hh signaling pathway in conserved pericyte-like gastrointestinal stromal cells compared to distinct populations. **b** IF of BrdU and SOX9 in the stomach antrum. The number of SOX9+ and BrdU+ stomach progenitors are significantly increased in *Ng2-Cre*;*Rosa26*^*+/SmoM2*^ mice compared to the controls (*n* = 3 per group). **c** IF of PCNA and αSMA in the intestine. The number of PCNA+ progenitors is significantly increased in *Ng2-Cre*;*Rosa26*^*+/SmoM2*^ mice compared to the controls (*n* = 3 per group), *P < 0.05. Mutant pups show a dilated small intestine (Right bottom panel). **d** IF of Lysozyme C (LyzC, right panel) and Alcian blue staining (left panel) shows increased numbers of intestinal Paneth cells and goblet cells, respectively, in *Ng2-Cre*;*Rosa26*^*+/SmoM2*^ mice compared to the controls. *P < 0.05. Scale bars indicate 50 μm. **e**, **f** smFISH combined with differential interference contrast (DIC) imaging demonstrates significantly increased levels of *Wnt2b* expression (magenta puncta) in gastrointestinal pericyte-like stromal cells of *Ng2-Cre*;*Rosa26*^*+/SmoM2*^ mice compared to the controls (*n* = 3 per group). Scale bars indicate 20 μm, *P < 0.05, **P < 0.01, ***P < 0.001, ****P < 0.0001. *P* values were determined using nonparametric unpaired Student’s *t* test. Values are mean ± SEM. Each *n* means biologically independent animals and experiments.

We next investigated how these gastrointestinal stromal Wnt niche signals are regulated. It has recently been shown that intestinal pericryptal cells co-express Hh target and Wnt ligand genes, and that Hh-responsive cells constitute a key colonic stem cell niche^17,22^. Activated exclusively in the gastrointestinal mesenchyme^35^, Hh signaling is required for gut epithelial and stromal proliferation, as well as muscle differentiation^36,37^. Mice depleted of Hh signaling also show defects in intestinal stem cells^36^. Therefore, we analyzed Hh target genes in our single cell RNA-seq data and found that the stromal cell populations conserved between the stomach and intestine are highly enriched for the Hh pathway, compared to the distinct stromal cell populations (Fig. 5a). To further investigate the Hh regulation of Wnt signaling, we activated Hh signaling in the *Ng2-Cre* labeled pericyte-like stromal cells and detected elevated levels of both stomach and intestinal epithelial cell proliferation (Fig. 5b, c). Corroborating this data, the numbers of gastric progenitors and stem cells positive for OLFM4^38^, SOX9^39^, and CD44^40^ (Wnt target genes), were significantly increased (Fig. 5b; Supplementary Fig. 25). Consistent with the role of Wnt signaling in intestinal secretory lineage differentiation^31–33^, the number of goblet cells also was significantly increased upon Hh activation during development (Fig. 5d). Moreover, the smFISH analysis showed increased levels of gastrointestinal stromal Wnt ligand expression in these mice, demonstrating Hh activation of stromal stem cell niche signals (Fig. 5e, f; Supplementary Fig. 26).

### GLI2 activation of Wnt ligands in conserved stromal cells

**Fig. 6:**
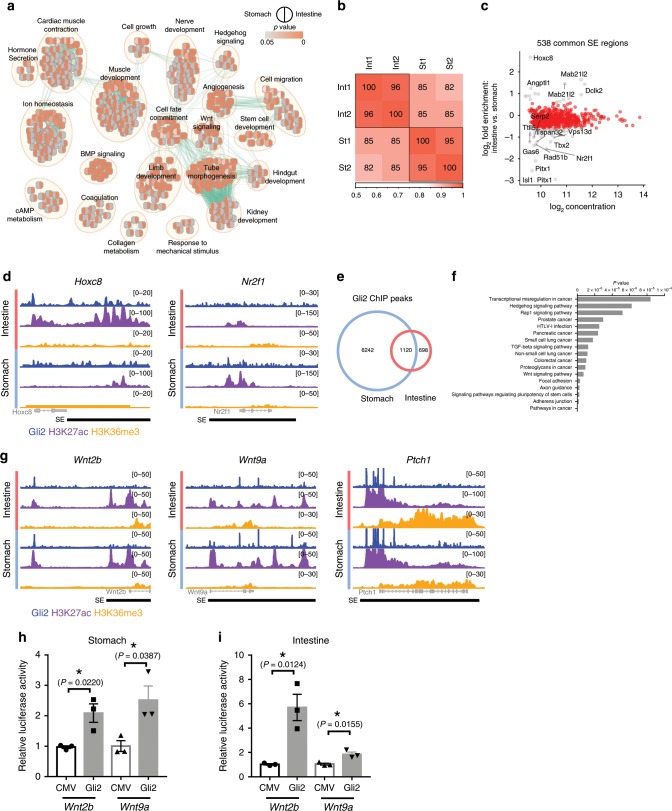
Conserved GLI2 activation of Wnt niche signals between the stomach and intestine. **a** Enrichment network. Functional profiling of genes upregulated > twofold in DKO stomach and intestine vs. control. Left half circle: significance of gene set enrichment in stomach. Right half circle: significance of gene set enrichment in intestine. Analysis revealed that the majority of pathways are commonly enriched in both mutant stomach and intestine. Key genes sets: Hh signaling, Wnt signaling, BMP signaling, stem cell development, muscle development, cell fate commitment, angiogenesis, hindgut development (intestine only), response to mechanical stimulus (stomach only). **b** Correlation heatmap of H3K27ac ChIP-Seq signal at all SE regions identified from DKO stomach and intestine. Plot shows high within-group correlation, and relatively high inter-group correlation, indicating overlapping SEs in the two tissue types. **c** MA plot of H3K27ac ChIP-Seq signal at SE regions in DKO stomach and intestine confirmed findings in the correlation heatmap. Totally, 538 SE regions were common (fold-enrichment between the two tissues < 1.5). Sixteen regions, mapped to 14 unique genes were tissue-specific (fold enrichment > 2). **d** Signals from GLI2, H2K27ac, and H3K36me3 ChIP-Seq experiments show examples of intestine (*Hoxc8*)- and stomach (*Nr2f1*)-specific regions regulated by GLI2. **e** Gli2 ChIP-Seq in DKO stomach and intestine identified over 1100 overlapping regions. **f** KEGG pathway analysis performed on genes associated with the 1120 overlapping Gli2 peaks. Significant enrichment was identified in Hh, TGF-beta, and Wnt signaling pathways. **g** Signals from GLI2, H2K27ac, and H3K36me3 ChIP-Seq experiments show examples of Hh signaling (*Ptch1*) and Wnt ligand (*Wnt2b*, *Wnt9a*) genes regulated by GLI2. All genes are within SE regions, and are bound by GLI2. **h**, **i** Validation of GLI2 activation of luciferase reporter fragments generated from GLI2 peak regions near (**h**) *Wnt2b* and (**i**) *Wnt9a* loci. *n* = 3, Values are mean ± SEM. *P < 0.05. *P* values were determined using nonparametric unpaired Student’s *t* test. Each n means biologically independent animals and experiments.

Although Hh signaling plays a critical role in many different organs and tissues, including the stomach and intestine, the instability of Hh downstream GLI transcriptional factors (TFs) has prevented the mapping of their genomic binding regions at a high resolution^41^. Consequently, understanding its transcriptional mechanisms has proven to be extremely challenging. To overcome this barrier, we utilized mice with conditional alleles of the key Hh-negative regulators, *Sufu* and *Spop*, which are known to act on GLI TFs, sequestering them in the cytoplasm and targeting them to proteasome degradation, respectively^41^. These mice were then crossed with mice carrying a *Bapx1*^*+/Cre*^ allele to specifically delete these genes in the gastrointestinal mesenchyme^24^ (Supplementary Fig. 27). *Bapx1*^*+/Cre*^;*Sufu*^*fl/fl*^;*Spop*^*fl/fl*^ mice stabilized GLI TFs, thereby activating Hh signaling; these mice exhibited severe gastrointestinal defects with abnormal proliferation in both epithelial and mesenchymal tissues (Supplementary Fig. 28). Taking advantage of the stabilized key Hh transcription factor involved in gut development, GLI2^36^, we mapped its genomic binding regions in the intestine, demonstrating direct transcriptional activation of intestinal stromal Wnt ligands by GLI2^42^. Since signaling and transcriptional mechanisms of stomach stromal Wnt niche signals remained unknown, we used these *Bapx1*^*+/Cre*^;*Sufu*^*fl/fl*^;*Spop*^*fl/fl*^ mice to analyze the stomach and compared with the intestinal data. Gene network analysis of the transcriptomes generated from these *Sufu* and *Spop* double knockout (DKO) gastrointestinal tissues showed common enrichment in muscle development and the Wnt pathway (Fig. 6a).

To determine if these Hh target genes are activated via super enhancers, we performed ChIP-Seq for H3K27ac marks with the *Sufu* and *Spop* DKO gastrointestinal tissues and screened for super enhancers by using the Rank Ordering of Super-Enhancers (ROSE) program^43,44^. We found a surprisingly high number (538) of Hh activated super enhancers conserved between the stomach and intestine (Fig. 6b, c). Organ-specific enhancers were also revealed: Stomach- and intestine-specific super enhancers were identified near the organ-specific *Hoxc8*^45^ and *Nr2f1*^46^ loci, respectively (Fig. 6d)^47^. Moreover, our mapping of genome-wide binding sites of GLI2 identified more than one thousand GLI2 peaks conserved between the stomach and intestine (Fig. 6e). The KEGG pathway analysis of these overlapping peaks demonstrated significant enrichment for the Hh and Wnt pathways (Fig. 6f). These peaks were identified in H3K27ac marked enhancer regions conserved between the stomach and intestine, near the Wnt and Hh pathway genes (*Wnt2b*, *Wnt9a*, and *Ptc1*) (Fig. 6g). To validate direct transcriptional regulation by GLI2 in gastrointestinal mesenchymal cells, we were able to culture primary stomach and intestinal mesenchymal cells. We then cloned GLI2-bound regions near *Wnt2b* and *Wnt9a* loci into plasmids containing a luciferase reporter gene and performed a transcriptional reporter assay upon co-expression of *Gli2*. We confirmed GLI2 activation of these reporter genes in gastrointestinal mesenchymal cells (Fig. 6h, i; Supplementary Fig. 29). Corroborating this data, the expression of *Gli2* was enriched in *Ng2-Cre* labeled and *Cspg4* (*Ng2*) expressing pericyte-like stromal cells (Supplementary Fig. 20, 30). Together, this work reveals that the GLI2-mediated transcriptional activation of stromal Wnt niche signals, and this direct regulation in gut stromal cells is conserved between the stomach and intestine.

### Redundant role of stromal Wnt secretion in gut development

Although this study has revealed conserved signaling mechanisms of gastrointestinal stromal Wnt ligands, genetic inhibition of Wnt secretion in *Ng2*-expressing cells and their descendants resulted in only mild defects (Fig. 3d–g; Supplementary Fig. 22). Therefore, the significance of gastrointestinal stromal Wnt niche signals during development is still unclear. In fact, we found that Wnt ligands and/or agonizts are expressed in different gut stromal cell populations (Supplementary Fig. 11). To further define the difference between *Bapx1*^*+/Cre*^ and *Ng2-Cre* lineage labeled stromal cells, we first analyzed the percentage of tdTomato positive cells sorted from *Bapx*^*+/Cre*^;*Rosa26*^*+/tdTomato*^ and *Ng2-Cre*;*Rosa26*^*+/tdTomato*^ mice and found that *Bapx*^*+/Cre*^ labels significantly more stromal populations than *Ng2-Cre* (Supplemental Fig. 31). We then performed mass cytometry (CyTOF) of stomach and intestinal stromal cells isolated from *Ng2-Cre*;*Rosa26*^*+/tdTomato*^ and *Bapx1*^*+/Cre*^;*Rosa26*^*+/tdTomato*^ mice for gut stromal cell type-specific markers. This analysis revealed that compared to *Bapx1*^*+/Cre*^, *Ng2-Cre* labels significantly fewer CD31-positive endothelial cells and Ly6c-positive stromal cells (Supplementary Figs. 32–34). Interestingly, these Ly6c positive cells failed to express a hematopoietic cell marker, CD45, representing unique stromal cell populations expressing Ly6c (Supplementary Figs. 35 and 36). Corroborating this data, our analysis of *Bapx1*^*+/Cre*^;*Rosa26*^*+/tdTomato*^ mice showed that LYVE1-positive lymphatic endothelial cells and PDPN-positive mesothelial cells expressed Wnt ligands and agonizts (Supplementary Figs. 11, 37, and 38). Taken together, these observations suggest a potentially redundant role for gastrointestinal stromal cells as a Wnt stem cell niche. Interestingly, our comparative analysis of single cell transcriptomes with available human gastrointestinal disease genes, identified by genome-wide association studies (GWAS), showed association of intestinal lymphatic endothelial and mesothelial cells with colon cancer and inflammatory bowel disease, also implying their potential roles in disease pathogenesis (Supplementary Fig. 39)^48^.

**Fig. 7:**
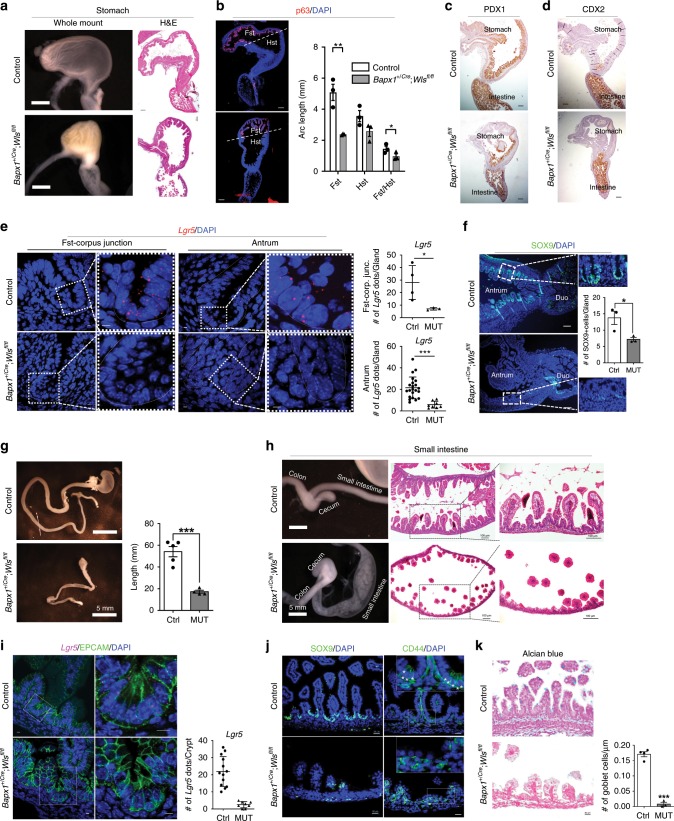
Stromal Wnt secretion is essential for gastrointestinal development. **a** Whole mount and H&E stained images of control and *Bapx1*^*+/Cre*^;*Wls*^*fl/fl*^ stomachs. **b** The arc length of forestomach (Fst) and hindstomach (Hst) measured by imageJ shows a significant reduction of the Fst region in *Bapx1*^*+/Cre*^;*Wls*^*fl/fl*^ mice compared to the controls. The p63 IF staining (red) demarcates Fst and Hst regions. Scale bars indicate 100 μm. **c**, **d** IHC of PDX1 (**c**) and CDX2 (**d**) show a properly maintained stomach-intestinal boundary. Scale bars indicate 100 μm. **e** smFISH of *Lgr5* in Fst-Corpus junction and Antrum. Red dots indicate *Lgr5* transcripts. Number of *Lgr5* transcripts were significantly reduced in *Bapx1*^*+/Cre*^;*Wls*^*fl/fl*^ mice compared to the controls. Scale bars indicate 5 μm. (*n* = 3, 1–4 images per mouse were counted for *Lgr5* expression) (**f**) IF of SOX9 shows a significantly reduced number of SOX9 expressing cells in the antrum of *Bapx1*^*+/Cre*^;*Wls*^*fl/fl*^ mice, compared to the controls. *n* = 3. *P < 0.05. Scales indicate 200 μm. **g**, **h** Whole mount (**g**) and H&E stained images (**h**) of control and *Bapx1*^*+/Cre*^;*Wls*^*fl/fl*^ intestines. The intestinal length was dramatically decreased in *Bapx1*^*+/Cre*^;*Wls*^*fl/fl*^ mice compared to the controls. Scale bars indicate 100 μm. *n* = 4. **i** smFISH of *Lgr5* (magenta dots) with EPCAM IF staining (an epithelial marker; green) in the intestines. The number of *Lgr5*+ magenta dots (transcripts) were significantly reduced in *Bapx1*^*+/Cre*^;*Wls*^*fl/fl*^ mice compared to controls. Scale bars indicate 5 μm. (*n* = 3, 4–5 images per mice were counted for *Lgr5* expression) (**j**) IF of SOX9 (left-green) and CD44 (right-green) show their severely impaired expression in *Bapx1*^*+/Cre*^;*Wls*^*fl/fl*^ intestines. * marks indicate CD44+ cells. **k** Alcian blue staining shows a significantly decreased number of goblet cells in *Bapx1*^*Cre/+*^;*Wls*^*fl/fl*^ intestines compared to the controls, *n* = 3, ****P < 0.0001. For all figures, all embryos were analyzed at E17.5. Values are mean ± SEM. *P < 0.05, **P < 0.01, ***P < 0.001. *P* values were determined using nonparametric unpaired Student’s *t* test. Each n means biologically independent animals and experiments.

To address this potentially redundant role for Wnt-secreting gastrointestinal stromal cells, we used *Bapx1*^*+/Cre*^ mice to more broadly target gastrointestinal stromal cells (Supplementary Figs. 1a and 31). *Bapx1*^*+/Cre*^;*Wls*^*fl/fl*^ mice exhibited remarkably severe phenotypes throughout the gastrointestinal tract (Fig. 7a–k; Supplementary Fig. 40). Consistent with the observation of Wnt signaling activation in the mouse forestomach, epithelial tissue during development^4^, *Bapx1*^*+/Cre*^;*Wls*^*fl/fl*^ mice showed a significant decrease in the size of the forestomach accompanied by reduced levels of epithelial proliferation, while properly maintaining a stomach–intestine boundary (Fig. 7a–d). In addition, the intestinal lumen was severely distended, and the intestine length was dramatically reduced; hematoxylin-eosin (H&E) staining also revealed epithelial depletion, with significantly reduced numbers of villi (Fig. 7g, h). Since this phenotype is morphologically similar to the gastric transformation defects found in *Cdx2* knockout intestines^49^, we also analyzed gastric markers and CDX2 by IHC but found no transformation (Fig. 7b–d; Supplementary Fig. 40a). To analyze stem cell defects, we performed smFISH for *Lgr5* and found its significantly reduced expression in both the stomach and intestine, demonstrating severe defects in developing stem cells (Fig. 7e, i; Supplementary Fig. 41). To measure epithelial Wnt signaling activity, we also analyzed the expression of known Wnt target genes such as SOX9 and CD44, and found a dramatic reduction of these markers (Fig. 7f, j; Supplementary Fig. 40b). Consistently, epithelial proliferation and secretory cell differentiation, both of which depend on Wnt signaling, were severely impaired (Fig. 7k; Supplementary Fig. 40c, d). These data demonstrate the essential role of gastrointestinal stromal Wnt niche signals during development.

## Discussion

Wnt signaling had been thought to be restricted to the hindgut and critical for its specification^6^. Indeed, its abnormal activation in the stomach through the deletion of the stomach-specific mesenchymal factor, *Barx1*, led to partial intestinal transformation^4^. In addition, the intestinal stem cell marker, *Lgr5*, was first identified by analyzing Wnt target genes^50^. Interestingly, *Lgr5* was also expressed in a subpopulation of the antrum^29,30^; Wnt reporter expression was found to be temporarily active during stomach development, becoming later restricted to the distal stomach region, suggesting its temporal and regional roles^4,23^. Corroborating these data, recent studies have revealed a critical role for Wnt signaling in stomach development and stem cells^9^. In the intestine, Wnt ligands are expressed by Paneth cells surrounding stem cells^11^, and a number of studies have shown that mesenchymal cells also constitute a critical source of Wnt ligands^11,15,16,18–20,22^. However, neither the source of Wnt niche signals, nor how they are produced in the stomach was known. The stomach has no Paneth cells that would serve as source for Wnt ligands, implying that mesenchymal cells are a major Wnt stem cell niche. Here, our single cell and mouse genetic analyses demonstrate for the first time that stomach stromal cells constitute a critical Wnt stem cell niche during development and regeneration. We also demonstrate that the Hh downstream transcription factor, GLI2, directly induces the expression of Wnt ligands in the stomach. Since our single cell analysis was performed with stromal cells isolated from the glandular hindstomach, which includes both the corpus and the antrum, separate single-cell analysis would enable further definition of the stomach region-specific stromal niches.

Despite the fact that tissue cross-talk is known to provide tissue-specific niche signals to properly specify distinct organs^1,2^, our work not only identifies stromal cell populations that are highly conserved between the stomach and intestine, but also reveals a conserved mechanism by which Hh signaling induces stromal Wnt niche signals. A recent study has shown that stromal Hh-GLI2 provides key niche signals, which include both Wnt2b and Wnt2, to the mammary epithelial stem cells^51^. However, how these niche signals are produced by GLI2 was unknown. In addition to our earlier work in the intestine^42^, our current work in the stomach shows direct transcriptional activation by GLI2 of Wnt ligands such as *Wnt2b* and *Wnt9a*, further supporting Hh and GLI2-mediated direct activation of Wnt ligands as a conserved mechanism responsible for producing stromal stem cell niche signals. Mesenchymal Hh signaling is known to promote different types of cancer^52^. Our proposed Hh-GLI2 activation of stromal stem cell niche signals may also contribute to tumorignesis in different types of cancer.

Interestingly, genetic inhibition of Wnt secretion in Ng2-derived cells, which include telocytes, led to much milder stem cell defects than the broad range of striking stem cell arrest defects found by Shoshkes-Carmel et al. Our work differs from these studies in that we utilized mice with the *Ng2-Cre* allele, which is expressed throughout embryonic development, in contrast to their inducible inhibition of Wnt secretion at the adult stage using the *Foxl1-CreERT2* allele. During development, these telocytes and other stromal cells might have adapted to produce compensatory stem cell niche signals to prevent drastic stem cell failure. Supporting the redundancy of stromal stem cell niches, we showed that Wnt ligands are expressed in different cell types throughout the mesenchyme (Supplementary Figs. 7 and 11). We also demonstrated that inhibition of Wnt secretion in stromal cells broadly leads to severe stem cell and developmental defects (Fig. 7). Although our *Ng2-Cre* lineage tracing showed ubiquitous labeling throughout the proposed telocyte region in proximity to gastrointestinal epithelial cells (Fig. 2e), and single cell analysis shows a significant overlap between *Ng2* and *Foxl1* expression (Fig. 2a–c), another possibility is that *Foxl1* also labels rare but critical stromal cells not targeted by *Ng2-Cre* lineage tracing. Of note, a recent study has shown striking resistance of intestinal stromal cells to tamoxifen inducible Cre activation^53^. This data suggests that telocytes among intestinal stromal cells might have lost their resistance during evolution. While further investigation would be needed to better understand this cell population, genetic inhibition of Wnt secretion in *Bapx1*^*Cre*^-derived cells have demonstrated the significance of redundancy among different gastrointestinal stromal cell populations secreting stem cell niche signals. Although both *Bapx1*^*+/Cre*^ and *Ng2-Cre* lineage-derived cells labeled telocytes, our single cell RNA-Seq and CyTOF analyses highlighted their difference. For example, many more CD31-positive endothelial cells and Ly6c positive stromal cells were represented in *Bapx1*^*Cre*^-derived cells, compared to *Ng2-Cre* lineage labeled cells (Supplementary Figs. 32–36). Importantly, *Ly6c* is highly expressed in Hh signaling responsive colonic stromal cell populations that constitute a critical stem cell niche, further supporting their potential redundancy as an intestinal stem cell niche^22^. The identity of this stromal population would require further investigations.

Taken together, our study has revealed not only the conserved signaling and transcriptional mechanisms of stomach and intestinal stromal stem cell niches but also unexpected redundancy among stromal cell populations. Interestingly, telocytes have been found in many different mammalian organs^54^. Therefore, it would be useful to determine if Hh and GLI2-mediated activation of Wnt ligands is also conserved in the telocytes of other organs.

## Methods

### Mouse strains

Mice were housed in specific pathogen free barrier facilities. Mice handling and all experiments performed were monitored in accordance with protocols approved by The Centre for Phenogenomics (TCP) Animal Care Committee. *Bapx1*^*Cre*^ mice were obtained as a.pngt from Dr. Warren Zimmer. A *Spop*^*fl*^ allele was obtained from the trans-NIH knock-out mouse project (KOMP) Repository. *Ng2-Cre*, *Ng2*^*DsRed*^, *Myh11-CreERT2*, *Rosa26*^*mTmG*^, *Rosa26*^*tdTomato*^, *Rosa26*^*SmoM2*^, and *Wls*^*fl*^ mice were purchased from The Jackson Laboratory. Embryos were dissected at E17.5 or E18.5; mice 2–5 weeks of age were used for analyses. Cre recombinase was activated by gavage of 1 mg tamoxifen (Invitrogen) or by intraperitoneal injection of 1 mg tamoxifen for 2 or 3 days. Tamoxifen was dissolved in corn-oil (Sigma) as a stock concentration of 10 mg/ml. BrdU (BD Biosciences, 50 mg/kg) was injected intraperitoneally in mice, and they were sacrificed 1 h later.

### Stromal cell isolation for single-cell RNA sequencing

Single-cell sequencing of stomach and intestine was performed with tdTomato+ cells isolated from male *Bapx1*^*+/Cre*^;*Rosa26*^*+/tdTomato*^ mice (5 weeks old). Mesenchymal cells were isolated from the glandular hindstomach, which includes both the corpus and the antrum, and used for stomach scRNA-Seq, while mesenchymal cells from the whole intestine were used for intestinal scRNA-Seq. Mesenchymal cell isolation from the stomach and intestinal tissues was modified from the previously described method^55^. Mice were dissected in ice-cold Wash Buffer; HBSS (Gibco) with 2% (vol/vol) of fetal calf serum (Gibco) and 10 mM HEPES (15630-080, Gibco). Intestines were cut into small pieces and moved to pre-warmed Predigestion Buffer: 30 ml of HBSS with 10% (vol/vol) inactivated FBS (080-105, Wisent Bioproducts), 10 mM HEPES and 5 mM EDTA (15575-038, Invitrogen). This solution was then incubated for 20 min at 37 °C under rotation (220 rpm) in a thermal incubator in a 50 ml tube, followed by filtering through a 100 μm cell strainer. The flow-through contains stomach or intestinal contents decanted off epithelial cells. These gut pieces were incubated for 20 min at 37 °C under slow rotation, followed by filtering through a 70 μm cell strainer. After wash off the remaining EDTA with Wash Buffer, the tissues were collected into 50 ml tubes containing 20 ml of Digestion Buffer; 1% (vol/vol) penicillin–streptomycin antibiotics (P4333, Sigma), 10% inactivated FBS, 15 mM HEPES, 100 U/ml of DNaseІ (LS002139, Worthington), 25 U/ml of collagenase IV (LS004186, Worthington), 0.3 g/100 ml of Dispase (04942078001, Roche). The tissues were digested by incubation at 37 °C for 30 min under slow rotation. After vortexing the cell solution intensely for 20 s every 10 minutes, the tissues were passed through a 40 μm cell strainer. After collecting them into 50 ml tubes with 20 ml fresh Digestion Buffer, the previous incubation process was repeated. After combining the supernatants from digestion steps and centrifugation for 5 min at 400*g* at 20 °C, the pellets were resuspended in Wash Buffer for FACS. CytoxBlue (S34857, Invitrogen) staining was used to distinguish dead cells. tdTomato-positive cells were sorted using MoFlo Astrios (Beckman Coulter).

### Drop sequencing

Drop-Seq was performed as described, using a Dolomite Bio (Royston, UK) single-cell RNA-Seq system^25^. Three mice were used to obtain 500,000 of cells for 4000 stamps of drop-seq. The finally recovered cells numbered 4946 for the stomach and 3459 for the intestine each. The viability and number of sorted cells were checked by performing the trypan blue (15250061, Invitrogen) exclusion test prior to Drop-Seq. These cells were then diluted at a concentration of 14 K cells/ml into PBS+ 0.01% BSA. Barcoded Beads SeqB (ChemGenes Corp., Wilmington, MA, USA) were diluted at a concentration of 200 particles/µL into 200 mM Tris pH 7.5, 6% Ficoll PM-400, 0.2% Sarkosyl, 20 mM EDTA, 50 mM DTT.

### Quality control of single-cell RNA-Seq data

The quality control metrics for the single-cell RNA-Seq data were obtained using the RNA-SeQC tool (v1.1.7)^56^. This program accepts aligned files as input and delivers a series of plots and statistics for each sample. The quality of the raw reads is defined by a probability distribution across each base call.

### Data clustering

DGE expression matrices were processed and analyzed using the R package Seurat. Only genes expressed in at least 3 cells and cells with minimum gene numbers of 100 for the stomach and 150 for the intestine were kept for downstream clustering. We chose approximately 2000 highly variable genes to project the scaled cpm (counts per million) normalized data into low dimensional subspace and perform component analysis. Significant PCs were selected before the curve of the standard deviations reached the plateau. Two-dimensional t-SNE was used for visualization. Clustering was based on density clustering. A likelihood-ratio test was used to find the differentially expressed genes for identify classes^57^. To overcome the dropout effect in single cell data, we used MAGIC as previously described^26^. We ran the MAGIC with the same parameters (*k* = 10, *a* = 15) in both stomach and intestinal data sets. For better visualization, we used the recovered data set to access the expression levels of *Wnt* and other gut stromal marker genes, which were projected into the feature plot in R package Seurat. For the co-expression feature plot, we used the mean value as the cutoff to determine whether the gene is expressed in one cell or not.

### Cell types comparison between the stomach and intestine

We used an average expression level of the top 30 marker genes as the feature for each cluster in both the stomach and intestine. Then we calculated the Spearman correlations to compare cell types. A correlation of 0.7 was used to select matching cell types in the stomach and intestine. For further validation, supervised MetaNeighbor^58^ with highly variable genes from the function”get_variable_genes” was used to calculate the AUROC across each cell type from both stomach and intestine.

### GSEA analysis

We used average expression of each cluster for analysis with GSEA (version 3.0.0)^59,60^. All the default options were used for GSEA.

### Histology, immunohistochemistry, and immunofluorescence

Mouse intestines were dissected in cold 1× PBS and fixed overnight in 4% paraformaldehyde (PFA) at 4 °C. The tissues were processed, embedded in paraffin blocks, and sectioned at 4 μm. For histology, the tissue sections were deparaffinized in xylene, rehydrated through alcohol gradients, and stained with hematoxylin and eosin (Sigma), Alkaline phosphatase substrate (ThermoFisher Scientific), Alcian blue (Sigma) and/or nuclear fast red (Sigma). The cover slips were then mounted using CytoSeal Mounting Medium (Electron Microscopy Science). For immunohistochemistry (IHC)-Paraffin sections, antigens were retrieved in 10 mM sodium citrate buffer (pH6.0). These sections were permeabilized with 0.3% Triton X-100 (Sigma), blocked with 5% fetal donkey serum (Gibco) and Avidin/Biotin Blocking kit (Vector), and incubated overnight with anti-Lysozyme C (Santa Cruz, 1:200) or anti-Pdx1 (Developmental Studies Hybridoma Bank, 1:300). After washing followed by incubation with biotin-conjugated anti-goat, or mouse antibody (Vector Laboratories, 1:300), the staining was developed by using the DAB Substrate Kit (SK-4100, Vector Laboratories). For immunofluorescence (IF)-Paraffin sections, anti-Cdx2 (MU392A-UC, Biogenex, 1:300), anti-BrdU (BDB347580, Fisher scientific, 1:200), anti-CD44 (550538, BD pharmigen, 1:100), anti-Sox9 (AB5535MI, EMD Millipore, 1:200), anti-p63 (619001, BioLegend, 1:300), anti-H,K-ATPase (D032-3H, MBL, 1:400), anti-pHH3 (05-806, Millipore, 1:200) or anti-PCNA (18-0110, Invitrogen, 1:200) was directly incubated overnight after the permeabilization and blocking steps described above. After washing, sections were incubated with Alexa Fluor 594-, 488-conjugated anti-rabbit, rat or mouse IgG (Invitrogen). CD44 immunostaining was enhanced by using the Tyramide Signal Application kit (Perkin-Elmer). For IF-Frozen sections, the tissues embedded in optimal cutting temperature (OCT) compound (Sakura) were sectioned into 10 μm and washed with 1× PBS. After permeabilization and blocking with 0.3% Triton X-100, 5% fetal donkey serum (Gibco) for 1 h, the tissue sections were incubated overnight with anti-αSMA (ab124964, Abcam, 1:2000), anti-Desmin (Ab32362, Abcam, 1:1000), anti-PDGFRβ (Ab32570, Abcam, 1:100), anti-PDGFRα (SC-338, Santa Cruz, 1:200), anti-Nestin (Ab6142, Abcam, 1:200), anti-s100β (ab41548, Abcam, 1:200), anti-GFAP (Z0334, Dako, 1:300), anti-Lyve1 (11-034, Angiobio, 1:400), anti-CD34 (ab81289, abcam, 1:300), anti-CD31 (550274, BD Bioscience, 1:300). E-Cadherin (610183, BD Bioscience, 1:300) or anti-RFP (600-401-379, Rockland Inc, 1:300) antibodies. The cover slips were then mounted using Fluoroshield Mounting Medium with DAPI (ab104139, Abcam). The slides were reviewed using a Nikon E1000 microscope with NIS-Elements BR 4.12.01 imaging software.

### Quantitative reverse-transcription PCR (qRT-PCR)

RNA was extracted from FACS sorted cells using RNeasy Plus micro kit (74034, QIAGEN), followed by cDNA synthesis with the SuperScript III First-Strand synthesis kit (11752050, ThermoFisher Scientific), according to the manufacturer’s instructions. Transcript levels were determined using SYBR Green PCR master mix and the ViiA7 system (4368577, Applied Biosystems). qRT-PCR assays were conducted in triplicates and normalized to the *Actin* transcript level. The comparative CT method was used to analyze the data. The primer list is shown in Supplementary Table 1.

### Small-molecule fluorescence in situ hybridization (smFISH)

Mice were perfused with 2% PFA and fixed overnight in 4% PFA at 4 °C. The tissues were washed with 1× PBS three times for 30 min, moved to 30% sucrose, and incubated overnight. The tissues embedded in OCT were sectioned, and we performed the manufacturer’s protocol for single-molecule FISH that is described in the RNAscope Multiplex Fluorescent Detection Kit v2 (323110, ACDBio). The RNAscope Probe (ACDBio)-Mm-*Wnt2b* (405031-C2), *Wnt4* (401101-C2), *Wnt2* (313601), *Foxl1* (407401), *Rspo3* (402011-C3), *Rspo1* (479591-C2), *Lgr5* (312171-C3), or *Shh* (314361-C2) was used for single-molecule FISH. The images were acquired with a Nikon A1R Confocal microscope.

### Luciferase reporter assay

Stomach and intestinal mesenchymal primary cells were cultured as previously reported^61^ and co-electroporated with *Wnt2b* or *Wnt9a* luciferase, and pRL-TK control plasmids (E2240, Promega), with or without the *Gli2* expression plasmid. Electroporation was performed using NEON (Invitrogen; Voltage 1400 V, pulse width 30 ms and pulse number 1). These cells were incubated in antibiotics-free culture medium (DMEM supplemented with 10% fetal bovine serum, 1% non-essential amino acid and 1% l-Glutamine) 24 h prior to electroporation, which was replaced with fresh culture medium containing antibiotics 24 h after electroporation. They were then harvested 36 hours post-electroporation and lysed to determine luciferase activity, using the Dual Luciferase Reporter Assay System (E0910, Promega) and the Lumat LB 9507 luminometer (ThermoFisher Scientific).

### Quantification and statistical analysis

All results were performed in duplicates or triplicates, and expressed as mean ± S.E.M.; *P* values were determined using nonparametric unpaired Student’s *t* test (Mann–Whitney *U* test). *P* < 0.05 was considered as statistically significant.

### Random forest based similarity learning for scRNA-seq data

We used random forest based similarity learning (RAFSIL) to learn cell–cell similarities of different cell types in each tissue and verify the clustering result from the SNN algorithm. RAFSIL implements a two-step procedure, where feature construction geared towards scRNA-seq data is followed by similarity learning. We chose the top 20 marker genes of each cluster as input features. We modified RAFSIL implementation by pooling data from every 20 cells randomly selected within each cell type and tissue, then applying RAFSIL to the average gene expression profile of the pooled data. We also replaced the randomForest() in RAFSIL with randomForest() in paralleRandomForest. These two simple modifications effectively increase data quality, while reducing the computational burden. paralleRandomFores

### Cell type specific expression of disease-associated genes

Using normalized expression values, we obtained a *z*-score for each cell, then computed the mean *z*-score for each cluster. Genes that display any cluster specific-enrichment (*z* > 1.65) were kept for further analysis. We acquired disease-associated SNPs and their neighboring genes from the NHGRI-EBI catalog (https://www.ebi.ac.uk/gwas/)^48^ and plotted the *z*-transformed expression of genes for each disease term individually. Genes were clustered using hclust function (R package: stats) with default parameters, while cell clusters were plotted in the order described above.

### Reporting summary

Further information on research design is available in the Nature Research Reporting Summary linked to this article.

## Supplementary information

Supplementary Information

Reporting Summary

Description of Additional Supplementary Files

Supplementary Data 1

## Data Availability

The single-cell RNA-seq, bulk RNA-seq and ChIP-seq data set generated for the current study is available in the GEO (Gene Expression Omnibus) database repository: Accession number for single-cell RNA-seq: GSE116514. Accession numbers for RNA-seq data: GSE114450 (stomach), GSE103683 (intestine). Accession numbers for ChIP-seq data: GSE114449 (stomach), GSE103690 (intestine). The source data underlying Figs. 2d, 3c–g, 4b, c, 4g–j, 5b–f, 6h, i, 7b, and 7e–k, and Supplementary Figs. 4a, b, 20, 22c–e, 23a, b, 24a, b, 25b, 28a, b, and 31 are provided as a Source Data file.

## Source data

Source Data
