# Supplementary material for: Single cell and genetic analyses reveal conserved populations and signaling mechanisms of gastrointestinal stromal niches
Source: Nat Commun. 2020 Jan 17;11:334. doi: 10.1038/s41467-019-14058-5 (PMC6969052; doi:10.1038/s41467-019-14058-5)
Supplement: Supplementary file 1 — Supplementary Information [file 41467_2019_14058_MOESM1_ESM.pdf]

Supplementary Information

Single cell and genetic analyses reveal conserved populations and signaling mechanisms of gastrointestinal stromal niches

Ji-Eun Kim[1,2], Lijiang Fei[3], Wen-Chi Yin[1,2], Sabrina Coquenlorge[1,2], Abilasha Rao-Bhatia[1,2], Xiaoyun Zhang[1,2], Sammy Shun Wai Shi[4,5], Ju Hee Lee[4,5], Noah A. Hahn[1,2], Wasi Rizvi[1,2], Kyoung-Han Kim[6,7], Hoon-Ki Sung[4,5], Chi-chung Hui[1,2], Guoji Guo[3], Tae-Hee Kim[1,2,‡]

[1]Program in Developmental & Stem Cell Biology, The Hospital for Sick Children, Toronto, Ontario M5G 0A4, Canada; [2]Department of Molecular Genetics, University of Toronto, Toronto, Ontario M5S 1A8, Canada; [3]Center for Stem Cell and Regenerative Medicine, Zhejiang University of School of Medicine, Hangzhou 310058, China; [4]Translation Medicine Program, The Hospital for Sick Children, Toronto, Ontario M5G 0A4, Canada; [5]Department of Laboratory Medicine and Pathobiology, University of Toronto, Ontario M5S 1A8, Canada; [6]University of Ottawa Heart Institute, Ottawa, Ontario K1Y 4W7, Canada; [7]Department of Cellular and Molecular Medicine, University of Ottawa, Ottawa, Ontario K1H 8M5, Canada

[‡]**Correspondance to:** Tae-Hee Kim at tae-hee.kim@sickkids.ca

This file includes:

Supplementary Methods
Supplementary Figure 1. Overview of single-cell RNA-seq.
Supplementary Figure 2. Random forest based similarity learning (RAFSIL) for single-cell RNA-seq data.
Supplementary Figure 3. Dot plots with representative, differentially expressed markers of single-cell RNA-seq data.
Supplementary Figure 4. Percentage of each cluster in scRNA-seq data.
Supplementary Figure 5. Unsupervised hierarchical clustering including correlation matrices to compare stomach and intestinal clusters.
Supplementary Figure 6. Unsupervised hierarchical clustering including representative cluster markers to compare stomach and intestinal clusters.

Supplementary Figure 7. Expression of Wnt ligand genes in gastrointestinal stromal cells.

Supplementary Figure 8. Expression of R-spondin genes in gastrointestinal stromal cells.

Supplementary Figure 9. Violin plots for Wnt ligands and Rspo family in stomach and intestial stromal cell.

Supplementary Figure 10. Violin plots of Wnt antagonists in the stomach and intestine.

Supplementary Figure 11. Heatmap of stromal cell markers and Wnt ligands in the conserved and unconserved stomach and intestinal clusters.

Supplementary Figure 12. Violin plots of pericyte and intestinal pericryptal cell markers in the stomach and intestine.

Supplementary Figure 13. Expression of pericyte and intestinal pericryptal cell markers in the stomach and intestine.

Supplementary Figure 14. Characterization of real-time *Ng2* expressing cells from $Ng2^{+/DsRed}$ mice in the antrum**.**

Supplementary Figure 15. Characterization of *Ng2-Cre* lineage traced cells.

Supplementary Figure 16. Heatmap of differential stromal cell markers in gastrointestinal pericyte-like (telocyte) and pericyte clusters.

Supplementary Figure 17. Pericyte-like stromal cells surrounding the antral gland and crypt with a mesh-like structure.

Supplementary Figure 18. Immunogold transmission electron microscopy (TEM) analysis of pericyte-like stromal cells surrounding the crypt.

Supplementary Figure 19. Expression of *Wnt2b* in *Ng2-Cre* lineage traced stomach and intestinal stromal cells.

Supplementary Figure 20. Characterization of *Ng2-Cre* lineage traced cells using quantitative reverse transcript PCR (qRT-PCR).

Supplementary Figure 21. smFISH images of negative and positive probes in gastrointestinal tissues.

Supplementary Figure 22. Gastrointestinal differentiation and proliferation upon pericyte-like stromal cell-specific inhibition of Wnt secretion.

Supplementary Figure 23. Proliferation and survival of pericyte-like stromal cells after irradiation.

Supplementary Figure 24. Increased *Shh* transcripts after irradiation in gastrointestinal epithelial cells.

Supplementary Figure 25. Increased numbers of gastrointestinal progenitors upon Hh activation in pericyte-like stromal cells.

Supplementary Figure 26. smFISH images of negative and positive probes in gastrointestinal tissues.

Supplementary Figure 27. Schematic diagram for Gli2 ChIP-seq.

Supplementary Figure 28. Percentage of αSMA expressing cells in the mesenchyme of mutant pups used for GLI2 ChIP-seq.

Supplementary Figure 29. Luciferase assays of genomic regions.

Supplementary Figure 30. co-featured plots (FP) of *Cspg4*, *Gli2*, *Wnt2b* and *Wnt9a* in the stomach and intestine.

Supplementary Figure 31. *Bapx1*$^{+/Cre}$ labels more mesenchymal cell populations than *Ng2-Cre*.

Supplementary Fig. 32. The expression pattern of all markers used for CyTOF analysis.

Supplementary Fig. 33. Major difference of CD31 expressing clusters between *Bapx1*$^{+/Cre}$ and *Ng2-Cre* labeled cells.

Supplementary Fig. 34. Feature plots and violin plots of *Ly6c* and *Pecam* (*Cd31*) analyzed from the published GSE113043 data set and our gastrointestinal scRNA-seq data.

Supplementary Figure 35. Feature plots from gastrointestinal scRNA-seq data for *Ly6c* and *Cd45* (*Ptprc*).

Supplementary Fig. 36. t-SNE plots from gastrointestinal CyTOF data for Ly6c and CD45.

Supplementary Figure 37. Gut stromal cell populations labeled by *Bapx1*$^{+/Cre}$ but not by *NG2-Cre* lineage tracing.

Supplementary Figure 38. Mesothelial cells are labeled by *Bapx1*$^{+/Cre}$ but not by *NG2-Cre* lineage tracing.

Supplementary Figure 39. Heatmap of gastrointestinal stromal cluster expression of genes correlated to GWAS data.

Supplementary Figure 40. Normal gastrointestinal specification and stem cell defects in *Bapx1*$^{+/Cre}$;*Wls*$^{fl/fl}$ mice.

Supplementary Figure 41. smFISH images of negative and positive probes in gastrointestinal tissues.

Other supplementary material for this manuscript includes the following:
Supplementary Data 1 and Table 1
Supplementary Data 1. List of expressed genes for each cluster in the stomach and the intestine reported in Figure 1a -d.
Supplementary Table 1. List of primers used in this study.

SUPPLEMENTARY METHODS

Immunogold transmission electron microscope (ImmunoTEM)

Ng2$^{+/DsRed}$ mice were used for immunoelectron microscopy according to the previously reported protocol [1]. Briefly, the ileum was dissected and fixed overnight at room temperature with 4% paraformaldehyde/0.1 M phosphate buffer, pH 7.4, 0.1% glutaraldehyde. After cryo dehydration at -20C using ethanol series and embedding in LR White, sections of 70 nm were cut on a Leica Ultracut and applied to formvar/carbon-coated Nickel grids. Immunogold staining was performed as described above, using anti-RFP (600-401-379, Rockland Inc, 1:300) with overnight antibody incubation at 4 degree followed by 1 hour incubation with donkey Anti-Rabbit IgG H&L (25nm Gold) (ab41514, Abcam, 1:20) at room temperature. Specimens were observed in a FEI Tecnai T20 TEM at 120kV, and images were recorded with a digital camera (Morada), using iTEM software (Soft Imaging System).

Random forest based similarity learning for scRNA-seq data

We used random forest based similarity learning (RAFSIL) to learn cell–cell similarities of different cell types in each tissue and verify the clustering result from the SNN algorithm. RAFSIL implements a two-step procedure, where feature construction geared towards scRNA-seq data is followed by similarity learning. We chose the top 20 marker genes of each cluster as input features. We modified RAFSIL implementation by pooling data from every 20 cells randomly selected within each cell type and tissue, then applying RAFSIL to the average gene expression profile of the pooled data. We also replaced the randomForest() in RAFSIL with randomForest() in paralleRandomForest. These two simple modifications effectively increase data quality, while reducing the computational burden.

paralleRandomFores: https://bitbucket.org/mkuhn/parallelrandomforest

Mass cytometry (CyTOF)

Mass cytometric analysis of 12 cell surface antibodies and one intracellular antibody was performed, using a panel which includes Ly6c-115In (128002, HK1.4, Biolegend, 1:300), CD44-141Pr (103002, IM7, Biolegend, 1:800), Anti-Lyve-1-PE (D225-5, ALY7, MBL international, 1:300), Anti-PE-45Nd (408202, PE001, Biolegend, 1:100), αSMA-146Nd (14-9760-82, 1A4, Invitrogen, 1:500), CD24-150Nd (101829, M1/69, Biolegend, 1:800), PDGFRβ-151Eu (136002

APB5, Biolegend, 1:50), PDGFRα-156Gd (14-1401-82, APA5, Biolegend, 1:300), Sca1-164Dy (3164005B, D7, Fluidigm, 1:200), CD31-165Ho (102425, 390, Biolegend, 1:800), CD9-166Er (NBP1-44876, EM-04, Novusbio, 1:300), CD177-169Tm (553352, 2B8, BD Biosciences, 1:300), CD34-173Yb (119302, MEC14.7, Biolegend, 1:100), Anti-PDPN-FITC (127415, 8.1.1, BioLegend, 1:300), Anti-FITC-174Yb (3174006B, FIT22, Fluidigm, 1:200).

Bapx1$^{+/Cre}$;*Rosa26*$^{+/tdTomato}$ and *Ng2-Cre*;*Rosa26*$^{+/tdTomato}$ reporter mice were used for stomach and intestinal mesenchymal cell isolation. Single cell isolation and sorting were performed as previously described in the scRNA-sequencing method section. Sorted cells were washed once in CyTOF staining media (CSM) (PBS with 1% BSA), collected by centrifugation for 5 minutes at 300g and supernatant aspirated. Fc receptors were blocked by re-suspending the cell pellets in 25 uL (per $3x10^6$ cells) of 2X TruStain (BioLegend, California, USA) for 10 minutes at RT. Abs specific for surface markers were diluted to 2X the desired final concentration in CSM. An equal volume of this cocktail was added to the cells without washing out the TruStain, and were incubated for 30 minutes at RT. Cells were then washed with 3mL of CSM, collected by centrifugation for 5 minutes at 300g. Secondary markers were diluted to 1X the desired final concentration in CSM and 50uL was added to cells and incubated for 30 minutes at RT. Cells were washed with 3mL PBS and collected by centrifugation for 5 minutes at 300g and supernatant aspirated. Cell pellets were resuspended in 2X Cisplatin viability stain (1:1 in PBS) and incubated for 5 minutes at RT and promptly quenched by adding 2mL CSM. Cells were collected by centrifugation for 5minutes at 300g and were fixed and permed using BD Cytofix and Cytoperm (BD Biosciences) as per manufacturer's protocol. Intracellular antibodies were diluted to 1X in Cytoperm buffer and 50uL were added to cells and incubated for 30 minutes at RT. Cells were then washed with 3mL CSM and collected by centrifugation for 5 minutes at 600g and supernatant aspirated. Cells were stained with 1mL of 100nM Iridium nucleic acid intercalator (Fluidigm) overnight at 4C. Cells were then washed once with CSM and once with PBS. Cell pellets were resuspended in Fluidigm Cell Acquisiton Solution containing 5-element EQ normalization beads according to Fluidigm's protocol. Data were collected by Helios instrument and converted to FCS files. The FCS files were normalized using the global EQ bead passport value by the Helios software.

Cell type specific expression of disease-associated genes

Using normalized expression values, we obtained a z-score for each cell, then computed the mean z-score for each cluster. Genes that display any cluster specific-enrichment ($z > 1.65$) were kept for further analysis. We acquired disease-associated SNPs and their neighbouring genes from the NHGRI-EBI catalog (https://www.ebi.ac.uk/gwas/)[2] and plotted the z-transformed expression of genes for each disease term individually. Genes were clustered using hclust function (R package: stats) with default parameters, while cell clusters were plotted in the order described above.

Supplementary Figure 1. related to figure 1

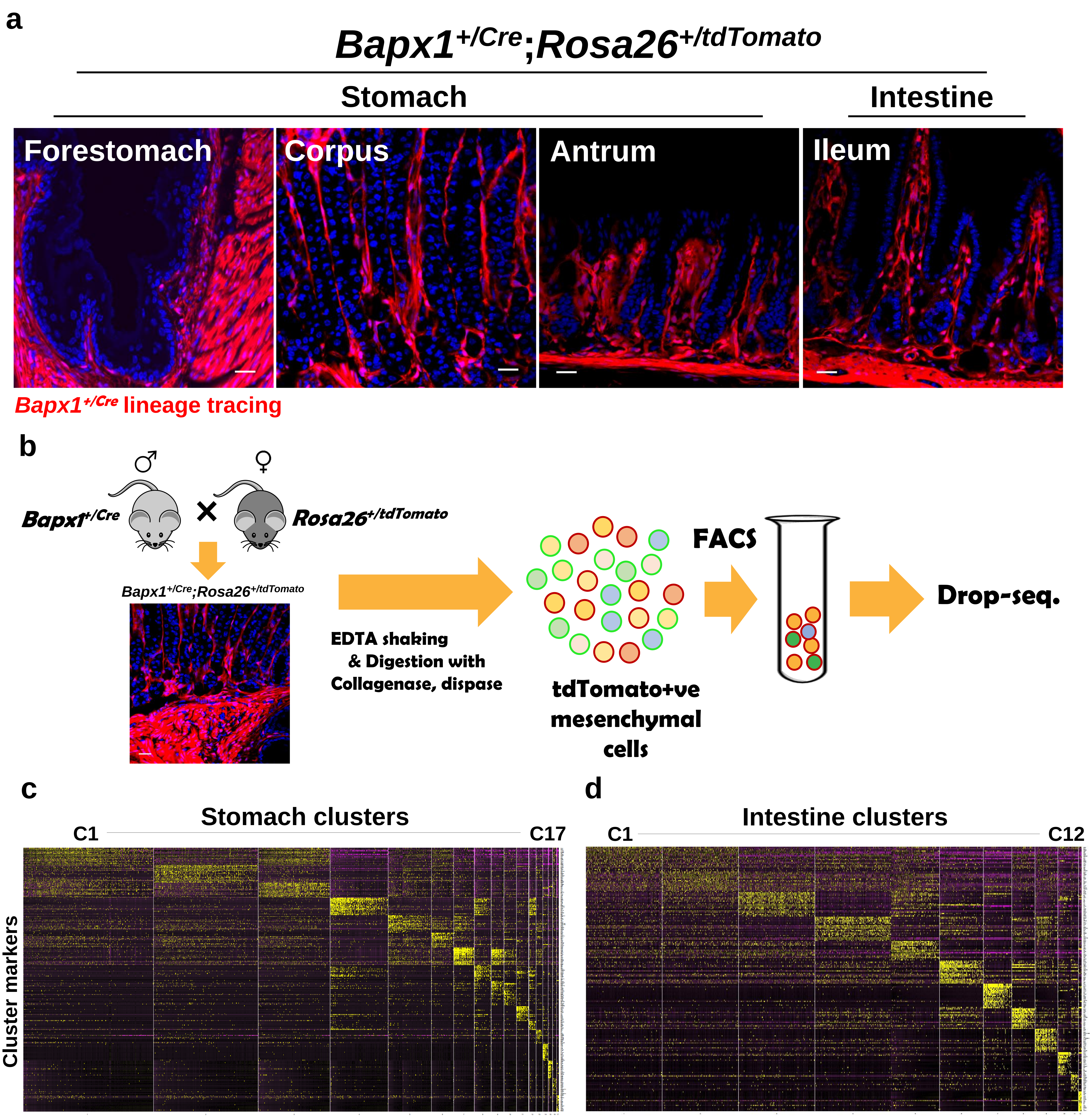

Supplementary Figure 1. Overview of single-cell RNA-seq (related to Figure 1).
(a) tdTomato expression in the gastrointestinal tract of *Bapx1*$^{+/Cre}$;*Rosa26*$^{+/tdTomato}$ mice demonstrates broad labeling of stomach and intestinal stromal cells. (b) A schematic diagram of gastrointestinal stromal single-cell transcriptomics. (c, d) Heatmaps show the expression of top marker genes for each stomach and intestinal stromal cell cluster. Two mice were pulled for stomach and intestinal scRNA-seq.

Supplementary Figure 2. related to figure 1

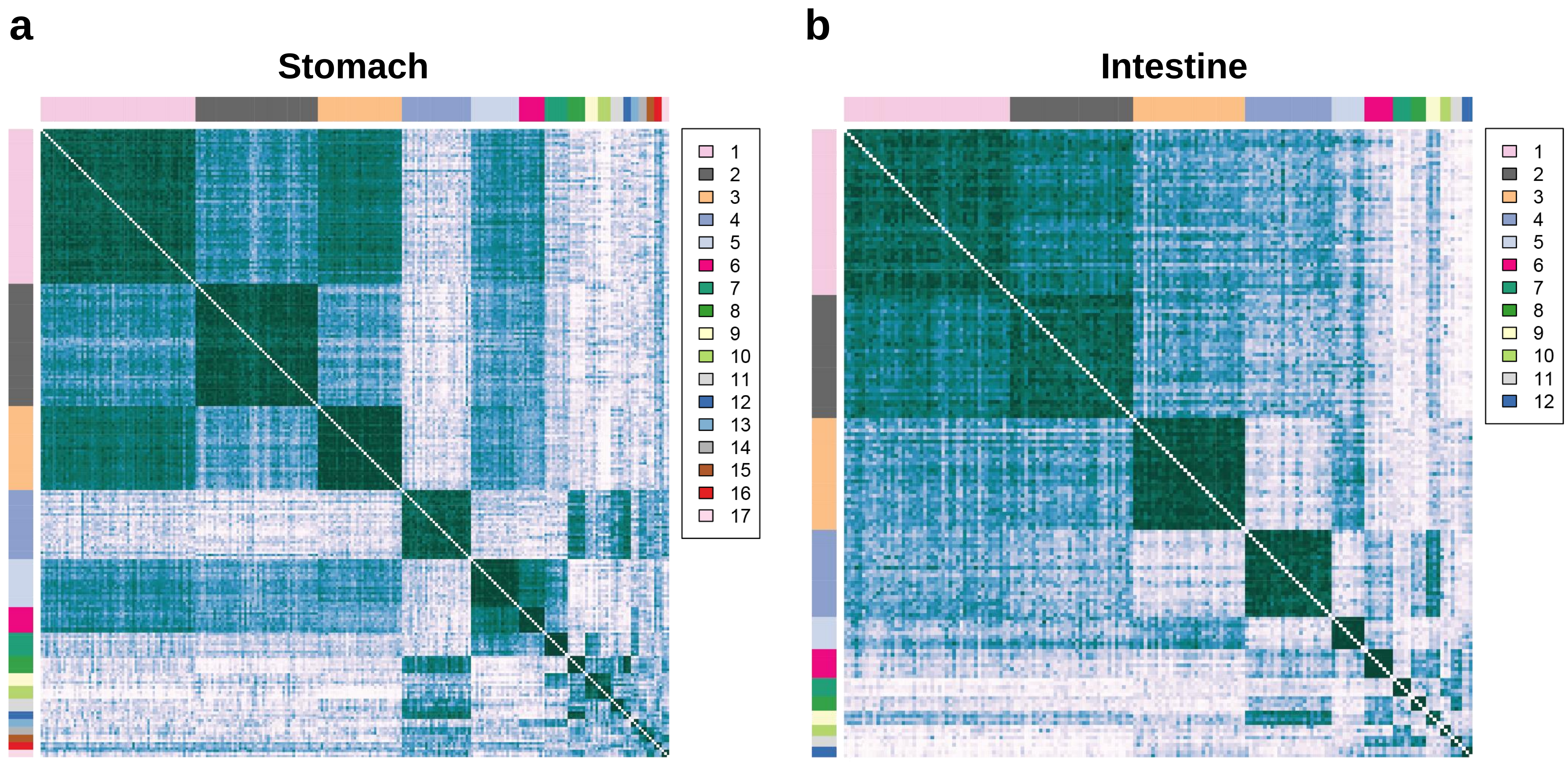

Supplementary Figure 2. Random forest based similarity learning (RAFSIL) for single-cell RNA-seq data (related to Figure 1).

(a, b) Random forest based similarity learning analysis of stomach (a) and intestinal (b) scRNA-seq data for classification.

Supplementary Figure 3. related to figure 1b and 1d

a Stomach

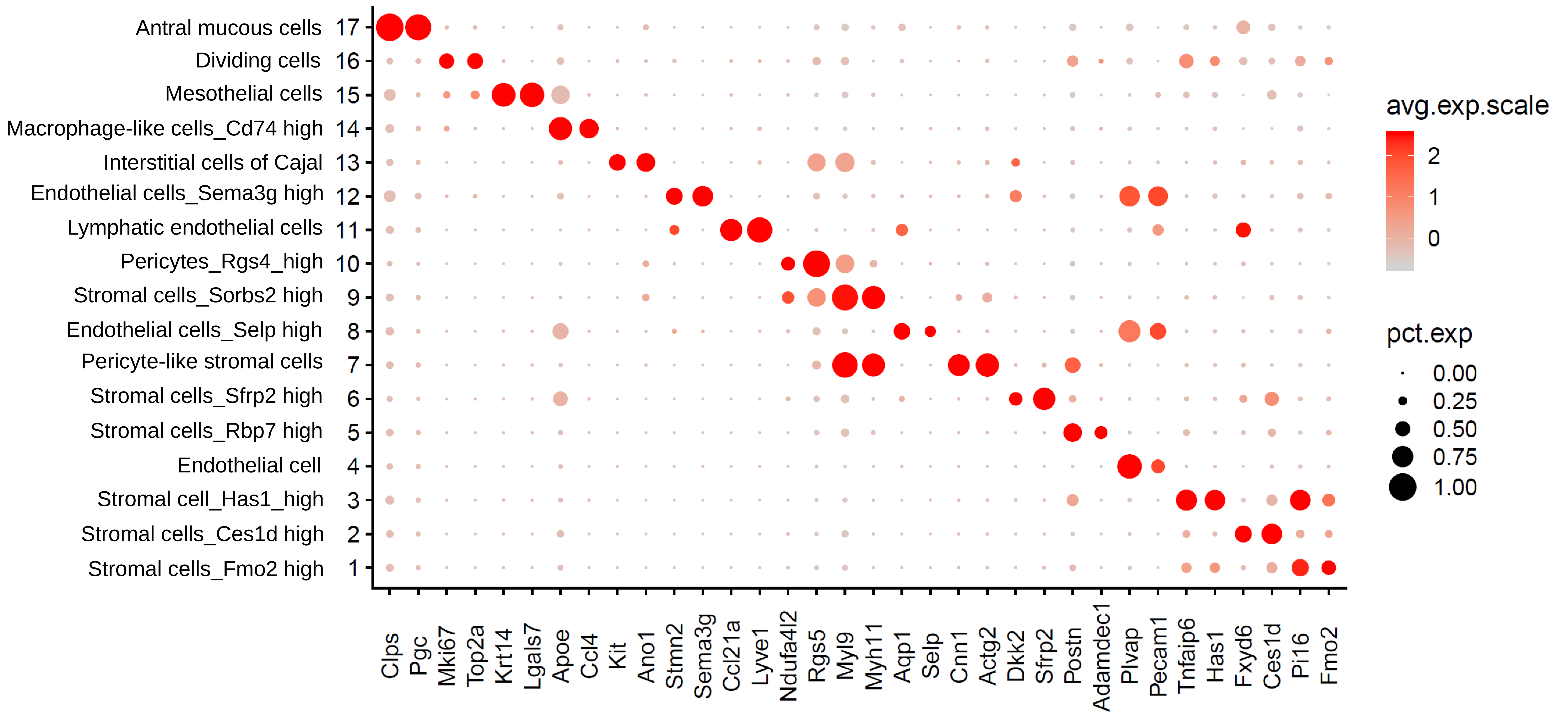

b Intestine

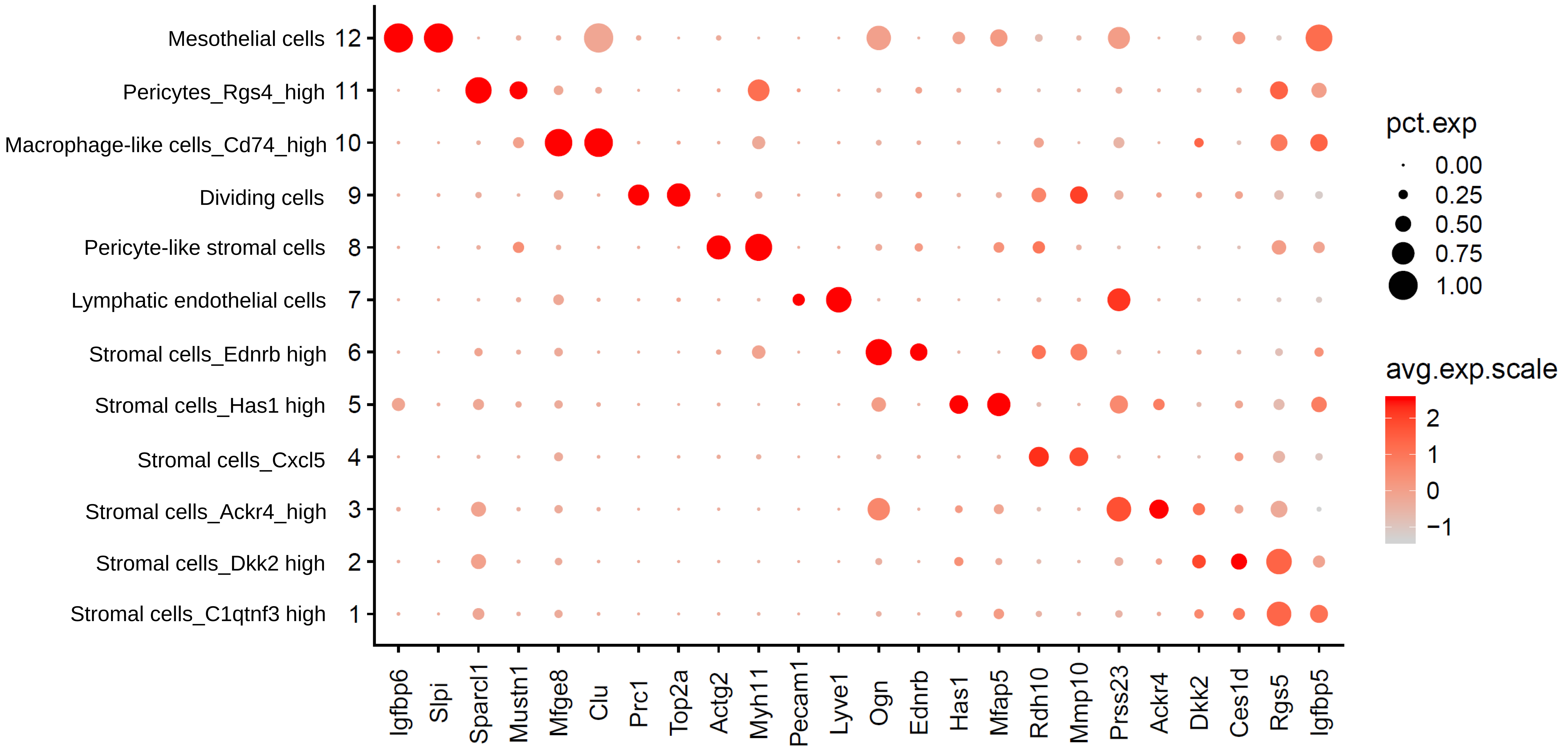

Supplementary Figure 3. Dot plots with representative, differentially expressed markers of single-cell RNA-seq data (related to Figure 1b and 1d).
(a, b) Dot plots of stomach (a) and intestinal (b) scRNA-seq data for each cluster.

Supplementary Figure 4. related to figure 1

a

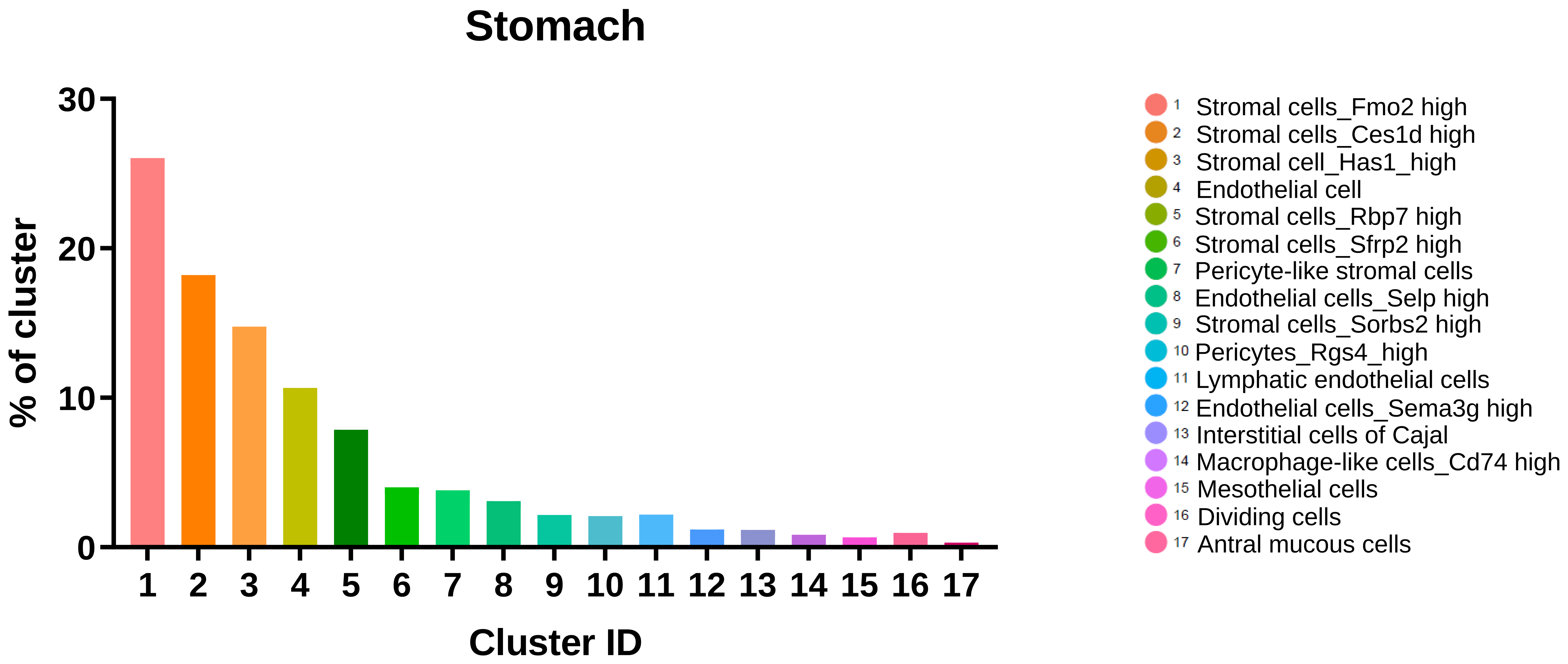

Bapx-scRNA-seq	Stomach																
Cluster ID	1	2	3	4	5	6	7	8	9	10	11	12	13	14	15	16	17
# of cells	1288	901	730	527	389	199	188	153	107	103	108	59	57	42	33	47	15
%	26	18.2	14.8	10.7	7.86	4.02	3.8	3.09	2.16	2.08	2.18	1.19	1.15	0.85	0.67	0.95	0.3

b

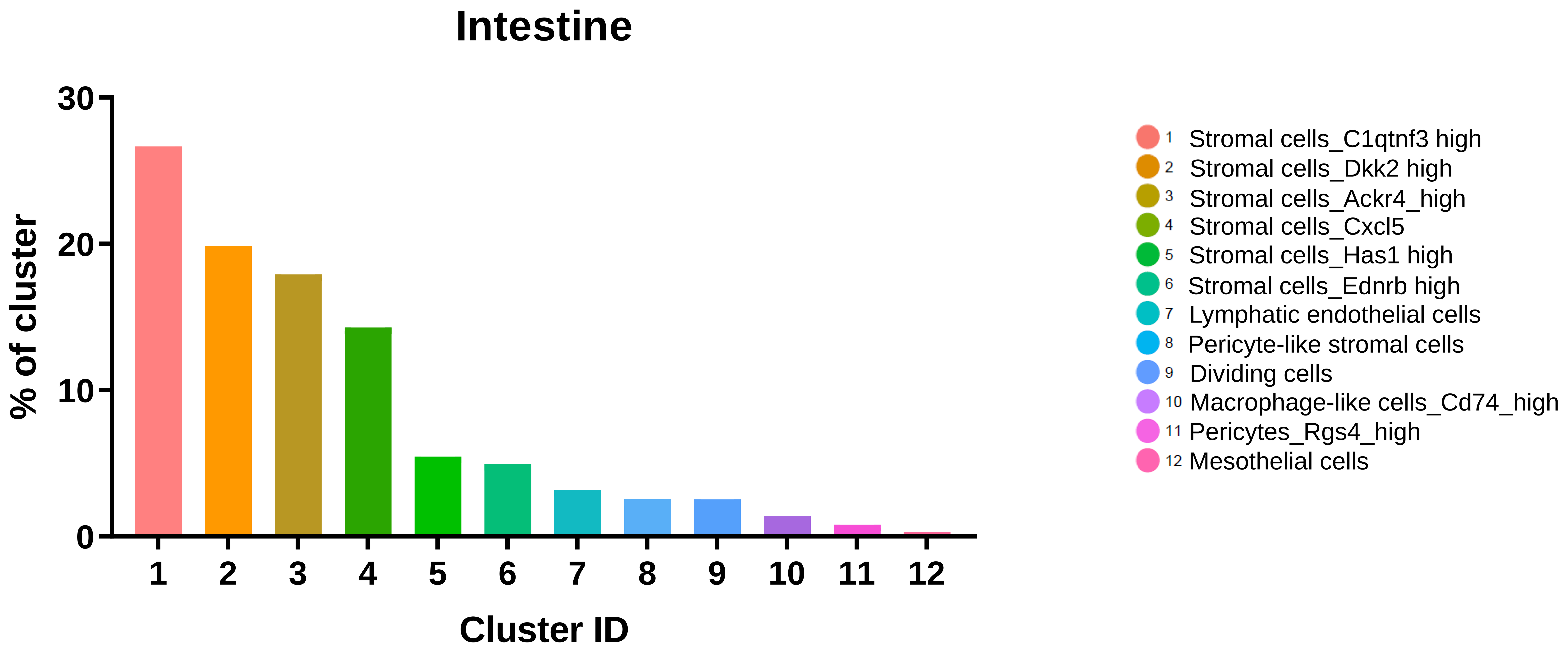

Bapx-scRNA-seq	Intestine											
Cluster ID	1	2	3	4	5	6	7	8	9	10	11	12
# of cells	922	687	620	494	189	172	110	89	88	49	28	11
%	26.66	19.86	17.92	14.28	5.46	4.97	3.18	2.57	2.54	1.42	0.81	0.32

Supplementary Figure 4. Percentage of each cluster in scRNA-seq data (related to Figure 1).
(a, b) Proportion of each cluster in scRNA-seq data among 4946 stomach cells (a) and 3459 intestinal cells (b).

Supplementary Figure 5. related to figure 1e

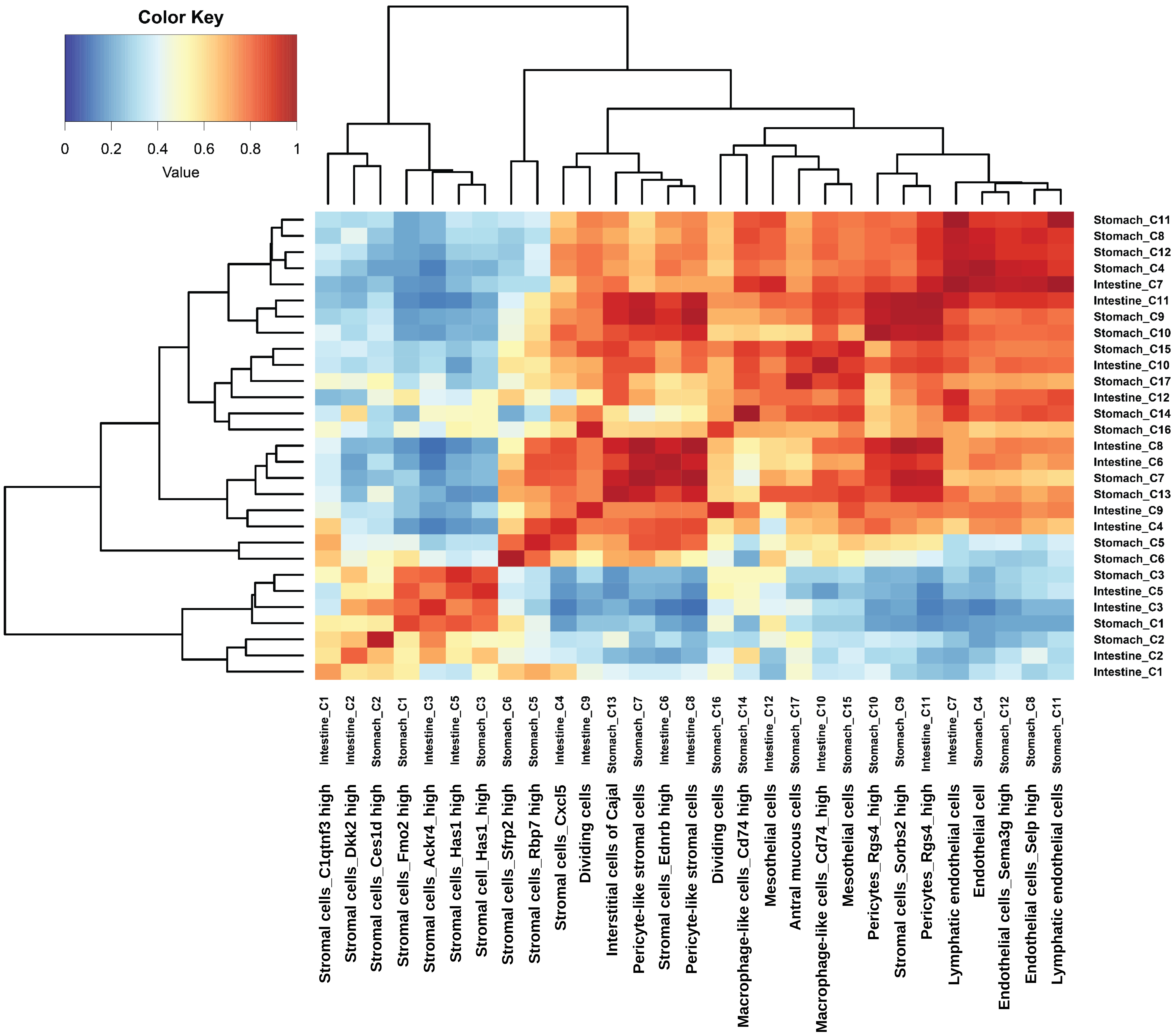

Supplementary Figure 5. Unsupervised hierarchical clustering including correlation matrices to compare stomach and intestinal clusters (related to Figure 1e).

Unsupervised hierarchical clustering shows gene expression correlation between the stomach and intestinal stromal cell clusters using area under curve (AUC) scores calculated from MetaNeighbor.

Supplementary Figure 6. related to figure 1e

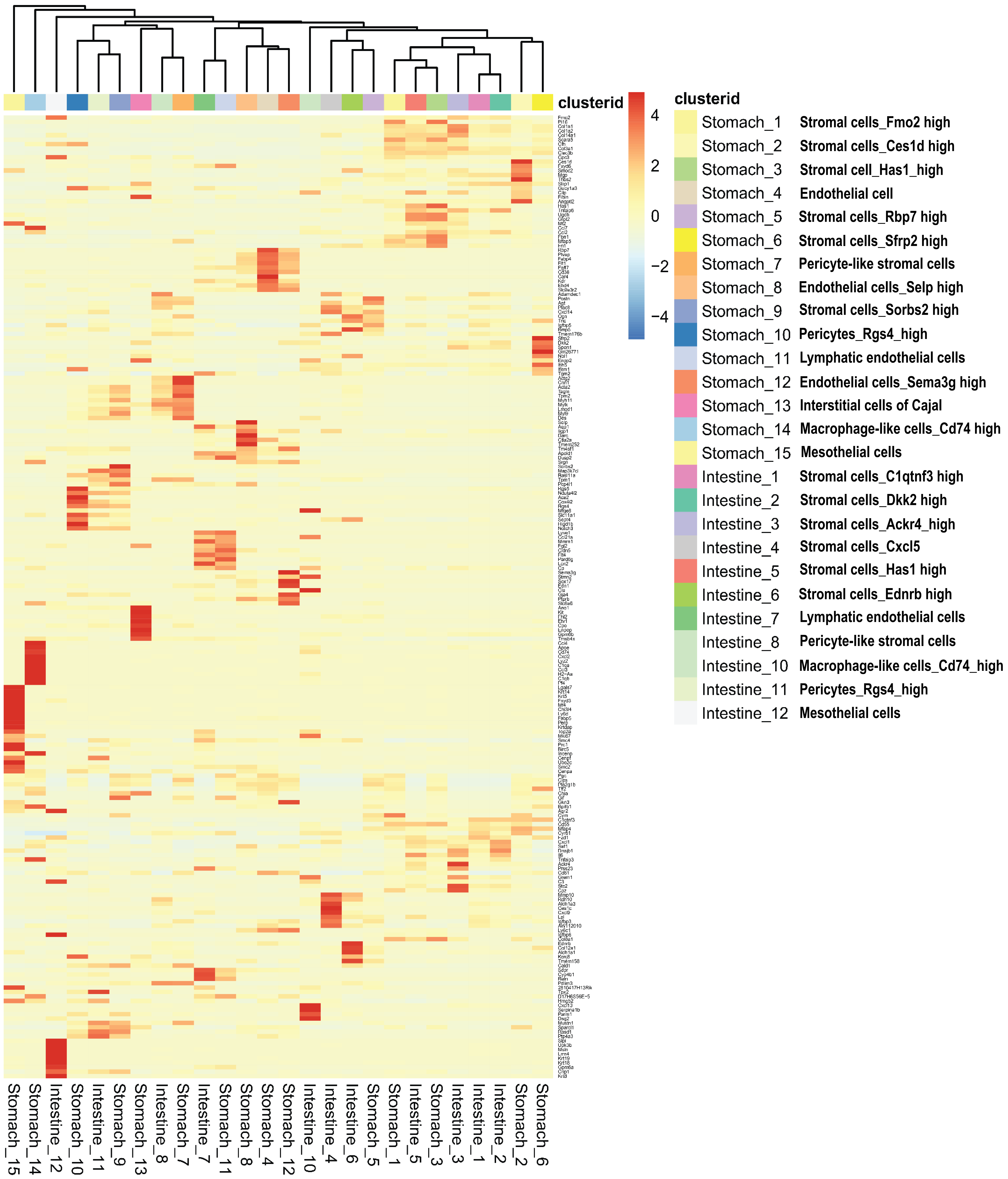

Supplementary Figure 6. Unsupervised hierarchical clustering including representative cluster markers to compare stomach and intestinal clusters (related to Figure 1e).

Unsupervised hierarchical clustering including representative markers for each cluster in y-axis.

Supplementary Figure 7. related figure 1

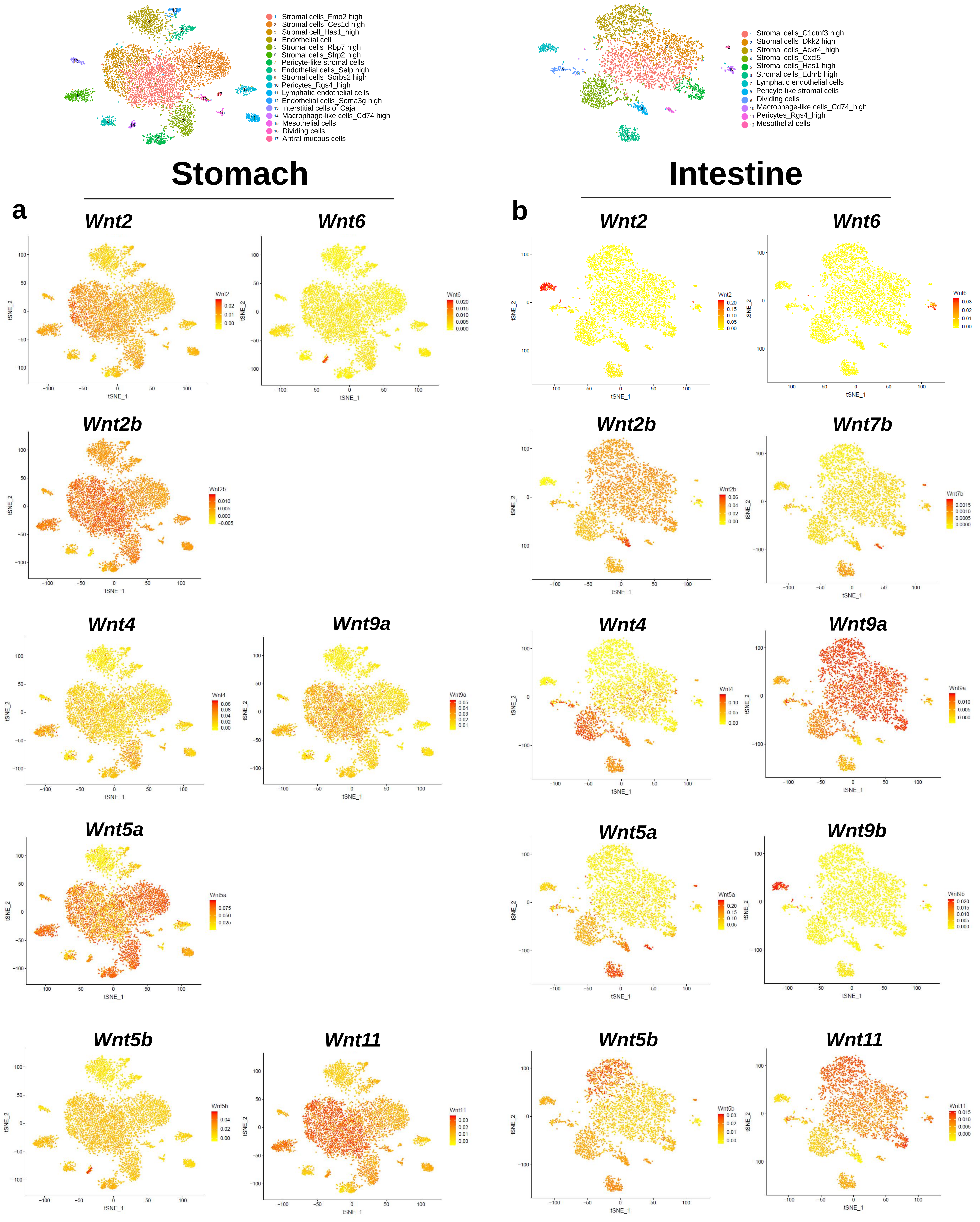

Supplementary Figure 7. Expression of Wnt ligand genes in gastrointestinal stromal cells (related to Figure 1). (a, b) t-SNE plots showing the relative expression levels of *Wnt2*, *Wnt2b*, *Wnt4*, *Wnt5a*, *Wnt5b*, *Wnt6*, *Wnt7b*, *Wnt9a*, *Wnt9b*, and *Wnt11* in stomach (a) and intestinal (b) stromal cell clusters using MAGIC. *Wnt7b* and *Wnt9b* were not detected in stomach scRNA-seq data.

Supplementary Figure 8. related figure 1

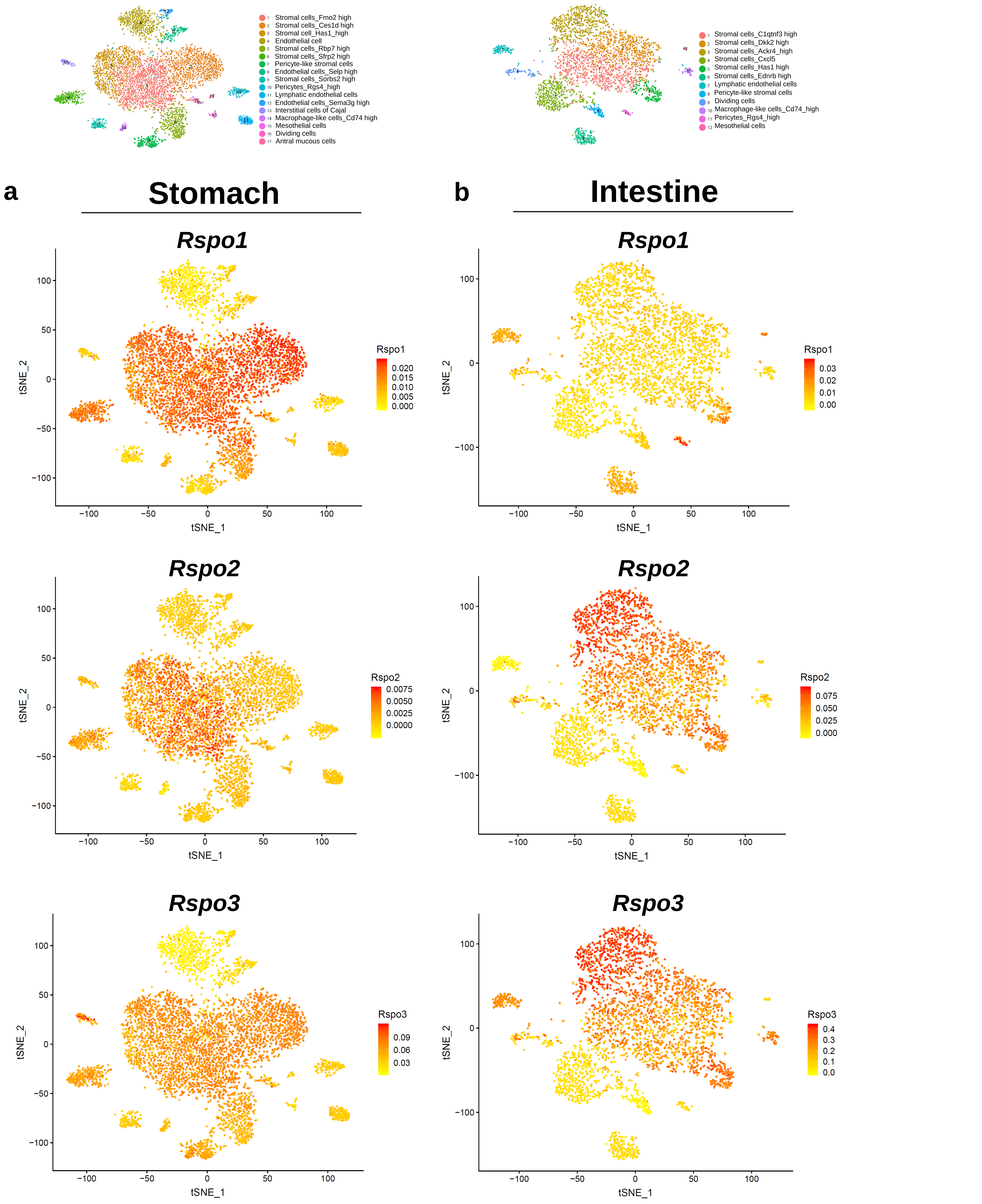

Supplementary Figure 8. Expression of R-spondin genes in gastrointestinal stromal cells (related to Figure 1).

(a, b) t-SNE plots showing the relative expression levels of *Rspo1*, *Rspo2*, and *Rspo3* in stomach (a) and intestinal (b) stromal cell clusters.

Supplementary Figure 9. related figure 1

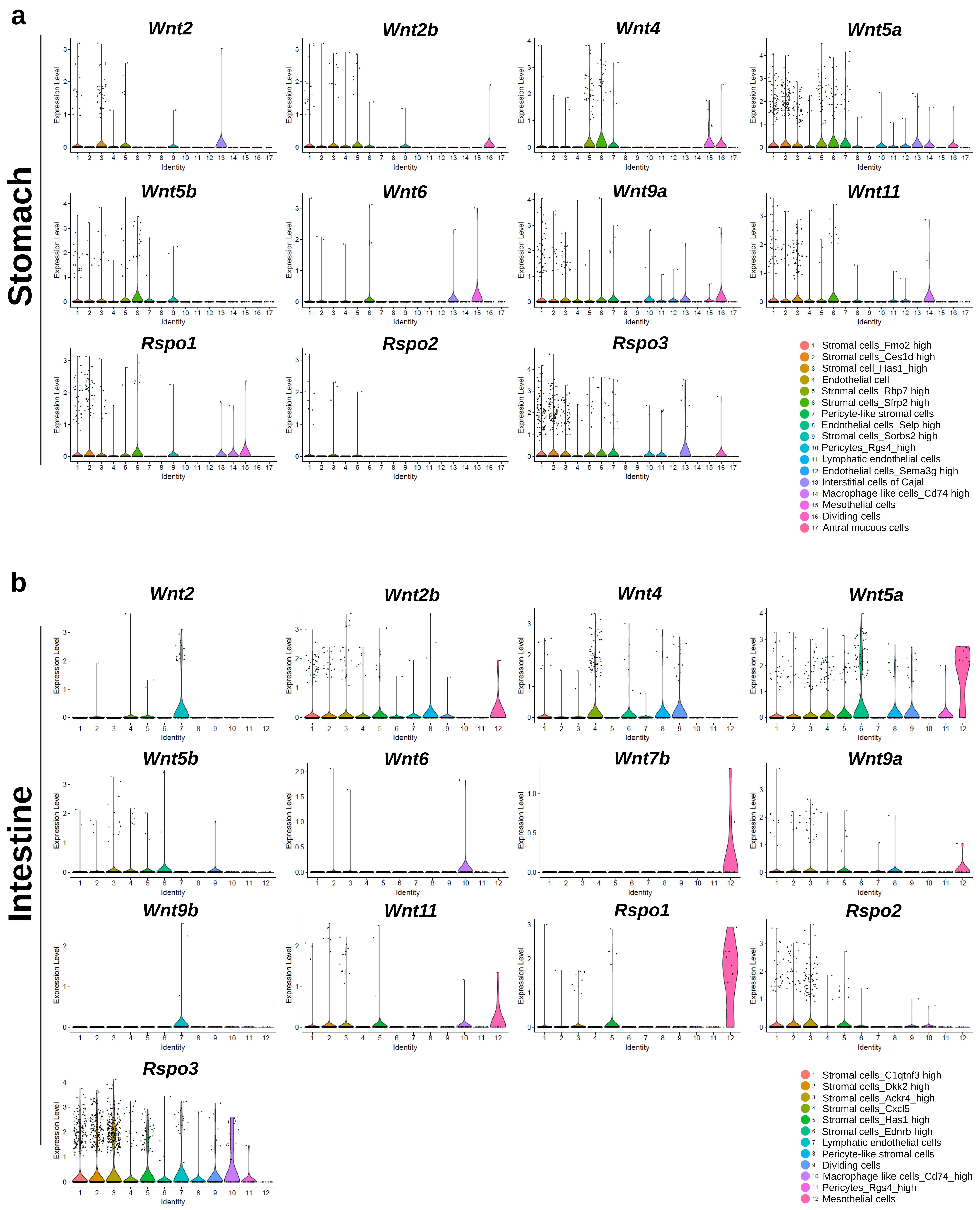

Supplementary Figure 9. Violin plots for Wnt ligands and Rspo family in stomach and intestial stromal cells (related to Figure 1).

(a, b) Violin plots of *Wnt2*, *Wnt2b*, *Wnt4*, *Wnt5a*, *Wnt5b*, *Wnt6*, *Wnt7b*, *Wnt9a*, *Wnt11*, *Rspo1*, *Rspo2* and *Rspo3* in the stomach (a) and *Wnt2*, *Wnt2b*, *Wnt4*, *Wnt5a*, *Wnt5b*, *Wnt6*, *Wnt7b*, *Wnt9a*, *Wnt9b*, *Wnt11*, *Rspo1*, *Rspo2 and Rspo3* in the intestine (b). *Wnt7b* and *Wnt9b* were not detected in stomach scRNA-seq data.

Supplementary Figure 10. related figure 1

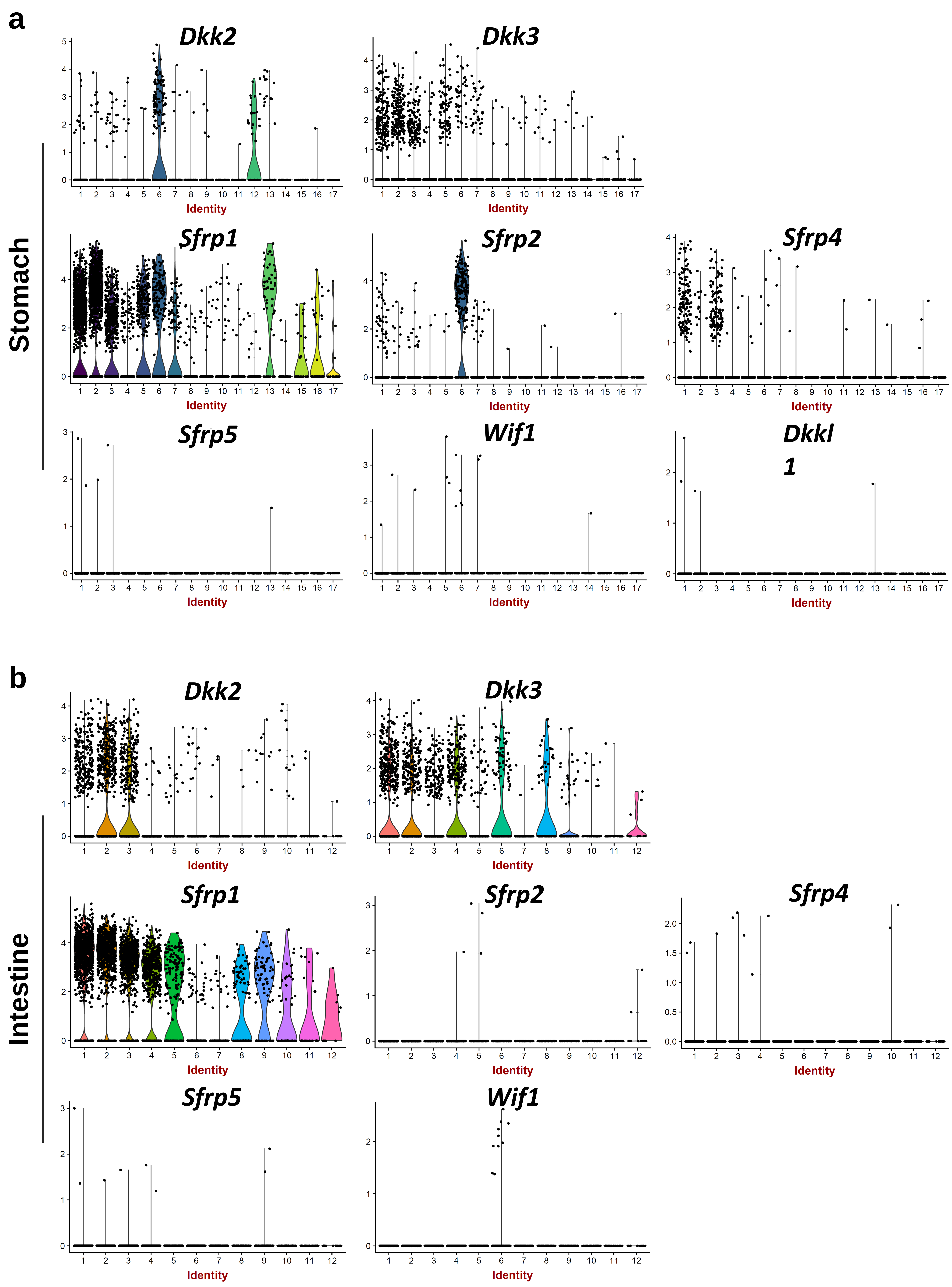

Supplementary Figure 10. Violin plots of Wnt antagonists in the stomach and intestine (related to Figure 1).

(a, b) Violin plots for Wnt antagonists (*Dkk2, Dkk3, Sfrp1, Sfrp2, Sfrp4, Sfrp5, Wif1 and Dkkl1*) in the stomach (a) and intestine (b).

Supplementary Figure 11. related figure 1

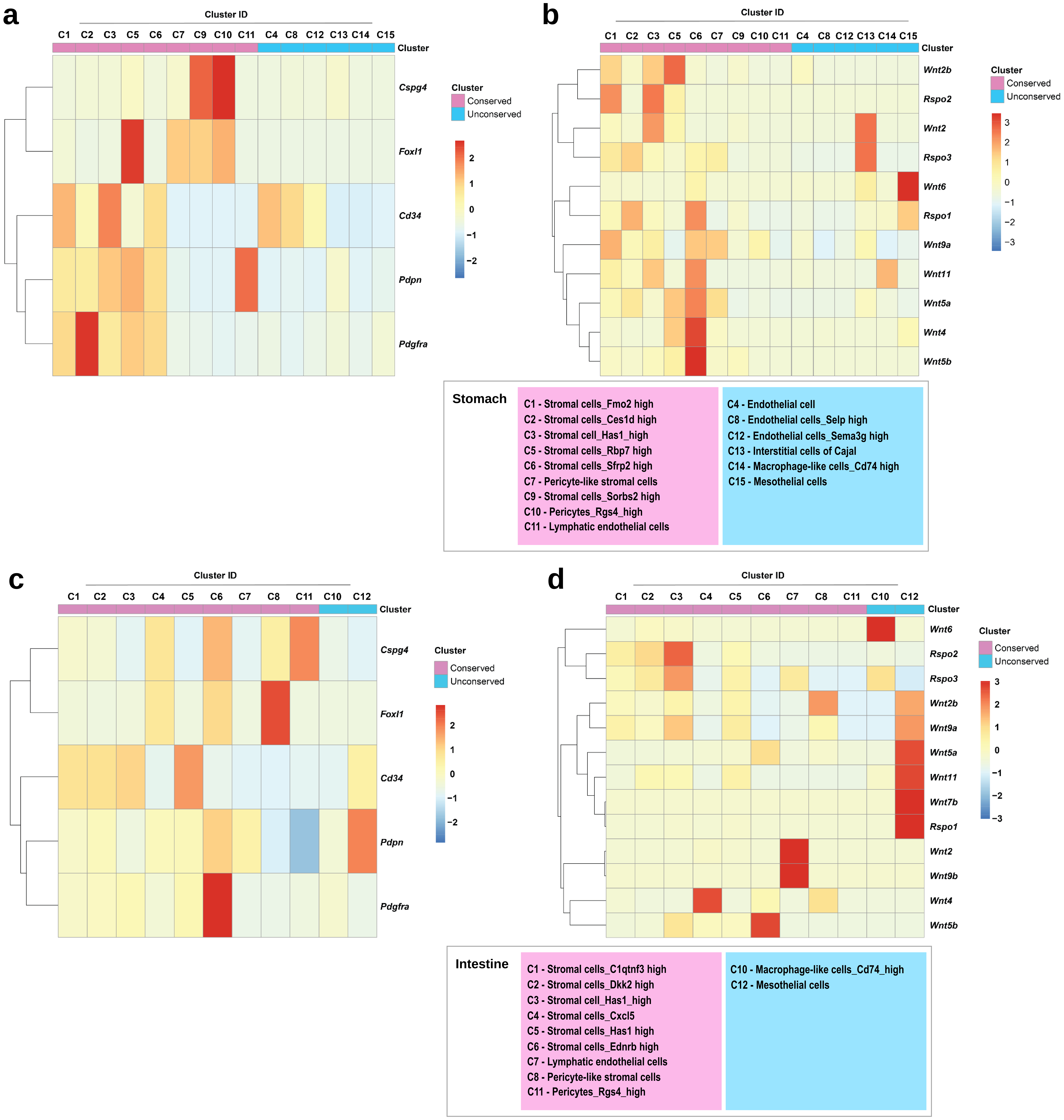

Supplementary Figure 11. Heatmap of stromal cell markers and Wnt ligands in the conserved and unconserved stomach and intestinal clusters (related to Figure 1).

(a, c) Heatmaps show the relative expression levels of pericyte markers, *Cspg4*, *Foxl1*, *Cd34*, *Pdpn*, and *Pdgfrα*, in the stomach (a) and intestine (c). (b, d) Heatmaps show the expression of Wnt ligands and R-spondin family in the stomach (b) and intestine (d). Pink colored clusters indicate the conserved population, while light blue colored clusters indicate the unconserved clusters between the stomach and intestine. Red indicates high relative levels of expression for marker genes in clusters, while blue indicates low relative levels of gene expression.

Supplementary Figure 12. related figure 1 and 2

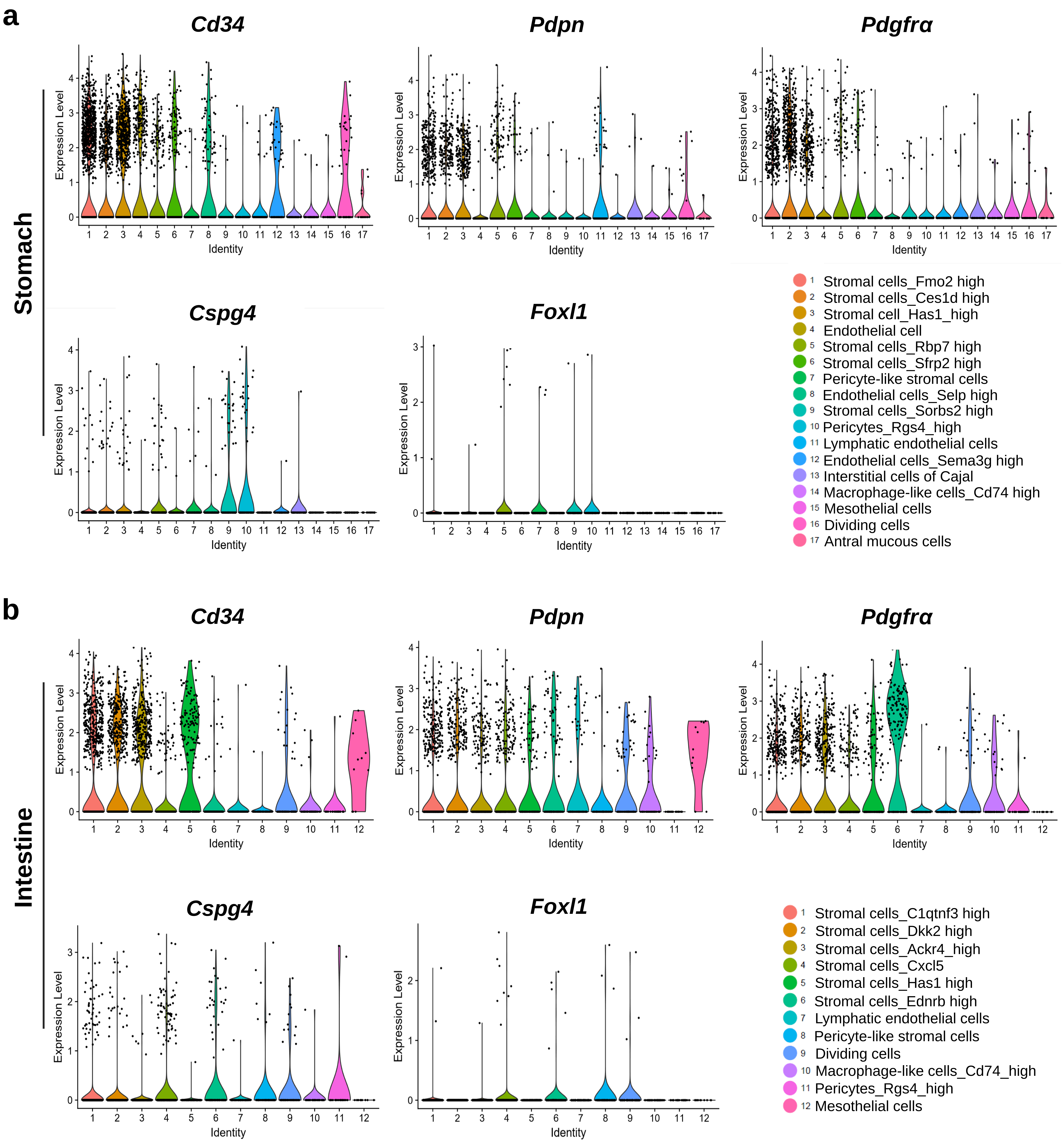

Supplementary Figure 12. Violin plots of pericyte and intestinal pericryptal cell markers in the stomach and intestine (related to Figure 1).

(a, b) Violin plots for pericyte-like cell markers such as *Cd34*, *pdpn*, *Pdgfrα*, *Cspg*4 and *Foxl1* in the stomach (a) and intestine (b).

Supplementary Figure 13. related figure 1

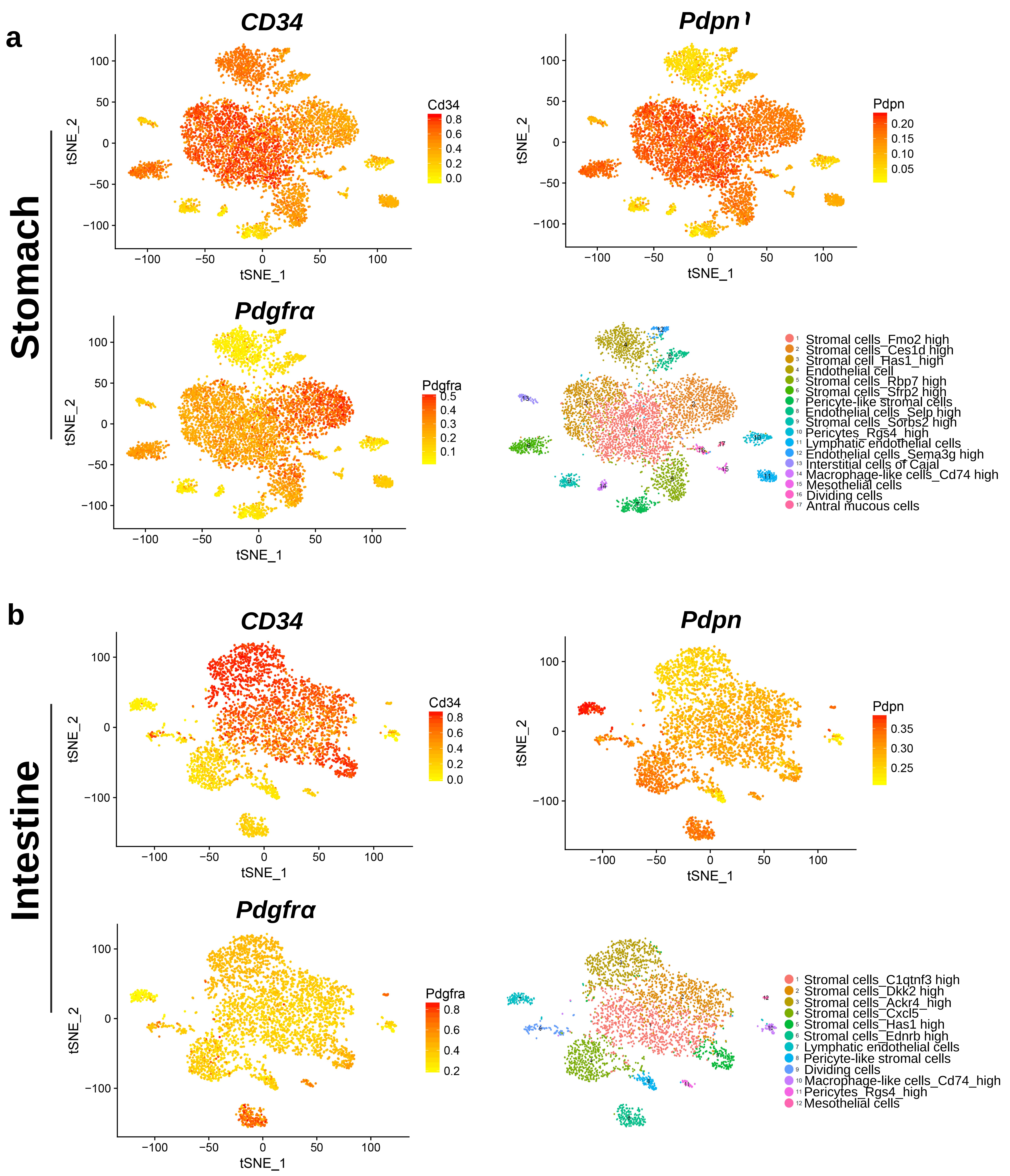

Supplementary Figure 13. Expression of pericyte and intestinal pericryptal cell markers in the stomach and intestine (related to Figure 1).

(a, b) Feature plots show the expression of pericyte markers *Cd34*, *Pdpn*, and *Pdgfrα* in stomach (a) and intestine (b).

Supplementary Figure 14. related to figure 2

a

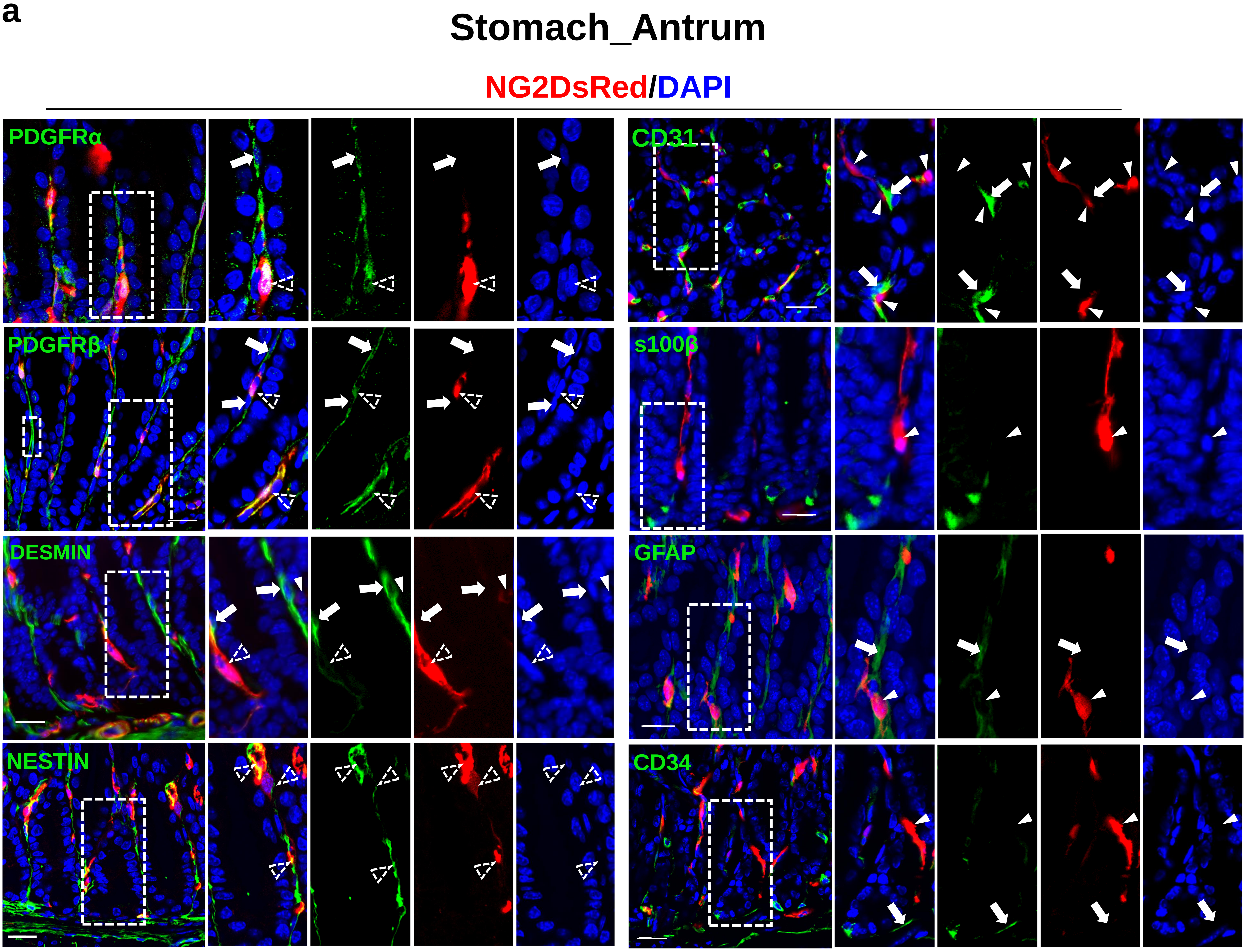

Supplementary Figure 14. continued. related to figure 2

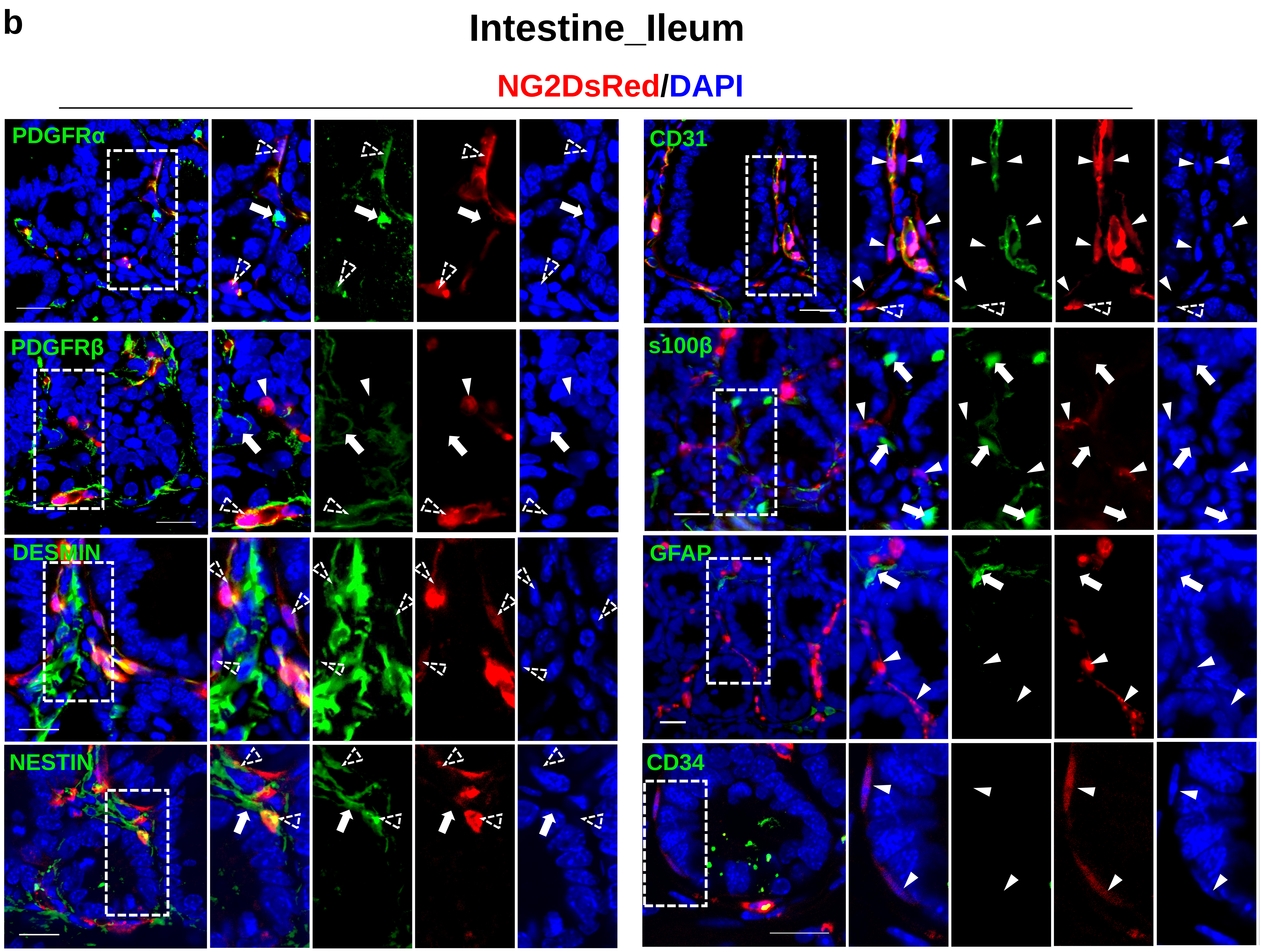

Supplementary Figure 14. Characterization of real-time *Ng2* expressing cells from *Ng2*$^{+/DsRed}$ mice in the antrum (related to Figure 2).

(a, b) Red color shows real-time *Ng2* expressing cells in proximity to epithelial cells in the *Ng2*$^{+/DsRed}$ mouse stomach (a) and intestine (b). Blue is DAPI. Green colors show IF stainings of PDGFRα, PDGFRβ, DESMIN, NESTIN, CD31, s100β, GFAP and CD34 in *Ng2*$^{+/DsRed}$ mice, as indicated in each panel. Empty arrowhead indicates co-expressing cells and filled arrowhead indicates only DsRed positive cells, while arrow indicates green color single positive cells. Sub-population of PDGFRα, PDGFRβ, DESMIN, and NESTIN positive cells are co-labeled with Ng2DsRed (Left panel). In contrast, CD31, s100β, GFAP and CD34 positive cells are not co-labeled with Ng2DsRed (Right panel). Scale bars indicate 20 μm.

Supplementary Figure 15. related to figure 2 and 3

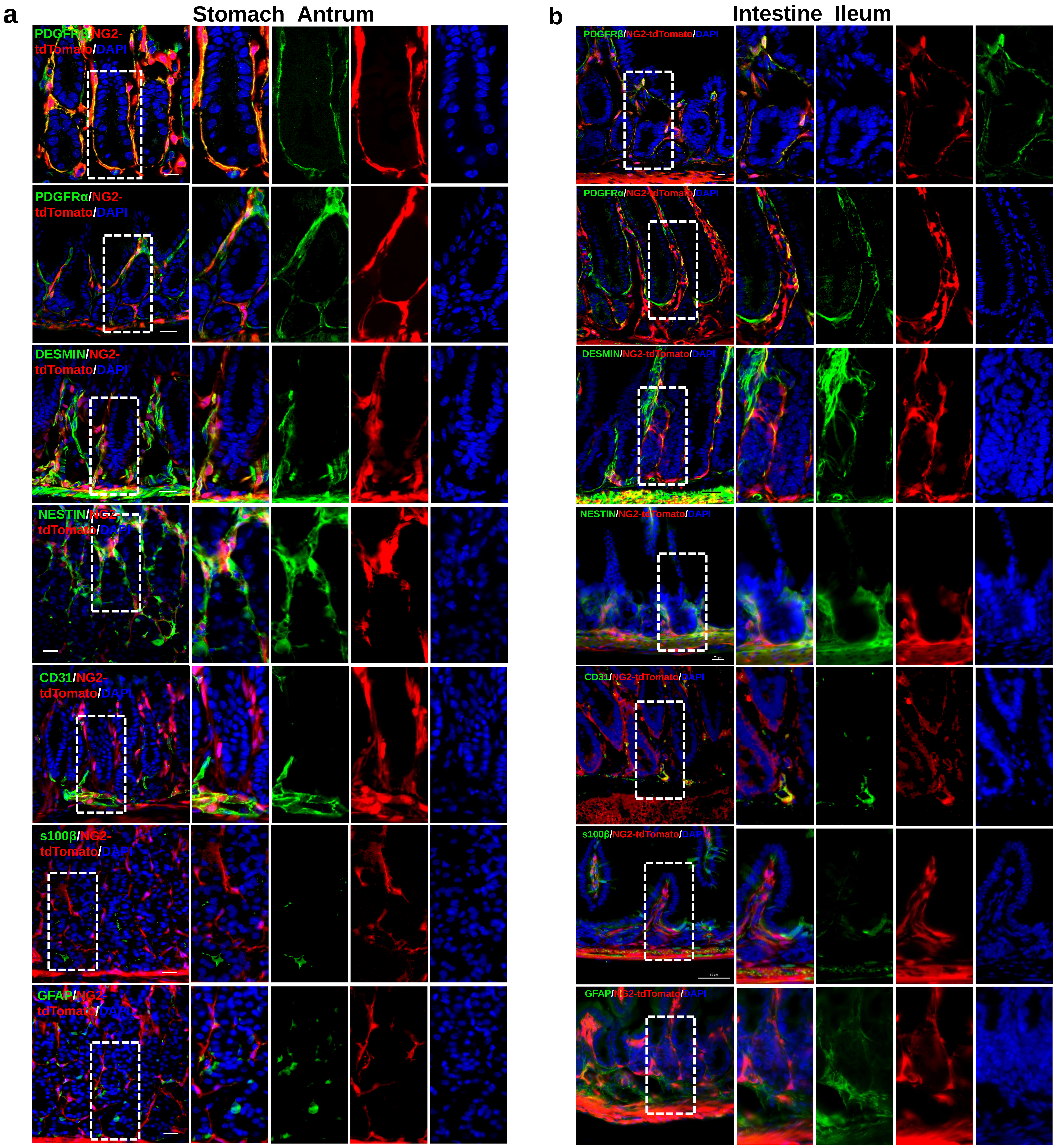

Supplementary Figure 15. Characterization of *Ng2-Cre* lineage traced cells (related to Figures 2 and 3). (a, b) IF of CD31, NESTIN, DESMIN, PDGFRα, PDGFRβ, s100β, αSMA and GFAP in *Ng2-Cre*;*Rosa26*$^{+/tdTomato}$ mice defines pericyte-like stromal cells in proximity to stomach (a) and intestinal (b) epithelial cells. Scale bars indicate

Supplementary Figure 16. related figure 1

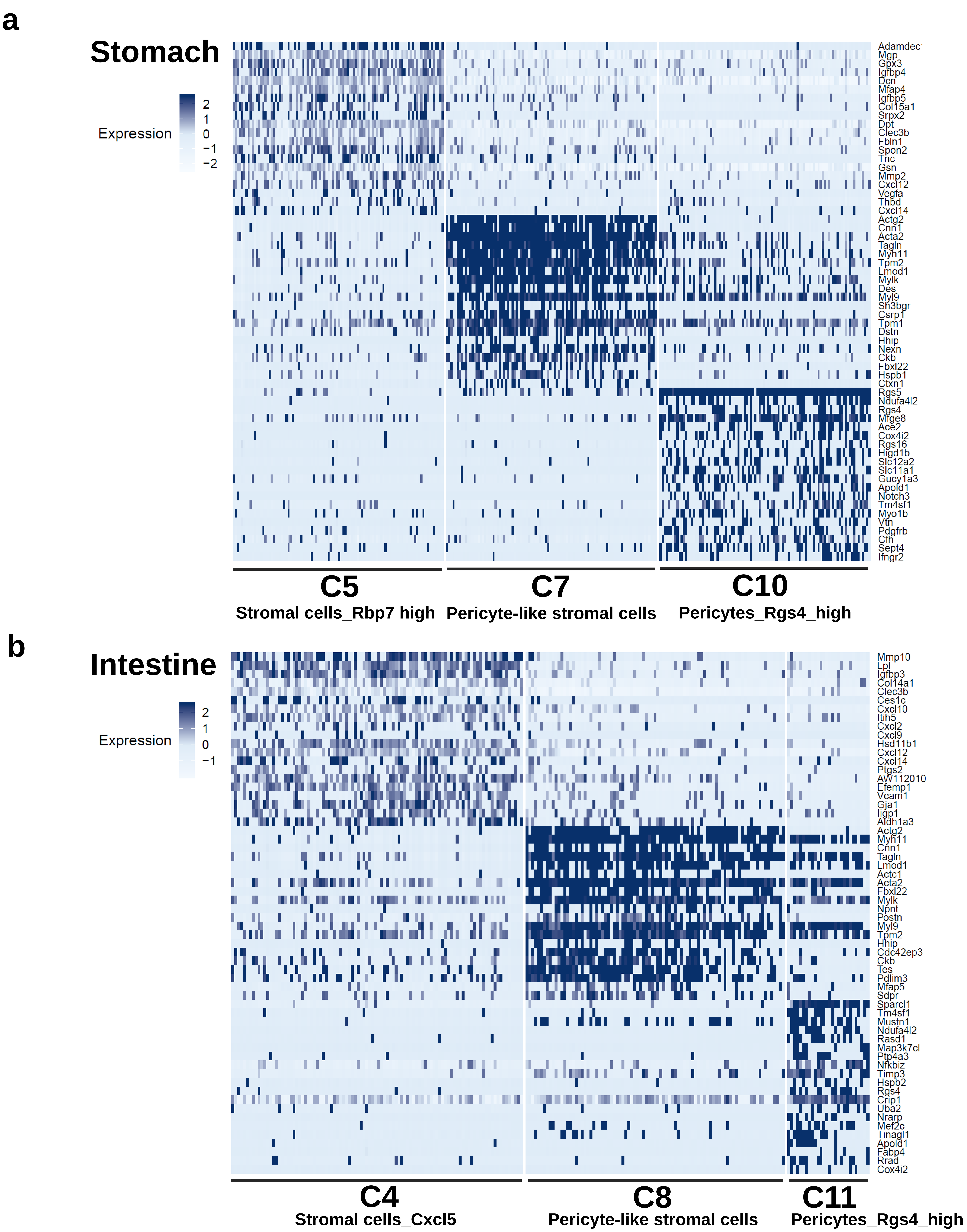

Supplementary Figure 16. Heatmap of differential stromal cell markers in gastrointestinal pericyte-like (telocyte) and pericyte clusters (related to Figure 1).

(a, b) Heatmaps show the expression of marker genes for pericyte-like (telocyte) clusters C5, C7 vs. pericyte cluster C10 in the stomach (a), and pericyte-like (telocyte) clusters C4, C8 vs. pericyte cluster C11 in the intestine (b).

Supplementary Figure 17. related to figure 3a

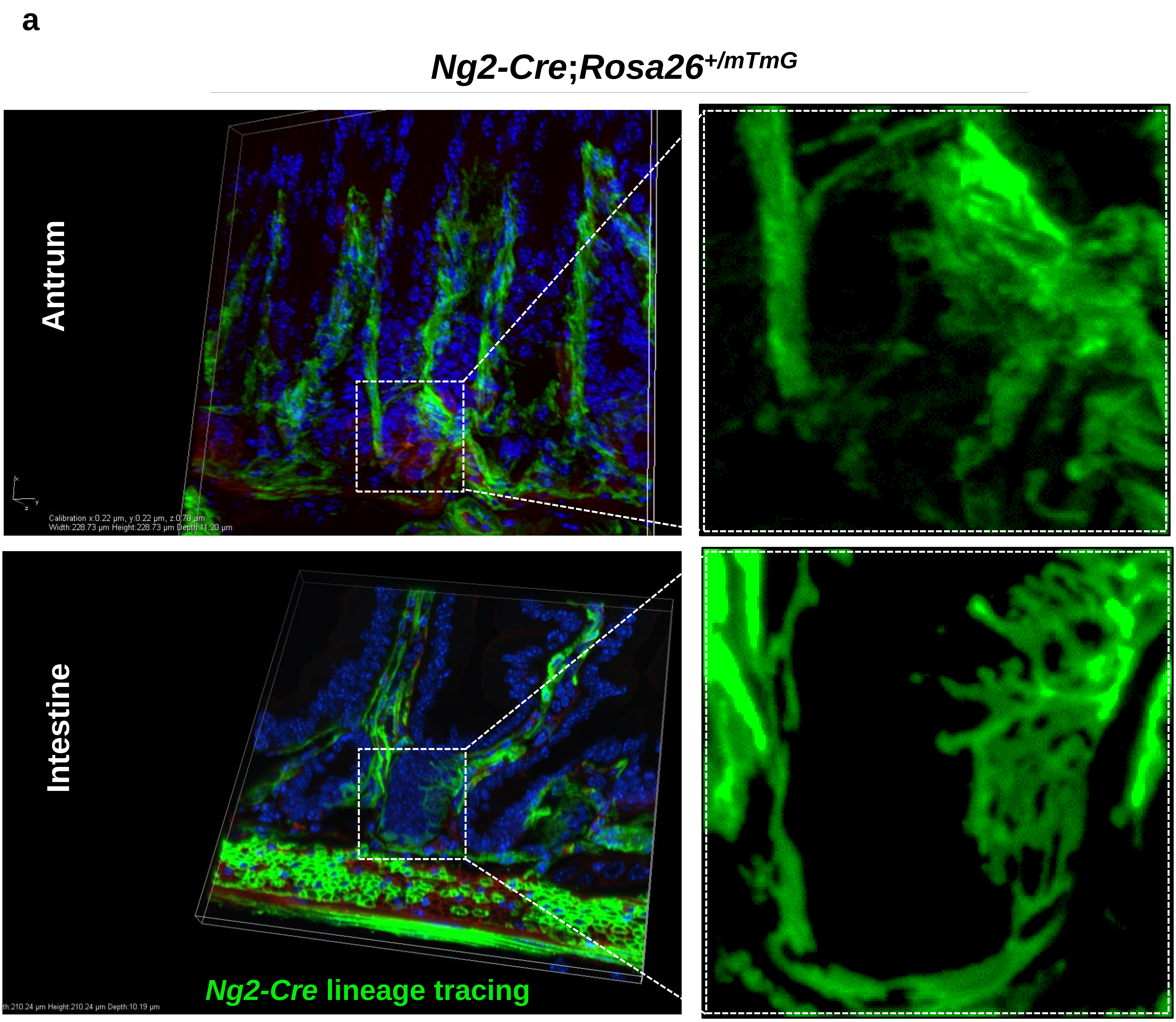

Supplementary Figure 17. Pericyte-like stromal cells surrounding the antral gland and crypt with a mesh-like structure (related to Figure 3a).

3D images of *Ng2-Cre*;*Rosa26*$^{+/mTmG}$ in the antrum and intestine show *Ng2-Cre* lineage labeled (green color) cells surrounding the antral gland and crypt with a thin mesh-like structure.

Supplementary Figure 18. related to figure 3a

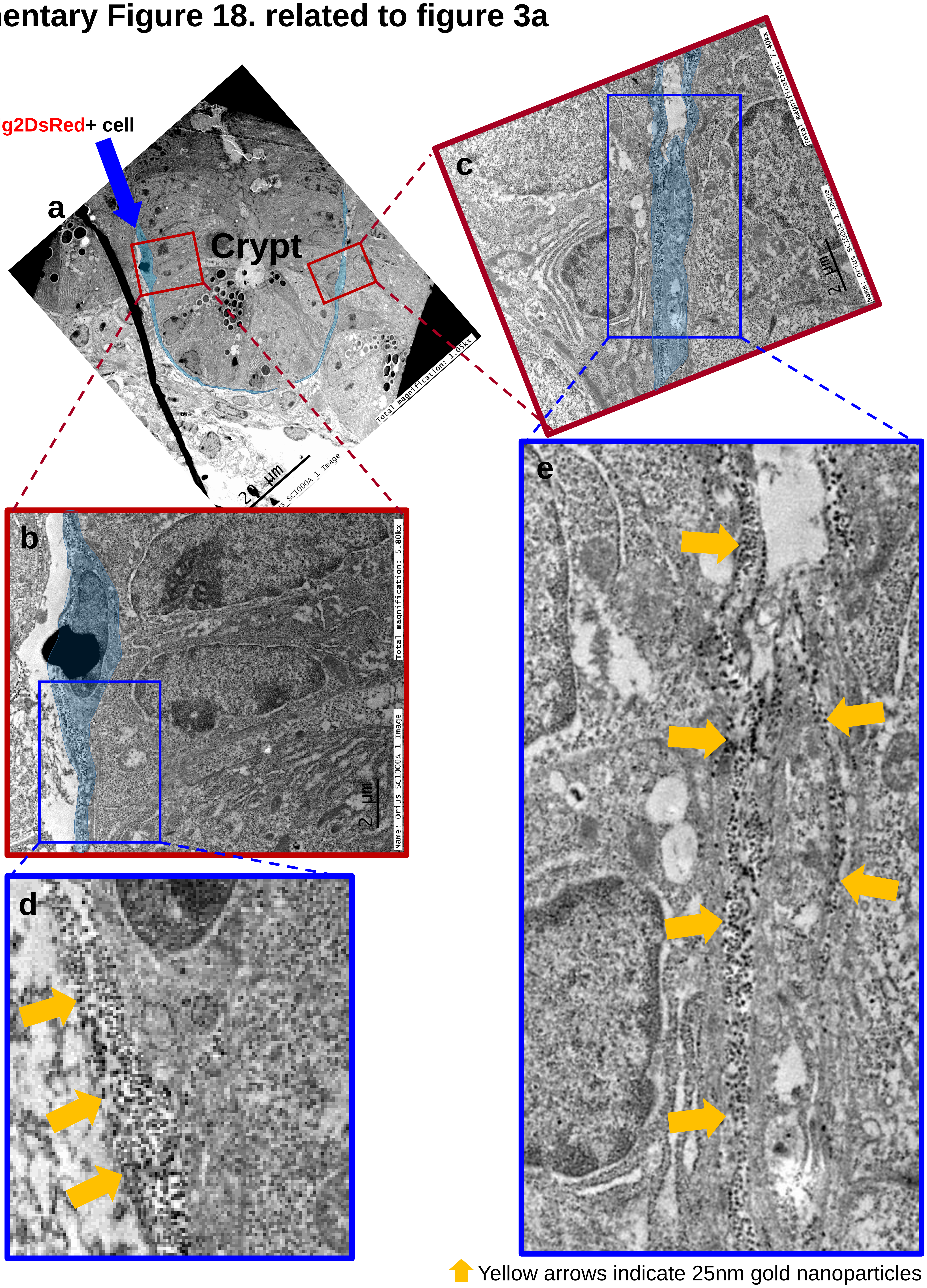

Supplementary Figure 18. Immunogold transmission electron microscopy (TEM) analysis of pericyte-like stromal cells surrounding the crypt (related to Figures 3a).
(a) TEM image of the crypt in *Ng2*$^{+/DsRed}$ ileum. Blue colored area indicates pericyte-like stromal cells (telocytes) labeled with 25 nm gold nanoparticles. Scale bar indicates 20 μm. (b, c) TEM images magnified from red boxes in (a). Scale bars indicate 2 μm. (d, e) TEM images magnified from blue boxes in (b) and (c). These magnified images show 25 nm gold nanoparticles labeled in pericyte-like stromal cells (telocytes). Yellow arrows indicate 25 nm gold nanoparticles staining DsRed expressing cells. n=2, Each n means biologically independent animals and experiments.

Supplementary Figure 19. related figure 3b

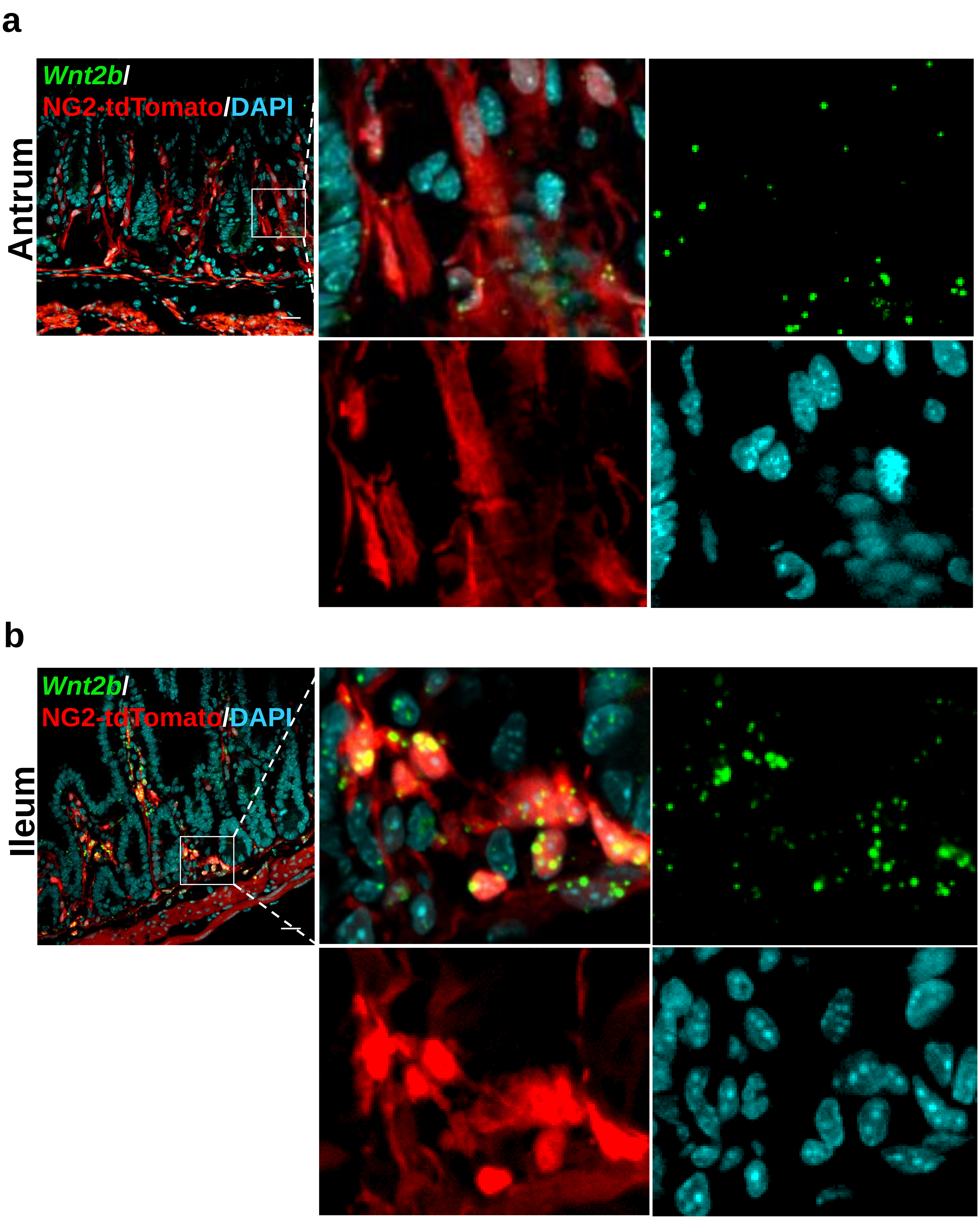

Supplementary Figure 19. Expression of *Wnt2b* in *Ng2-Cre* lineage traced stomach and intestinal stromal cells (related to Figures 3b).

(a, b) Single-molecule FISH of *Wnt2b* (green dot) transcripts in the antrum (a) and ileum (b) of *Ng2-Cre*;*Rosa26*$^{+/tdTomato}$ mouse. Left panels show low magnification, and right panels indicate magnified images of white square insets. Scale bars indicate 20 µm.

Supplementary Figure 20. related figure 3c

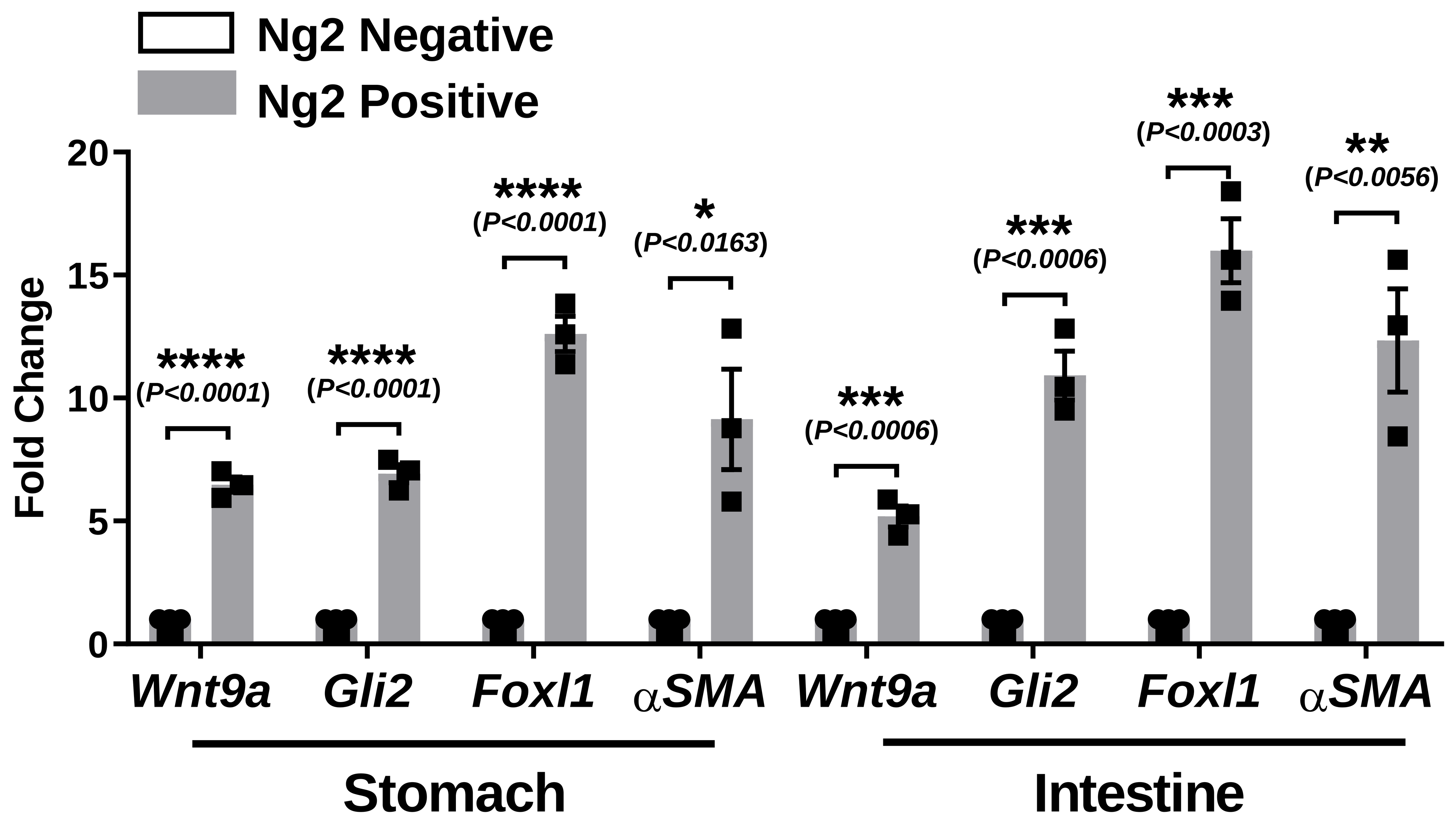

Supplementary Figure 20. Characterization of *Ng2-Cre* lineage traced cells using quantitative reverse transcript PCR (qRT-PCR) (related to Figure 3c).

qRT-PCR of *Wnt9a*, *Gli2*, *Foxl1* and *αSMA* in FACS sorted tdTomato positive and negative cells in *Ng2-Cre*;*Rosa26*$^{+/tdTomato}$. tdTomato positive cells express significantly higher levels of *Wnt9a*, *Gli2*, *Foxl1* and *αSMA* transcripts, compared to tdTomato negative populations. n=3 per group, *P< 0.05, **P< 0.01, ***P< 0.001, ****P< 0.0001. P-values were determined using nonparametric unpaired Student's t test. Values are mean ± SEM. n=3, Each n means biologically independent animals and experiments.

Supplementary Figure 21. related figure 3b, 3g and 4b

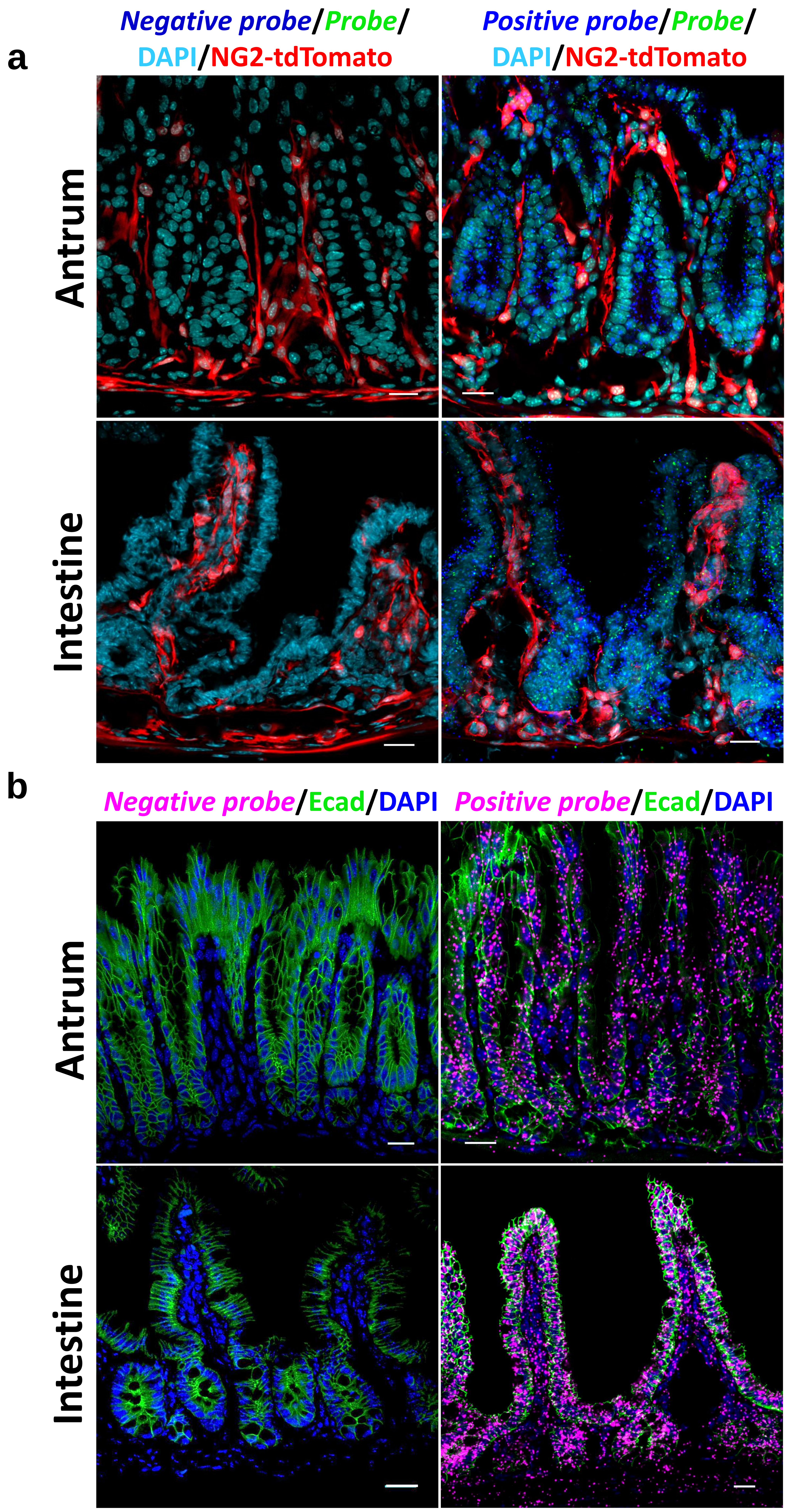

Supplementary Figure 21. smFISH images of negative and positive probes in gastrointestinal tissues (related to Figure 3b, 3g and 4b).

(a) Images of negative (left) control with a probe targeting *DapB* and of positive (right) control with probes targeting *Polr2a* (green) and *Ppib* (blue) in the antrum (upper panel) and ileum (lower panel) related to Fig. 3b and 4b. (b) Images of negative control with a probe targeting *DapB* (left) and a positive control probe targeting *Ubc* (right; magenta) co-stained with E-cadherin (green) in gastrointestinal tissues used for Fig. 3g. Scale bars indicate 20 μm.

Supplementary Figure 22. related figure 3

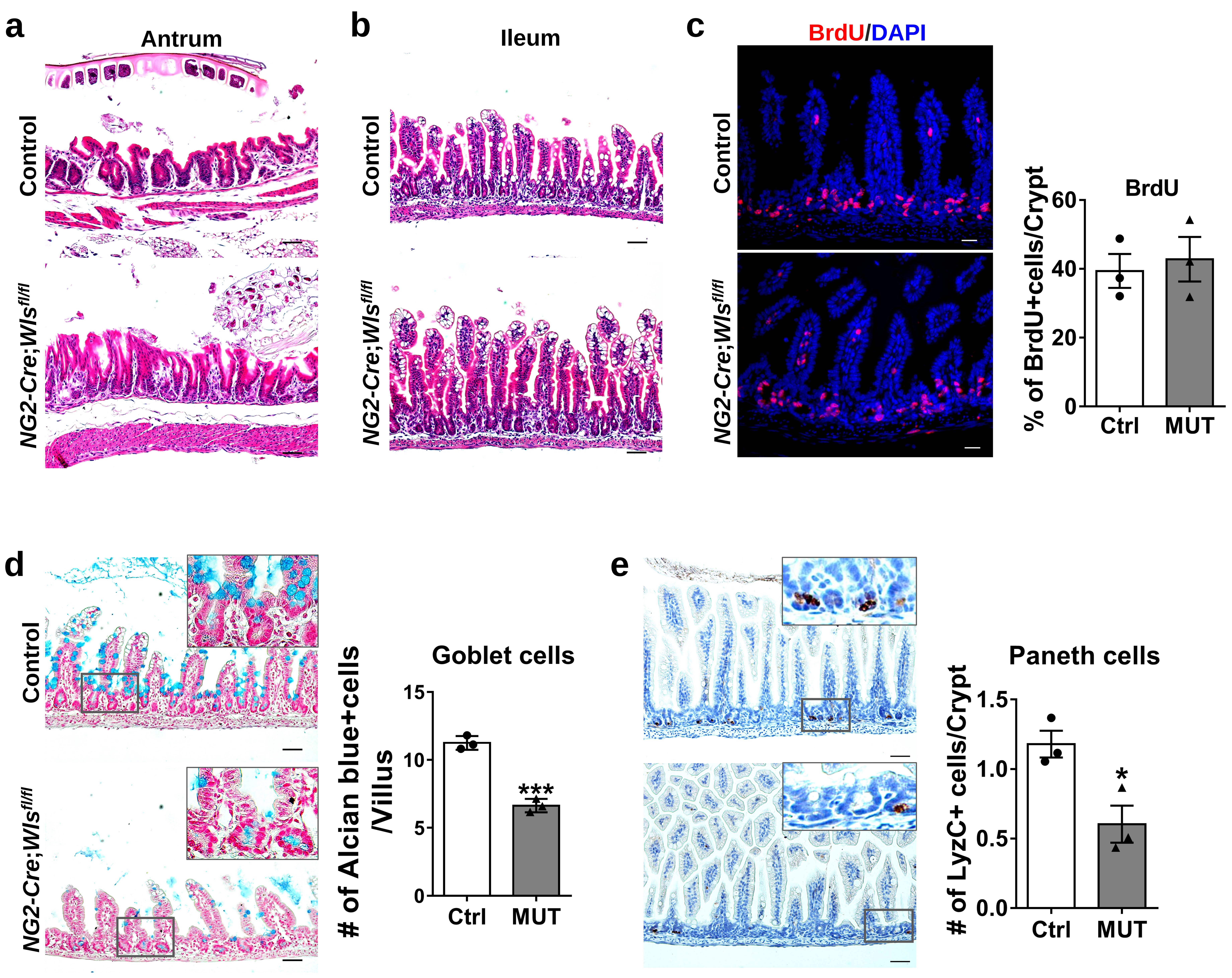

Supplementary Figure 22. Gastrointestinal differentiation and proliferation upon pericyte-like stromal cell-specific inhibition of Wnt secretion (related to figure 3).

(a, b) H&E staining shows gastrointestinal morphology in *Ng2-Cre*;*Wls*$^{fl/fl}$ mice and their littermate controls (P17). Scale bars indicate 50 µm. (c-e) BrdU labeling in the intestine show no obvious defects in epithelial proliferation (c), while Alcian blue staining (d) and immunohistochemistry (IHC) of Lysozyme C (e) indicate a significantly decreased number of goblet cell and Paneth cells, respectively, in *Ng2-Cre*;*Wls*$^{fl/fl}$ mice compared to the controls (P14). Scale bars indicate 50 µm. n=3 per group, $^{**}P < 0.01$, $^{***}P < 0.001$. *P*-values were determined using nonparametric unpaired Student's t test. Values are mean ± SEM. n=3, Each n means biologically independent animals and experiments.

Supplementary Figure 23. related figure 4

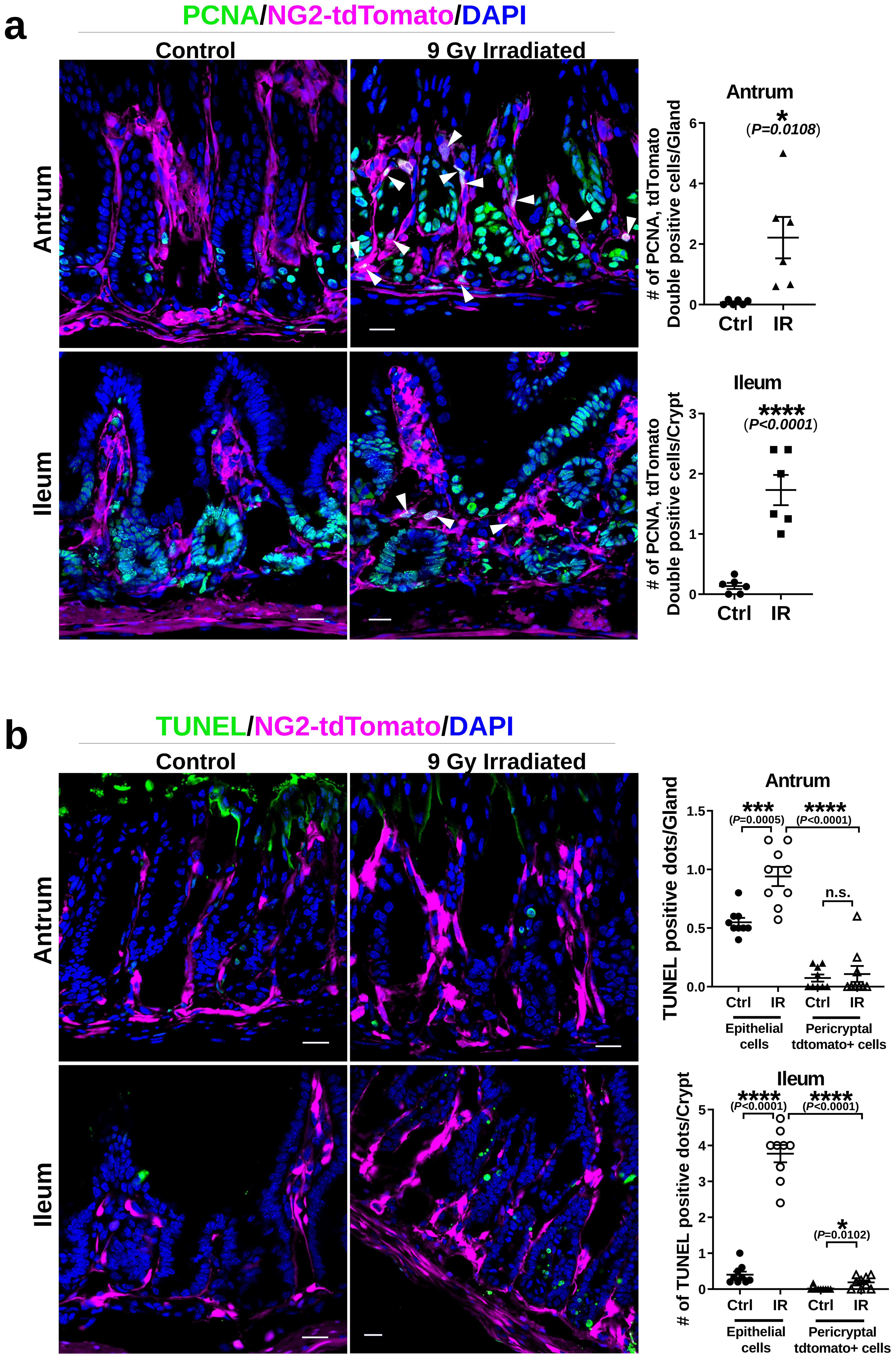

Supplementary Figure 23. Proliferation and survival of pericyte-like stromal cells after irradiation (related to Figure 4).

(a) IF of PCNA in *Ng2-Cre*;*Rosa26*$^{+/tdTomato}$ tissues after 48 hours of 9 Gy irradiation in the antrum (upper panel) and ileum (lower panel). Left and right panels indicate non-irradiated P32 control samples and P32 tissues treated with 9 Gy irradiation, respectively. Arrow head indicates PCNA (green) and tdTomato (red) doubly positive cells, and their number is significantly increased after 48 hours of irradiation. (b) TUNEL assay showing a significantly increased number of TUNEL positive dots in epithelial cells after irradiation, compared to the controls. Analysis of intestinal tdTomato+ pericryptal cells in *Ng2-Cre*;*Rosa26*$^{+/tdTomato}$ mice showed a mildly increased number of TUNEL positive dots, while antral tdTomato+ pericryptal cells showed no significant difference. Significantly increased numbers of TUNEL positive dots in epithelial cells are found after irradiation when compared to those in pericryptal cells. Scale indicates 20 μm. n=3 per group, $^*P< 0.05$, $^{***}P< 0.001$, $^{****}P< 0.0001$. *P*-values were determined using nonparametric unpaired Student's t test. Values are mean ± SEM. Each n means biologically independent animals and experiments.

Supplementary Figure 24 related figure 4

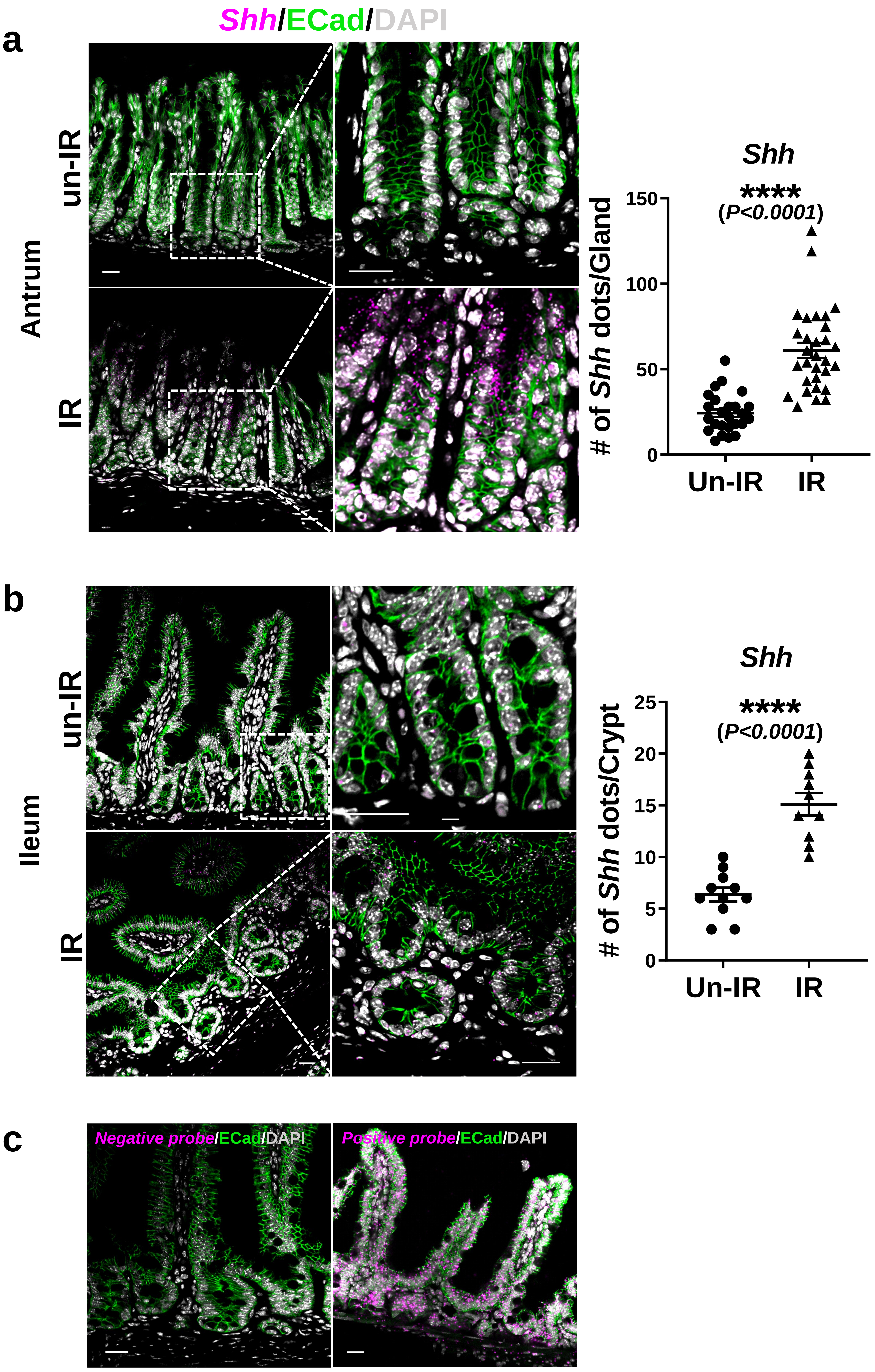

Supplementary Figure 24. Increased *Shh* transcripts after irradiation in gastrointestinal epithelial cells (related to Figure 4).

(a, b) smFISH of *Shh* transcripts in P32 control and irradiated tissues after 48 hours of 9 Gy irradiation in the antrum (a) and ileum (b). Magnified images of white square boxed areas are shown in right panels. E-cadherin (green) is co-stained with smFISH of *Shh* probe (magenta). Scale indicates 20 μm. n=3 per group, ****P< 0.0001. P-values were determined using nonparametric unpaired Student's t test. Values are mean ± SEM. (c) smFISH images of a negative (left) probe targeting *DapB* and of a positive (right) probe targeting *Ppib* (magenta) imaged with the same laser intensity with *Shh* probe. Scale indicates 20 μm. Each n means biologically independent animals and experiments.

Supplementary Figure 25. related to figure 5

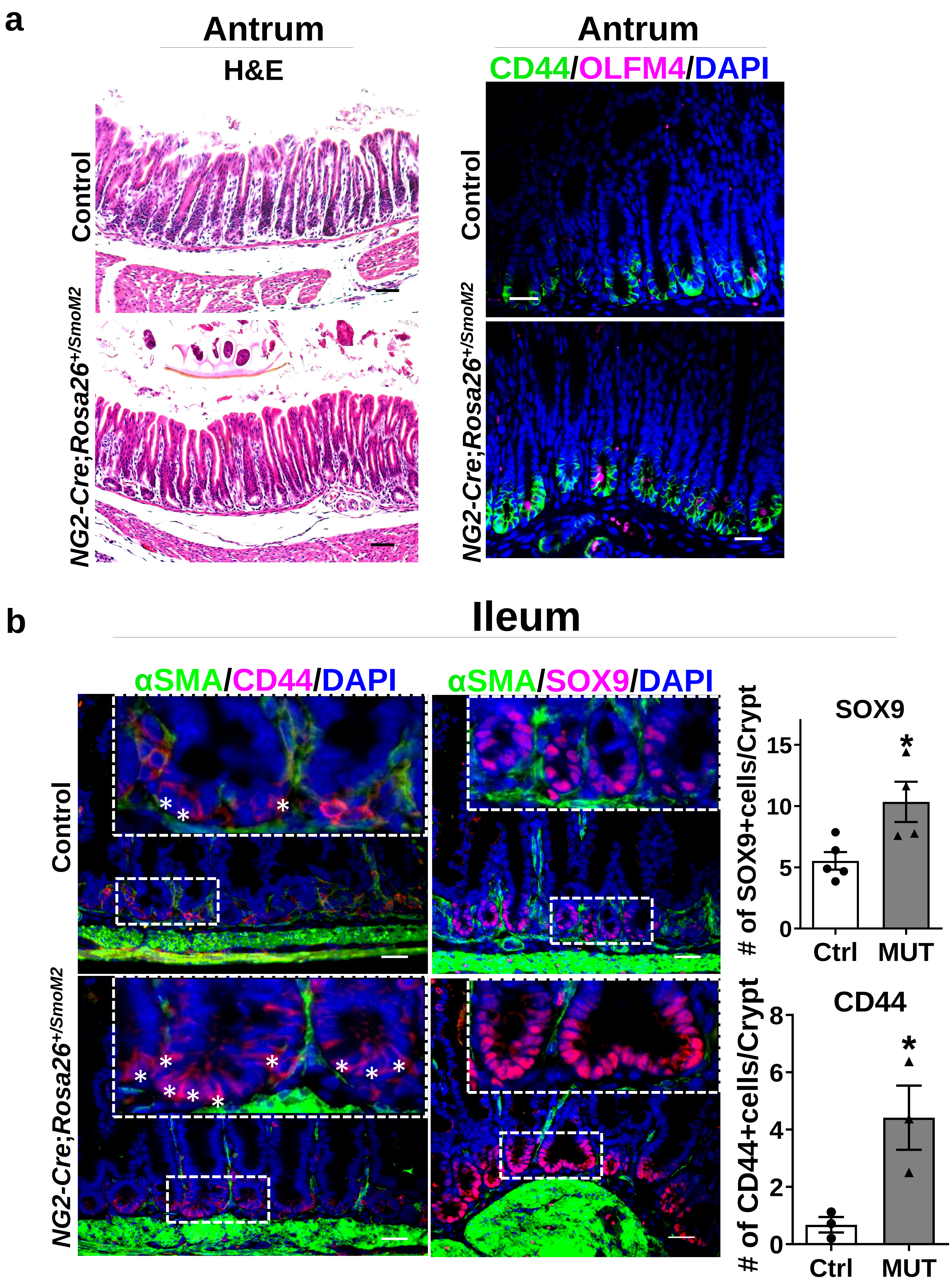

Supplementary Figure 25. Increased numbers of gastrointestinal progenitors upon Hh activation in pericyte-like stromal cells (related to Figure 5).
(a) H&E staining (Left panel) and IF (Right panel) of CD44 (green) and OLFM4 (magenta) in the antrum. Scales indicate 50 μm (Left panel) and 20 μm (Right panel). (b) IF of CD44 and SOX9 in ileum shows that the numbers of CD44+ and SOX9+ progenitors are significantly increased in *Ng2-Cre*;*Rosa26*$^{+/SmoM2}$ mice compared to the controls (n=3 per group), *$P<0.05$, **$P<0.01$. Scales indicate 20 μm. *P*-values were determined using nonparametric unpaired Student's t test. Values are mean ± SEM. Each n means biologically independent animals and experiments.

Supplementary Figure 26. related to figure 5e and 5f

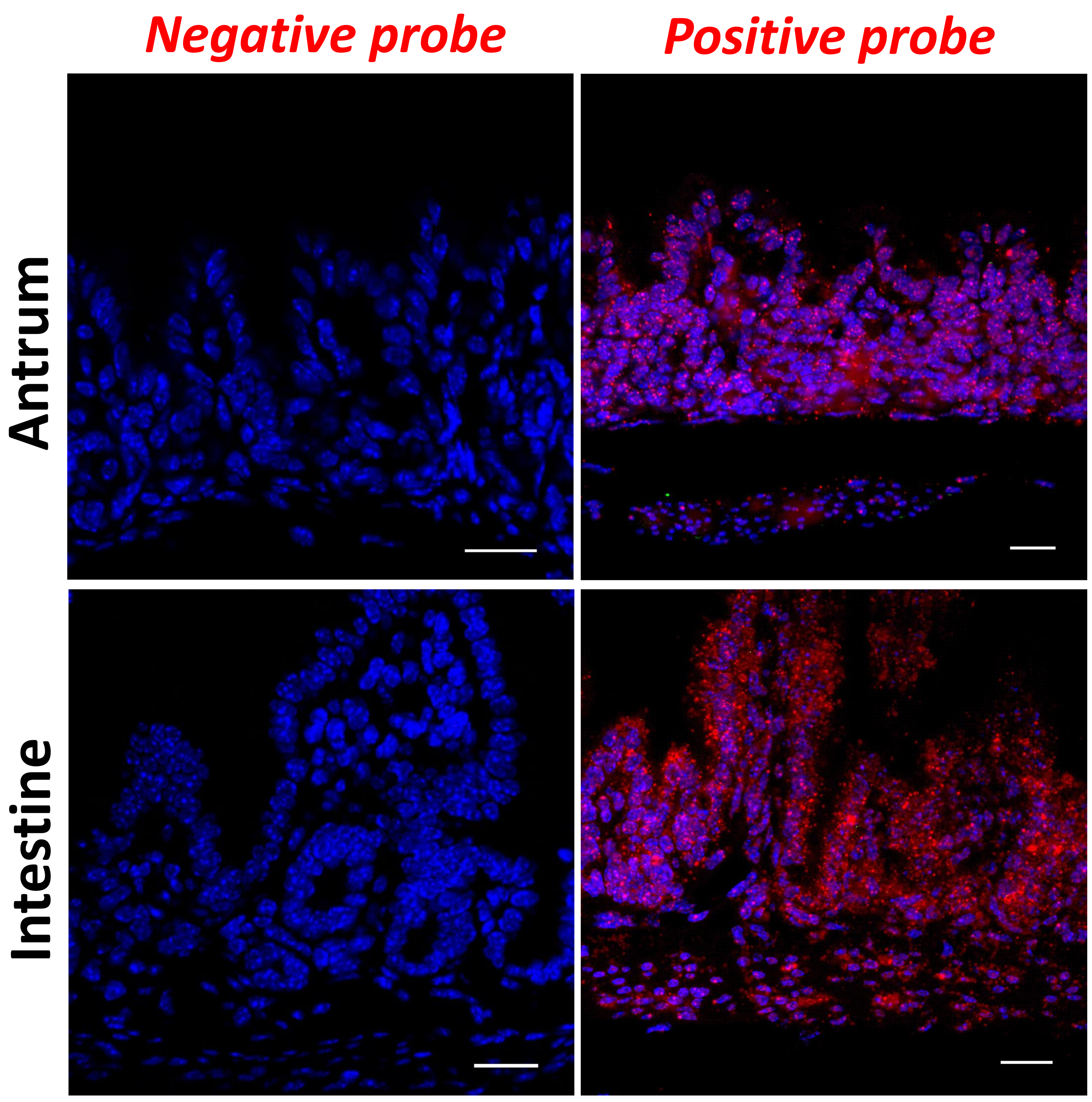

Supplementary Figure 26. smFISH images of negative and positive probes in gastrointestinal tissues (related to Figure 5e and 5f).

(a) Images of negative control (left) with a probe targeting *DapB* and of positive control (right) with a probe labeling *Polr2a* (red) in the antrum (upper panel) and ileum (lower panel) in gastrointestinal tissues related Figure 5e and 5f. Scale bars indicate 20 µm.

Supplementary Figure 27. related to figure 6

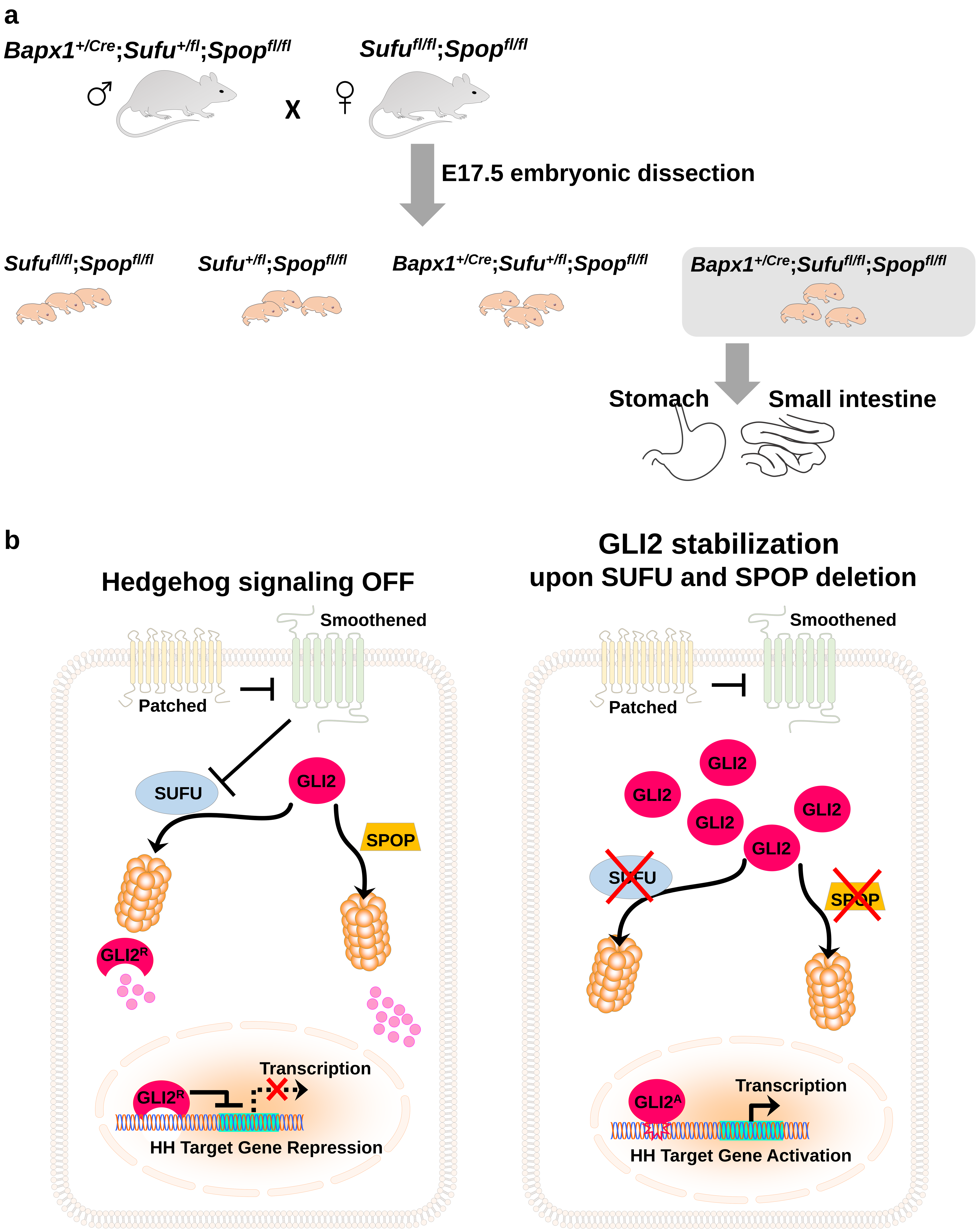

Supplementary Figure 27. Schematic diagram for Gli2 ChIP-seq (related to Figure 6).
(a) Mouse cross for Gli2 ChIP-seq. (b) Strategy for stabilization of GLI2 transcription factor for ChIP-seq. Left panel shows cytoplasmic retention and fast degradation of GLI2 by SUFU and SPOP. Right panel shows stabilized, nuclear GLI2 achieved by deleting *Sufu* and *Spop*, which are known to facilitate inactivation and degradation of GLI2.

Supplementary Figure 28. related to figure 6

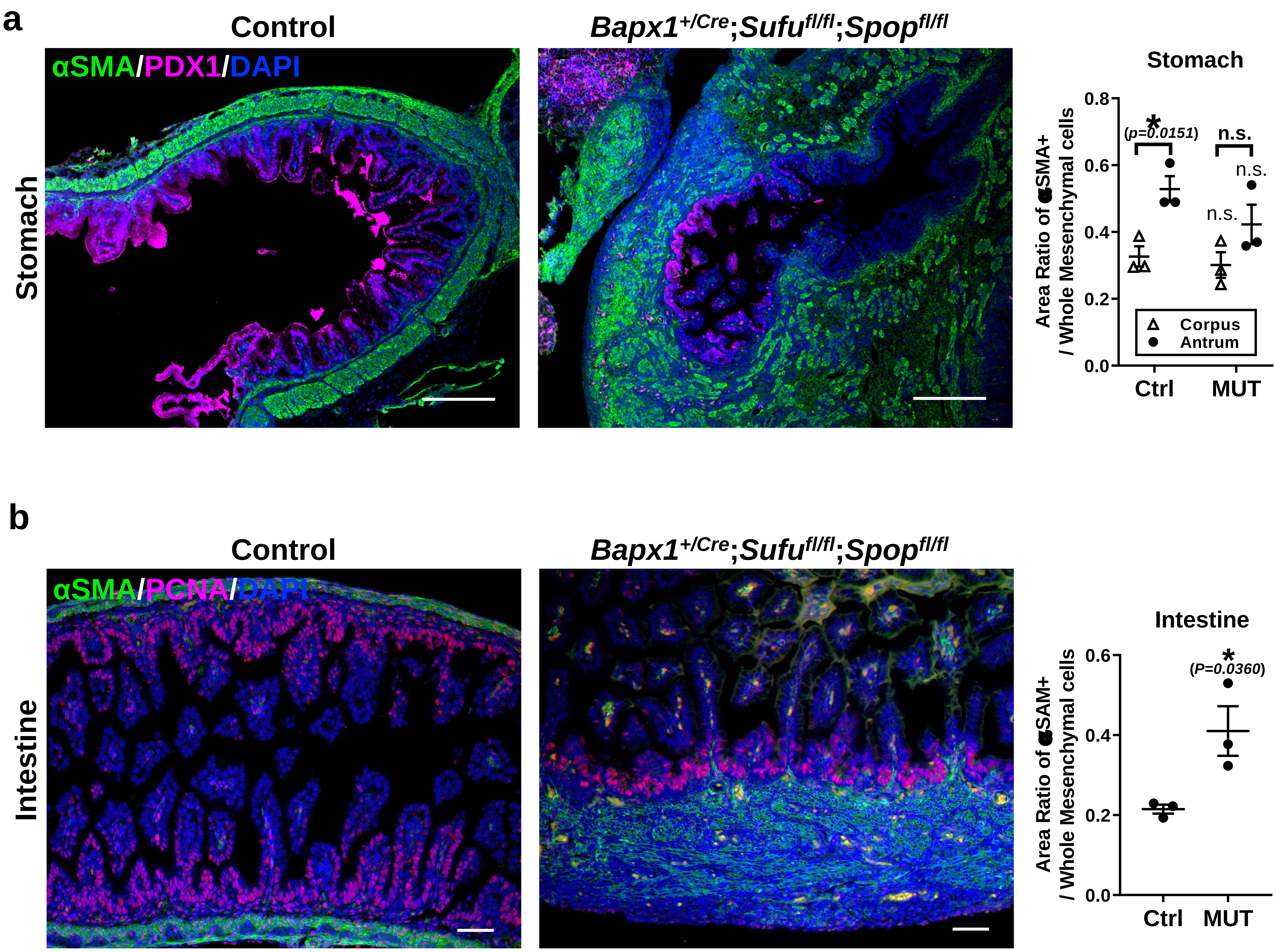

Supplementary Figure 28. Percentage of αSMA expressing cells in the mesenchyme of mutant pups used for GLI2 ChIP-seq (related to Figure 6).
(a) IF of αSMA (green) and PDX1 (red) in the *Bapx1*$^{+/Cre}$;*Sufu*$^{fl/fl}$;*Spop*$^{fl/fl}$ stomach. The percentages of smooth muscle cells among whole mesenchymal cells are approximately 30% and 40-50% in the corpus and the antrum, respectively. These ratios were not significantly different between mutant and control. Scale bars indicate 100 μm. (b) IF of αSMA (green) and PCNA (magenta) in the *Bapx1*$^{+/Cre}$;*Sufu*$^{fl/fl}$;*Spop*$^{fl/fl}$ intestine. The percentage of smooth muscle cells in whole mesenchymal cells is significantly increased in mutant compared to control. (n=3 per group), $^{*}P< 0.05$. *P*-values were determined using nonparametric unpaired Student’s t test. Scales indicate 20 μm. Values are mean ± SEM. Each n means biologically independent animals and experiments.

Supplementary Figure 29. related to figure 6g, 6h and 6i

a

Stomach_*Bapx1*$^{+/Cre}$;*Rosa26*$^{+/tdTomato}$

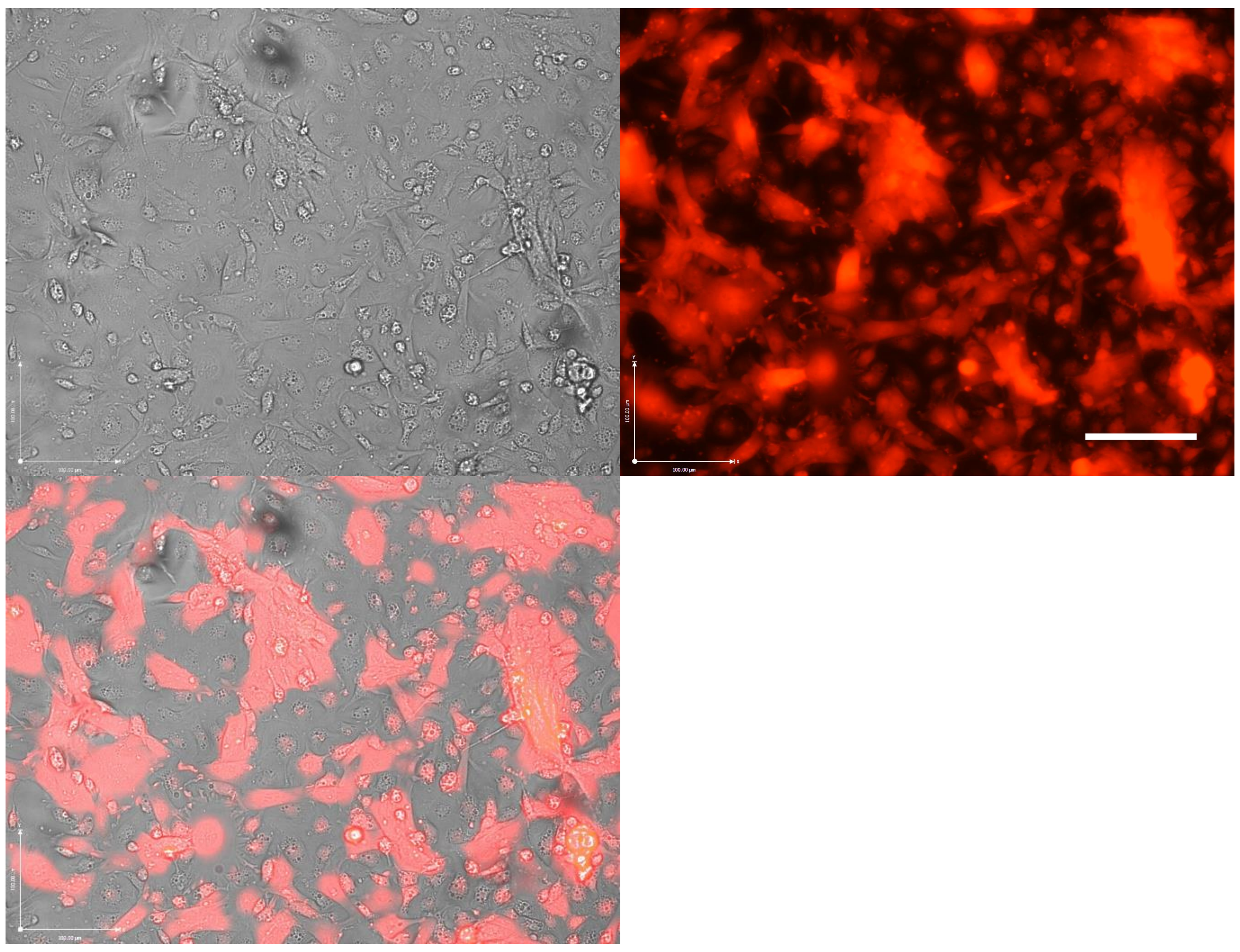

b

Intestine_*Bapx1*$^{+/Cre}$;*Rosa26*$^{+/tdTomato}$

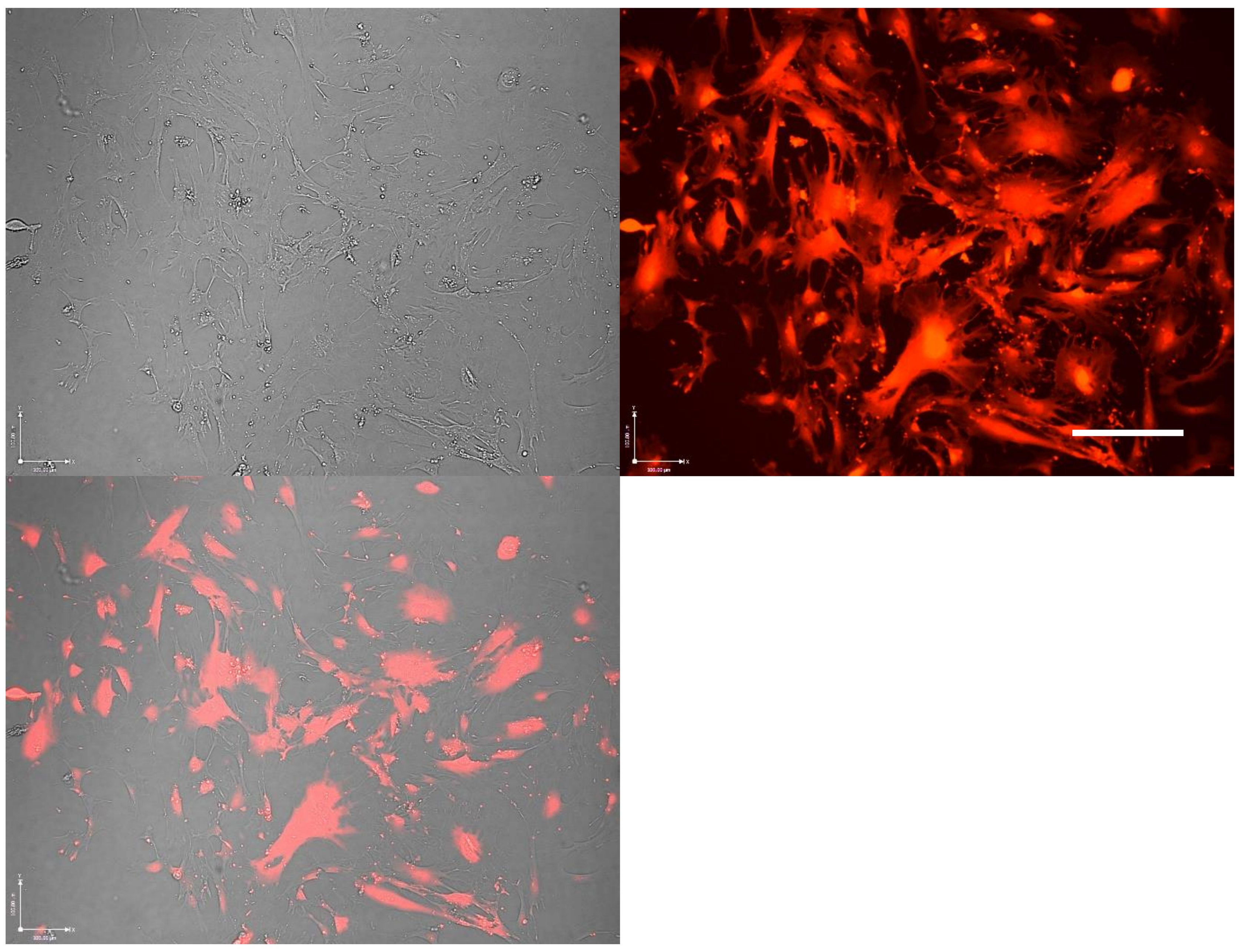

c Genomic GLI2 peak regions of *Wnt2b*

>mm9_dna range=chr3:104796734-104797168 5'pad=0 3'pad=0 strand=+
repeatMasking=none
CTACAGCCAGCTGGGATTACCCAACTCCCAGAAAGGAAGAGTGAGGAATG
TGGACTGATG**TGGGTGCT**GGGGAGTTGTTGGTGGGGGCTGGGAGGCATGA
GGAGGCCCGGGGCAGGGAGCAGAGCTTTCCCATGGTGAGAATAAGGCCCA
CATAGAGAATGAGGGAGGGAGAGTTGCCAAGATCTGAGTTATAGCAGAAG
GAAGCTGAGCCCTGAATGGGAGCCTTGTGTGTGGCCCTGTGCTGCTGACT
CACCAGGCTGAGGAGAGCGAAAACATGCTAGGGATGAAGAAGGGGCCTGT
TTATGCCCTG**CCACCA**CCTCCAAACCGCCCTG**CCACCCCA**GATTGCTTTG
GTCAGGGGCTTCCTGAGGACACCAGCTGCCCCGAGGTACAAAGAGTCCTG
GAAGGCTGTGATTTAGGTGGGACAGGCAGGGGAGA

Light Blue : Primer region
Yellow : Gli2 binding region

d Genomic GLI2 peak regions of *Wnt9a*

>mm9_dna range=chr11:59149833-59150235 5'pad=0 3'pad=0 strand=+
repeatMasking=none
AAGGGTTATAGCCCTTTCCTGGCAGCCCAGGATAATGTCCTGGGGTCTCA
GCACAGTAAGGCATGGGCTGGAAAGGGGCAGTGGTCCTATCCAAAGCTTT
TCTGGCCTGCTCAGGGATGGGAGTGGGGGAAGGGTCCAGCAAGTTGCCAA
GGAAACAAGAGCAAACAAACACTTCCTCCCCTCACAGGCCTGCTGGCCTG
CTGGGGGGCCTGTGGCAATGACCTCATCTG**CCCACCCA**ACCCTACTCCCA
AGGCCAGGCTGAAGGTGGGGGGTCCTGGGCTGTTTGTGTTGGGCCCAGAT
CCATGGAAACGGCTGGGGCTGGGGGTGCAGGGCTGTTCCTGCTGCAGTCT
GTCAGTAGGTGGCAGGAATATGGAGAGGGAAGGAAAGTGAGAGGGCAGAG
AGA

Light Blue : Primer region
Yellow : Gli2 binding region

Supplementary Figure 29. Luciferase assays of genomic regions (related to Figure 6g, 6h and 6i). (a, b) Stomach (a) and intestinal (b) mesenchymal cells were isolated from *Bapx1*$^{+/Cre}$;*Rosa26*$^{+/tdTomato}$ mice and cultured for 5 days. Scale bar indicates 50 µm. (c, d) Genomic regions of *Wnt2b* (435bp; Chr3 104796734 - 104797168) and *Wnt9a* (255bp; Chr11 59336431 - 59336685) luciferase reporter fragments are shown. Primer and GLI2 binding regions are highlighted in light blue and yellow, respectively.

Supplementary Figure 30. related to figure 6g

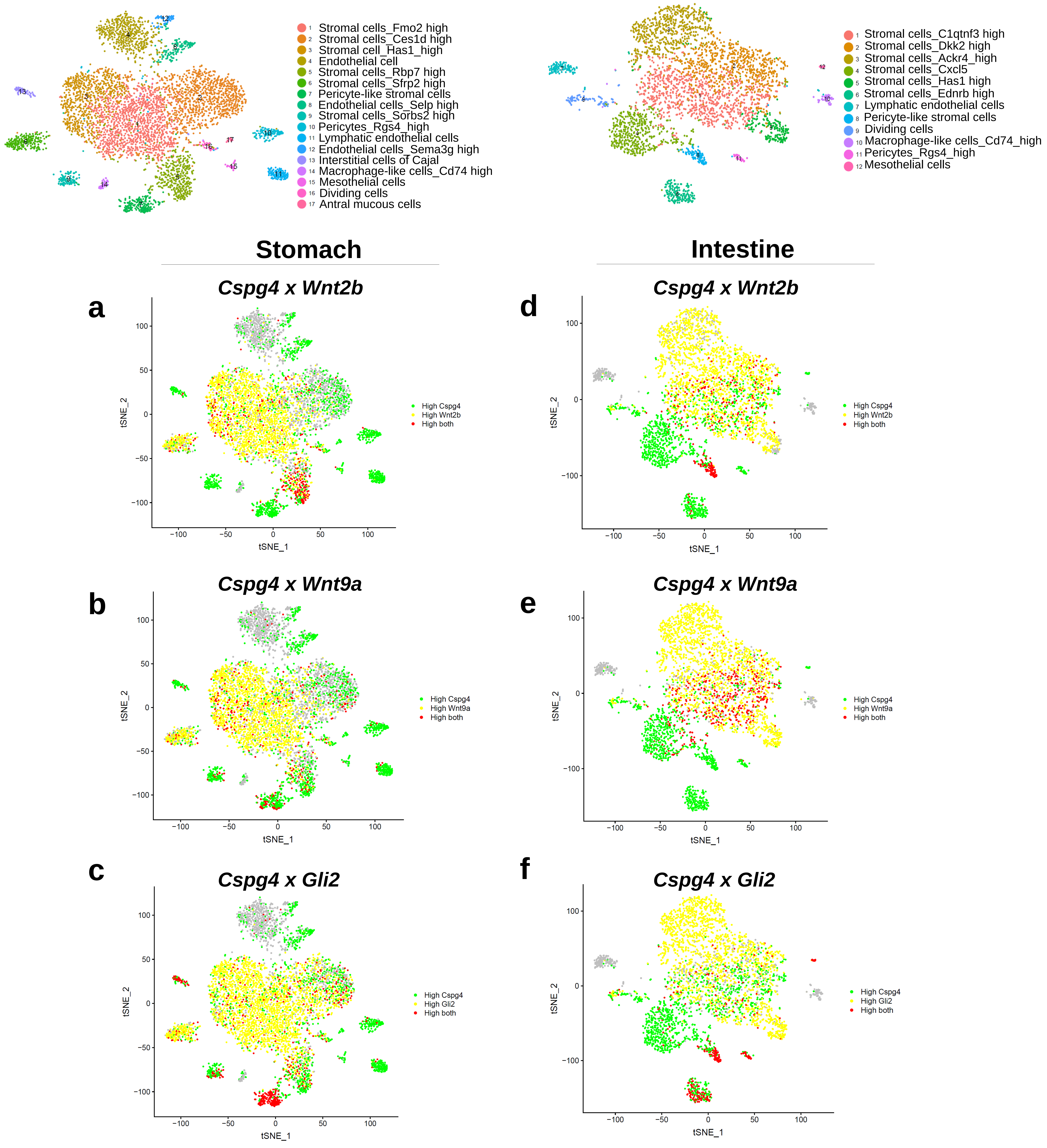

Supplementary Figure 30. co-featured plots (FP) of *Cspg4, Gli2, Wnt2b* and *Wnt9a* in the stomach and intestine (related to Figure 6g).

(a, d) co-FP of *Cspg4* and *Wnt2b* of the stomach (a) and intestine (d). (b, e) co-FP of *Cspg4* and *Wnt9a* of the stomach (b) and intestine (e). (c, f) co-FP of *Cspg4* and *Gli2* of the stomach (c) and intestine (f). Green dots indicate *Cspg4*, while yellow dots indicate *Wnt2b* (a, c), *Wnt9a* (b, e) and *Gli2* (c, f). Red dots mean cells expressing both genes. Values were determined based on cluster means.

Supplementary Figure 31. related to figure 7

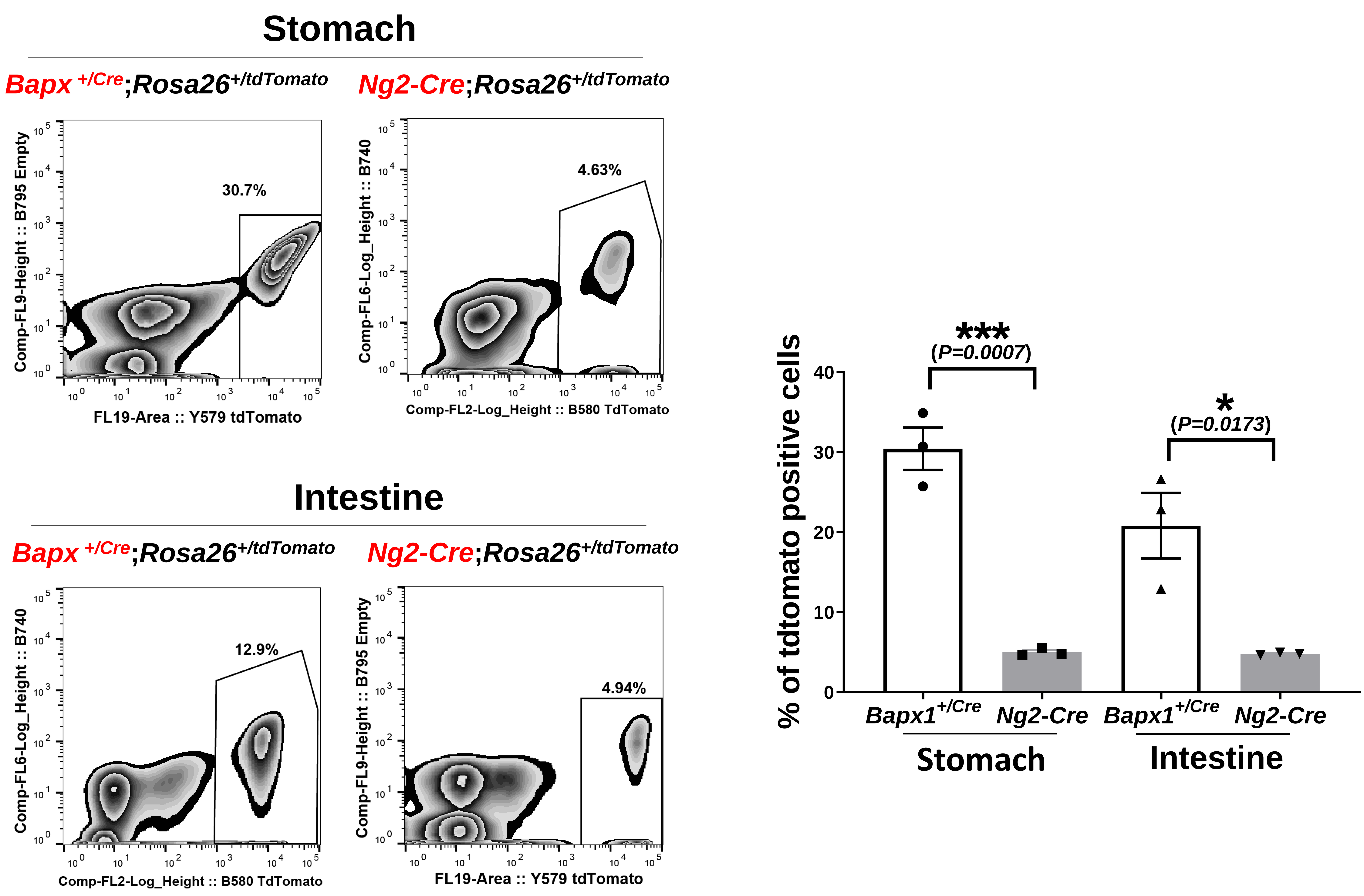

Supplementary Figure 31. ***Bapx1*** $^{+/Cre}$ **labels more mesenchymal cell populations than** ***Ng2-Cre*** **(related to Figure 7).**

(a) When compared to whole mesenchymal cells after EDTA shaking, the percentage of tdTomato positive cells is 25-35% in *Bapx1* $^{+/Cre}$;*Rosa26* $^{+/tdTomato}$ mice, while it is only 4-6% in *Ng2-Cre*;*Rosa26* $^{+/tdTomato}$ mice, n=3 per group. *P< 0.05, ***P< 0.001. Values are mean ± SEM. Each n means biologically independent animals and experiments.

Supplementary Figure 32. related to figure 7

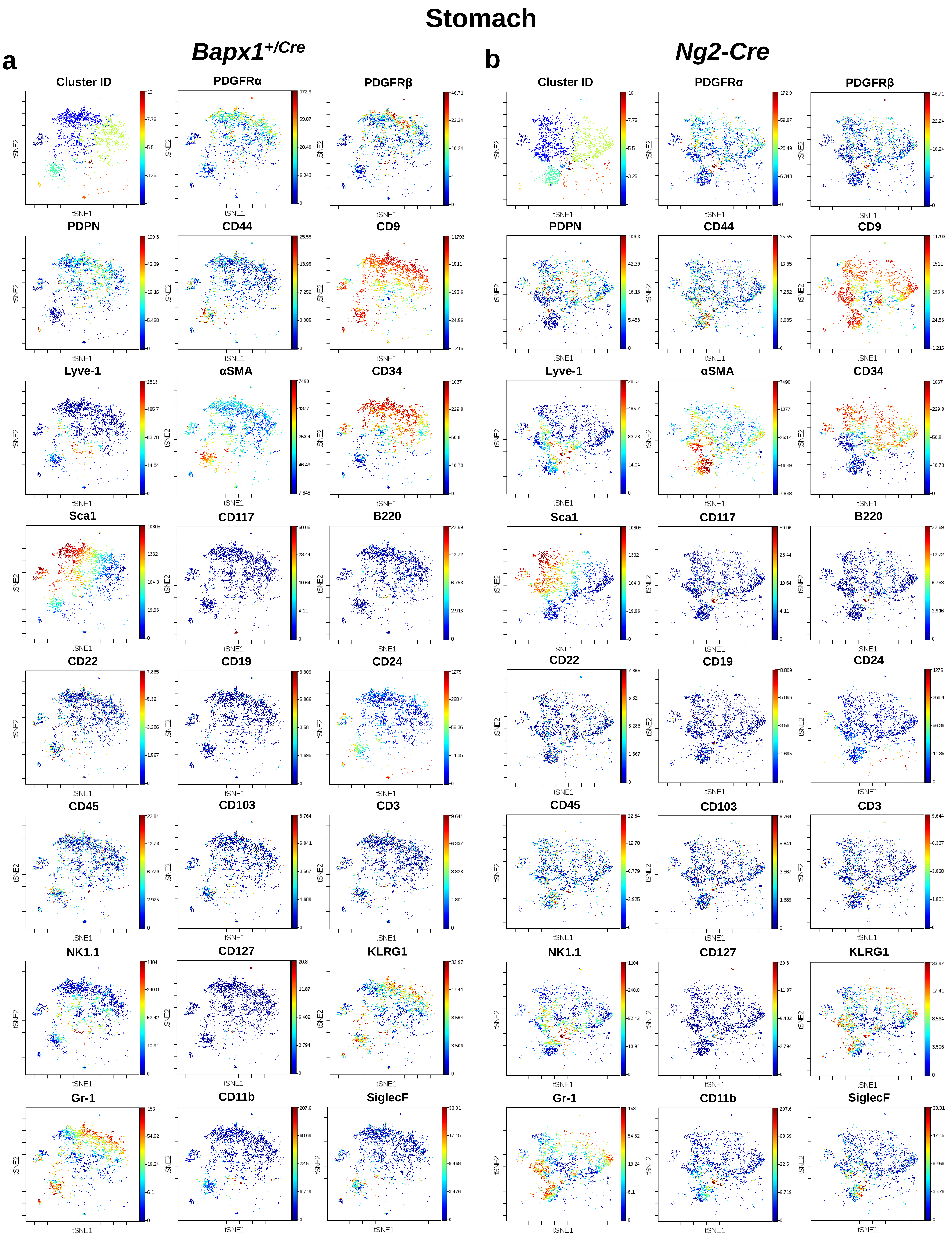

Supplementary Figure 32. continued. related to figure 7

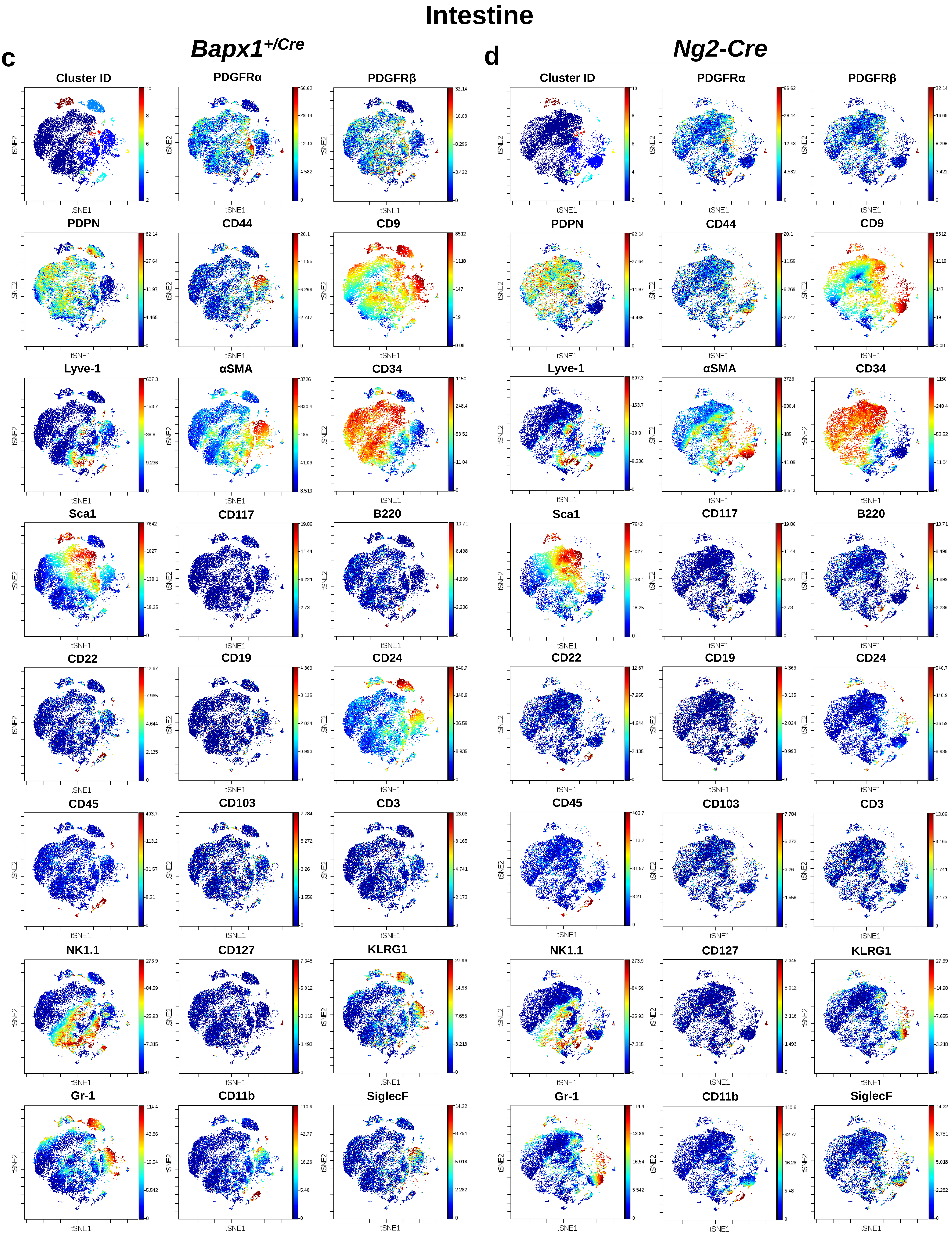

Supplementary Figure 32. continued. related to figure 7

Supplementary Fig. 32. The expression pattern of all markers used for CyTOF analysis (related to Figure 7).

(a, b, c, d) Starting from the left, t-SNE plots showing FlowSOM cluster ID followed by the expression pattern of gut stromal cell markers (i.e. Pdgfrα, Pdgfrβ, PDPN, CD44, CD9, Lyve-1, αSMA), mesenchymal/heamatopoietic stem cell markers (i.e. CD34, Sca1, CD117), and immune cell markers: B cell markers (i.e. B220, CD22, CD19, CD24), T cell markers (CD45, CD103, CD3, NK1.1, CD127, KLRG1), neutrophil marker (Gr-1), CD11b, eosinophil marker (SiglecF). Panels (a) and (b) correspond to CyTOF data of stomach tissue using *Bapx1*$^{+/Cre}$ and *Ng2-Cre*, respectively. Stomach stromal cells from two mice were pulled for stomach CyTOF analysis. Panels (c) and (d) correspond to CyTOF data of intestinal tissue using *Bapx1*$^{+/Cre}$ and *Ng2-Cre*, respectively. Intestinal stromal cells from two mice were pulled for intestinal CyTOF analysis. t-SNE plots associated with CD31 and Ly6c are described in the following supplementary figure 32.

Supplementary Figure 33. related to figure 7

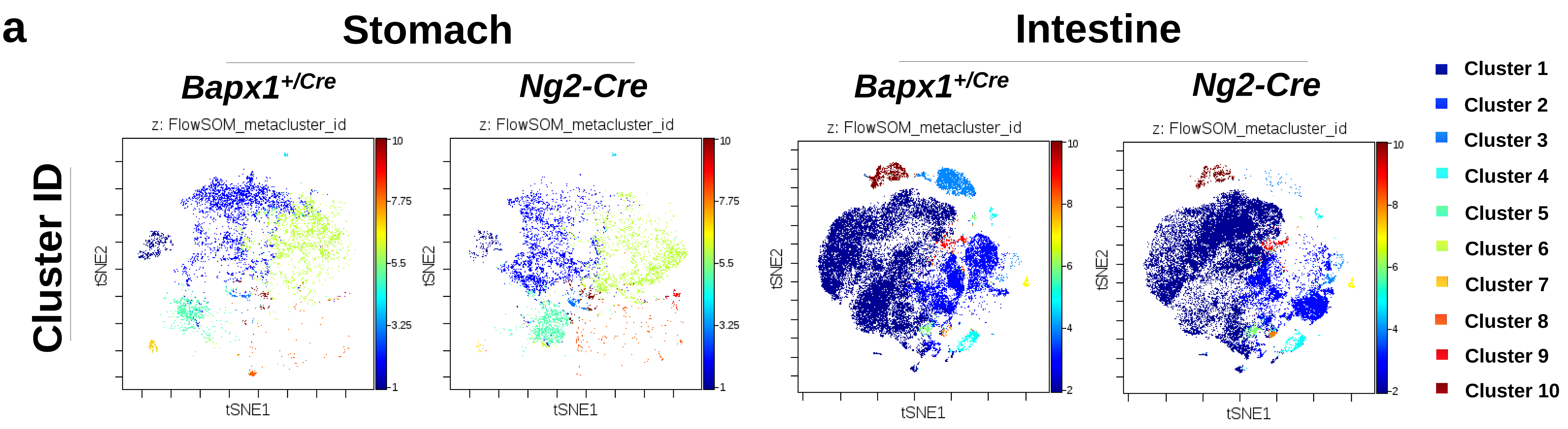

Stomach (Cell Number BreakDown)

	Bapx1+/Cre	*Ng2-Cre*
Cluster_1	332	146
Cluster_2	3155	2745
Cluster_3	91	196
Cluster_4	29	25
Cluster_5	749	1087
Cluster_6	2341	2327
Cluster_7	70	13
Cluster_8	130	179
Cluster_9	12	113
Cluster_10	91	169
Total	7000	7000

Intestine (Cell Number BreakDown)

	Bapx1+/Cre	*Ng2-Cre*
Cluster_1	280	394
Cluster_2	21314	22931
Cluster_3	7119	7572
Cluster_4	2167	358
Cluster_5	672	535
Cluster_6	348	374
Cluster_7	183	111
Cluster_8	99	300
Cluster_9	342	381
Cluster_10	809	377
Total	33333	33333

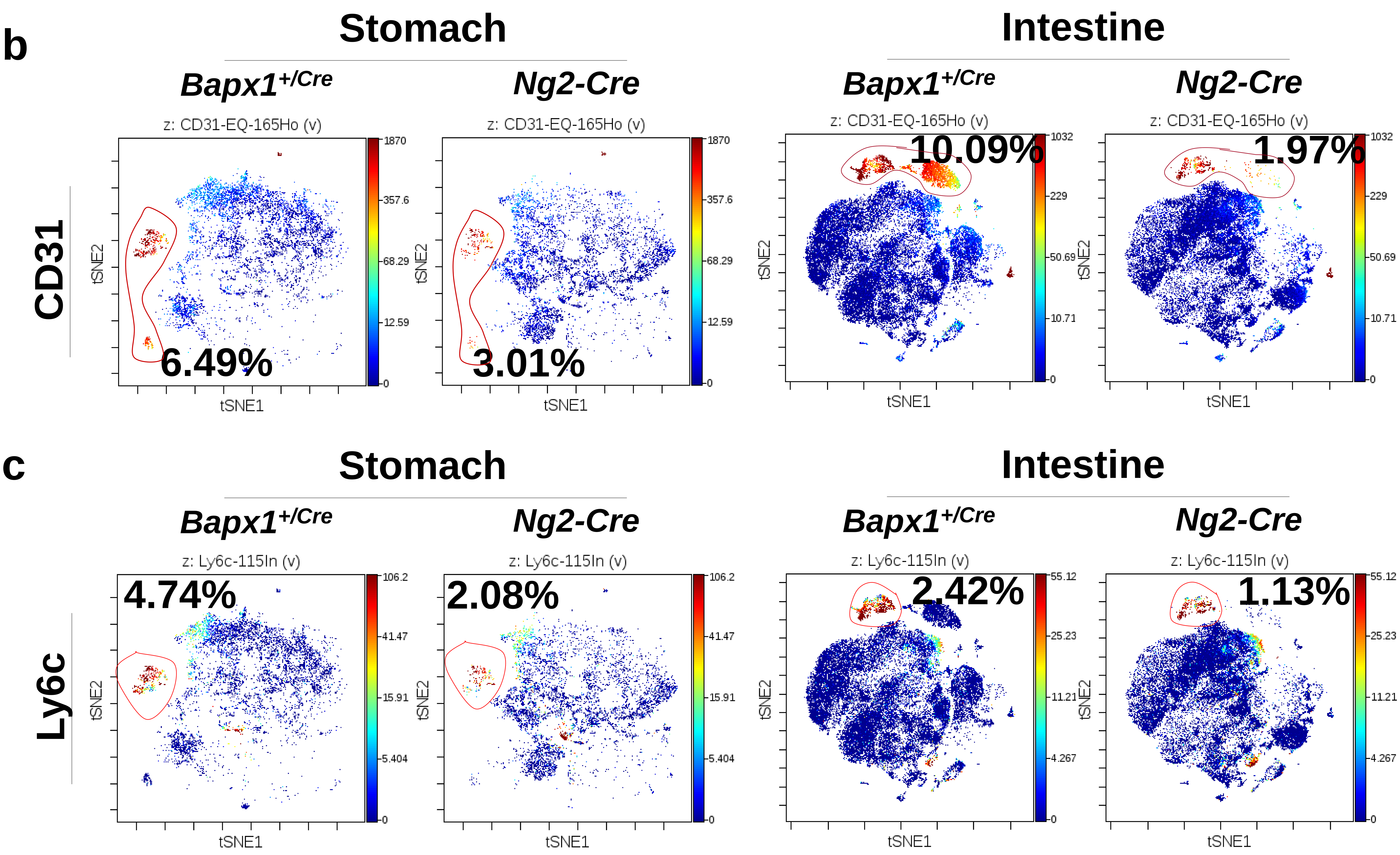

Supplementary Fig. 33. Major difference of CD31 expressing clusters between *Bapx1+/Cre* and *Ng2-Cre* labeled cells (related to Figure 7).

(a) t-SNE plots identify 10 different clusters from the CyTOF panel. Three mice were used for analysis, and intestinal cell numbers were downsized according to stomach cell numbers. The cell numbers of each cluster are identified in the table. (b, c) The cell numbers of CD31 (b) and Ly6c (c) expressing clusters (red lined) are significantly reduced in *Ng2-Cre*;*Rosa26+/tdTomato* mice, compared to *Bapx1+/Cre*;*Rosa26+/tdTomato* mice. The percentages of red lined clusters are indicated on each t-SNE plot.

Supplementary Figure 34.

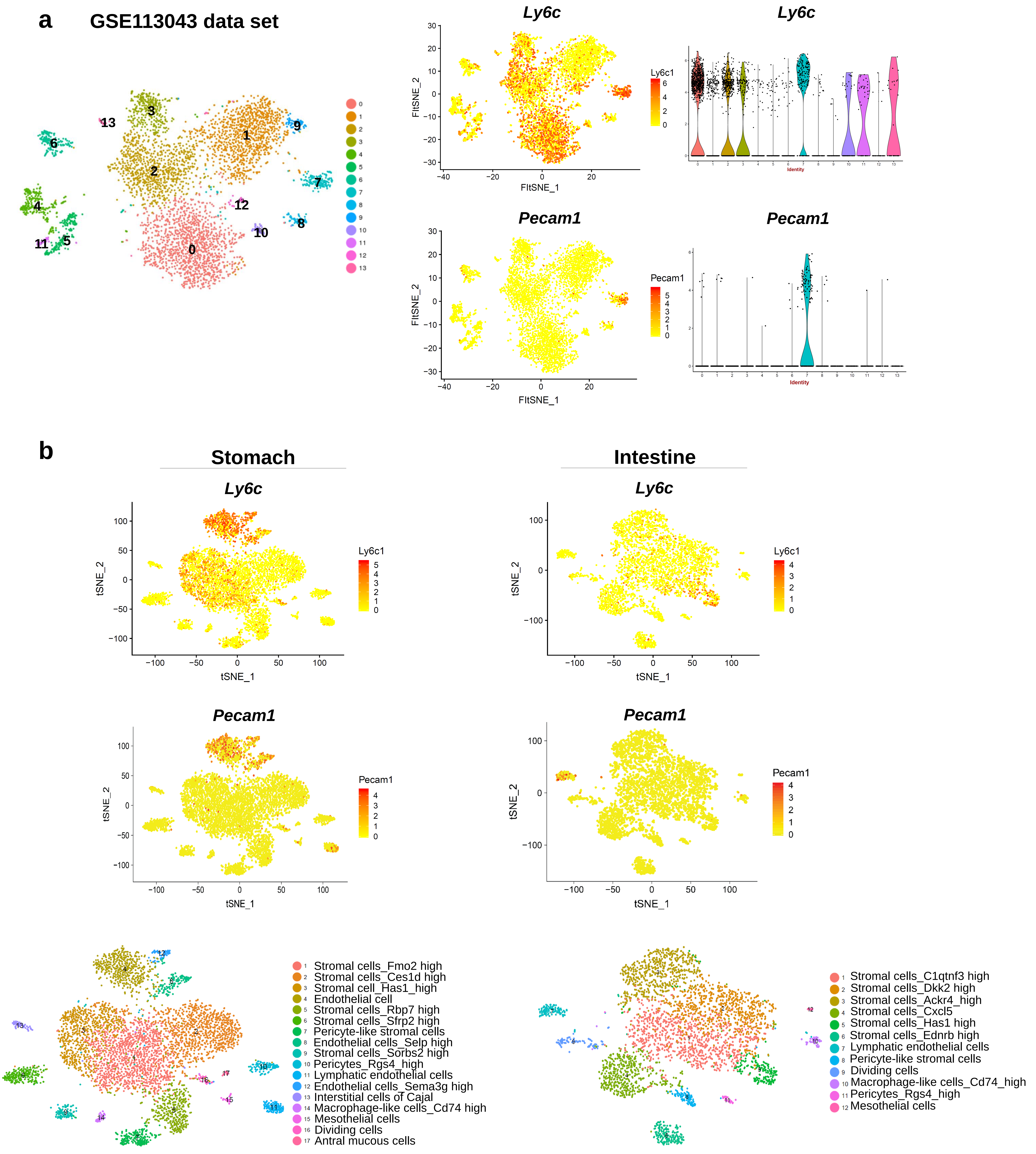

Supplementary Fig. 34. Feature plots and violin plots of *Ly6c* and *Pecam* (*Cd31*) analyzed from the published GSE113043 data set and our gastrointestinal scRNA-seq data.
(a) t-SNE plot identify 13 different clusters from GSE113043. The *Ly6c* is expressed throughout all stromal clusters from $Gli1^{CreERT2}$;$Rosa26^{+/tdTomato}$ mice. (b) Featured plots showing *Ly6c* and *Pecam* (*Cd31*) expressing clusters in our scRNA-seq data from $Bapx1^{+/Cre}$;$Rosa26^{+/tdTomato}$ mice.

Supplementary Figure 35.

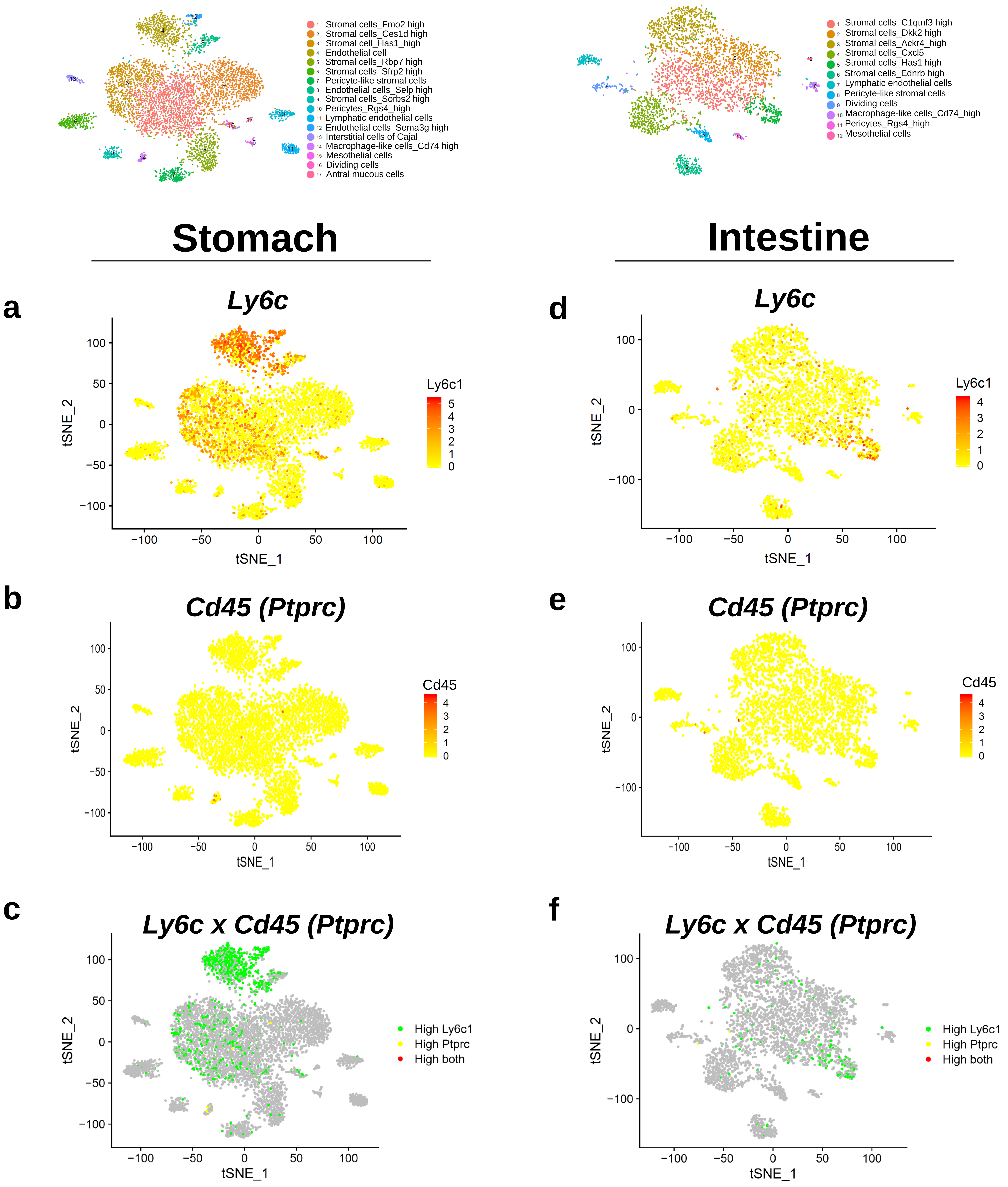

Supplementary Figure 35. Feature plots from gastrointestinal scRNA-seq data for *Ly6c* and *Cd45* (*Ptprc*).
(a, b) Feature plots from stomach scRNA-seq data (*Bapx1*$^{+/Cre}$;*Rosa26*$^{+/tdTomato}$ mice) showing *Ly6c* and *Cd45* (*Ptprc*) expressing cells. (c) Co-feature plot of *Ly6c* and *Cd45* (*Ptprc*) showing no overlap of *Ly6c* and *Cd45* expression. (d, e) Feature plots intestinal scRNA-seq data (*Bapx1*$^{+/Cre}$;*Rosa26*$^{+/tdTomato}$ mice) showing *Ly6c* and *Cd45* (*Ptprc*) expressing cells. (f) Co-feature plot of *Ly6c* and *Cd45* (*Ptprc*) showing no overlaps of *Ly6c* and *Cd45* expression.

Supplementary Figure 36.

CD45 & Ly6c double positive population

Stomach

Sample	Total Percentage	Event Count	Total Cell Count
Bapx1$^{+/Cre}$	**0%**	0	7000
Ng2-Cre	**0.01%**	1	7000

Intestine

Sample	Total Percentage	Event Count	Total Cell Count
Bapx1$^{+/Cre}$	**0.0%**	0	33333
Ng2-Cre	**0.0%**	0	33333

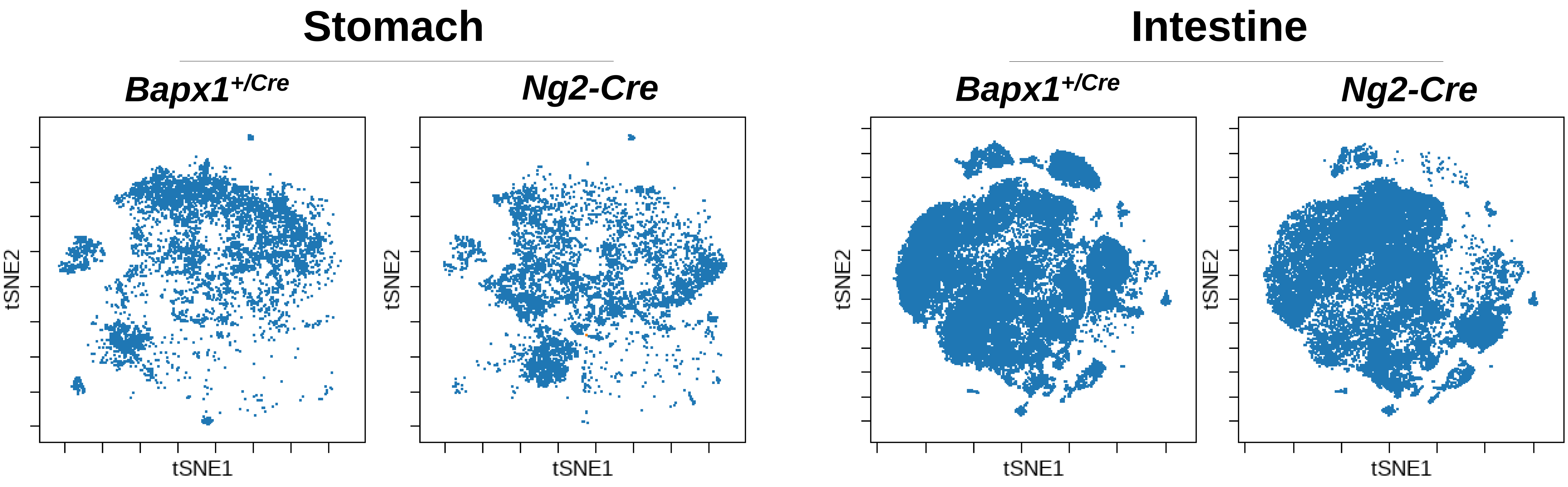

Supplementary Fig. 36. t-SNE plots from gastrointestinal CyTOF data for Ly6c and CD45. t-SNE plots from *Bapx1*$^{+/Cre}$;*Rosa26*$^{+/tdTomato}$ and *Ng2-Cre*;*Rosa26*$^{+/tdTomato}$ mice show no overlap of *Ly6c* and *Cd45* expression. *Ly6c* and *Cd45* doubly positive cells represent 0% in the stomach and intestinal tdTomato positive cells sorted from *Bapx1*$^{+/Cre}$;*Rosa26*$^{+/tdTomato}$ and *Ng2-Cre*;*Rosa26*$^{+/tdTomato}$ mice.

Supplementary Figure 37. related to figure 7

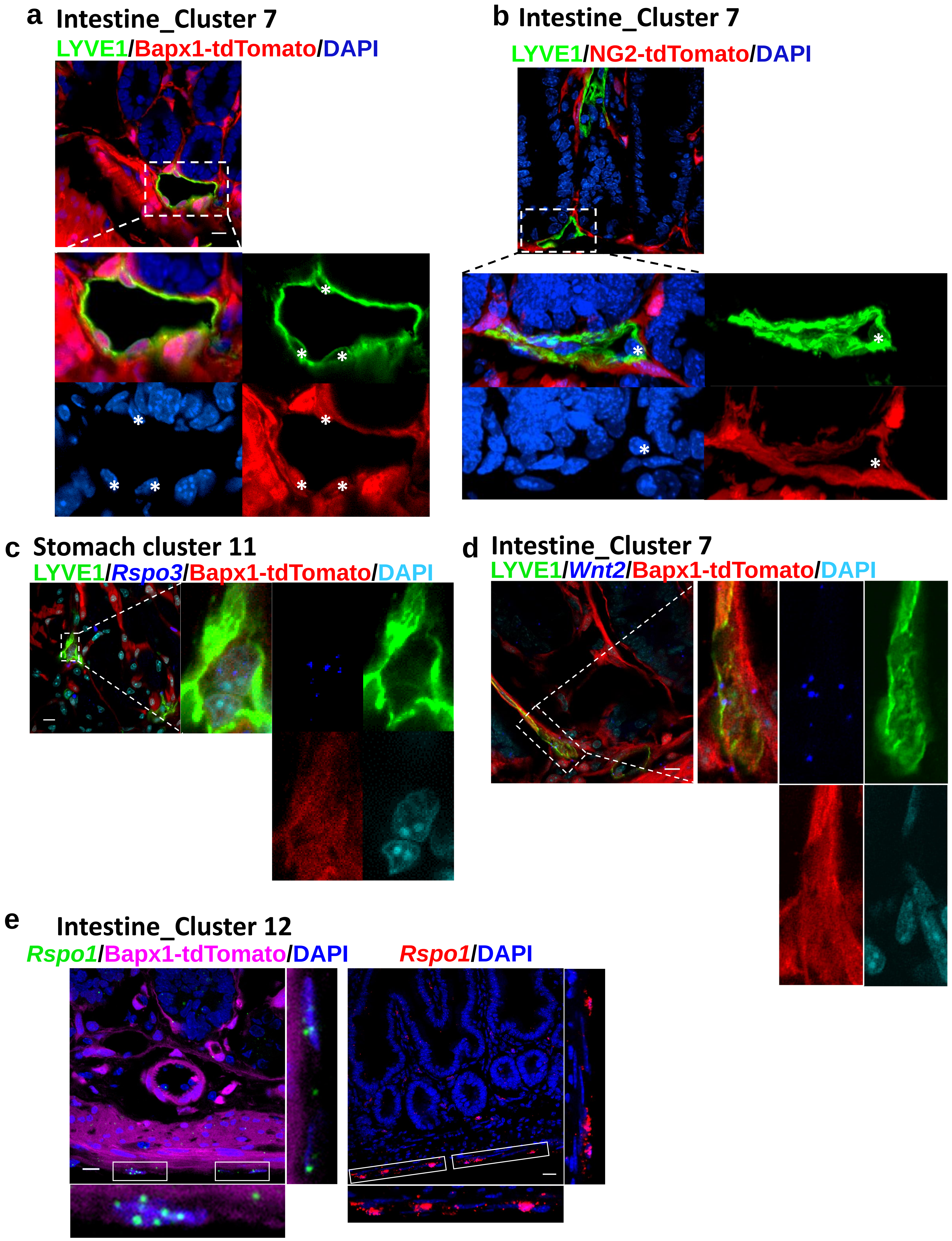

Supplementary Figure 37. Gut stromal cell populations labeled by *Bapx1*$^{+/Cre}$ but not by *NG2-Cre* lineage tracing (related to Figure 7).

(a, b) A *Bapx1*$^{+/Cre}$ allele (a) but not a *Ng2-Cre* allele (b) labels lymphatic vessels visualized by Lyve-1 staining (green). * indicates nucleus of lymphatic endothelial cells. (c, d) Lymphatic vessels express *Rspo3* (blue) in the stomach and *Wnt2* (blue) in the intestine. (e) Intestinal mesothelial cells labeled by the *Bapx1*$^{+/Cre}$ allele express *Rspo1* (left, green; right, red). All scale bars indicate 10 μm.

Supplementary Figure 38. related to figure 7

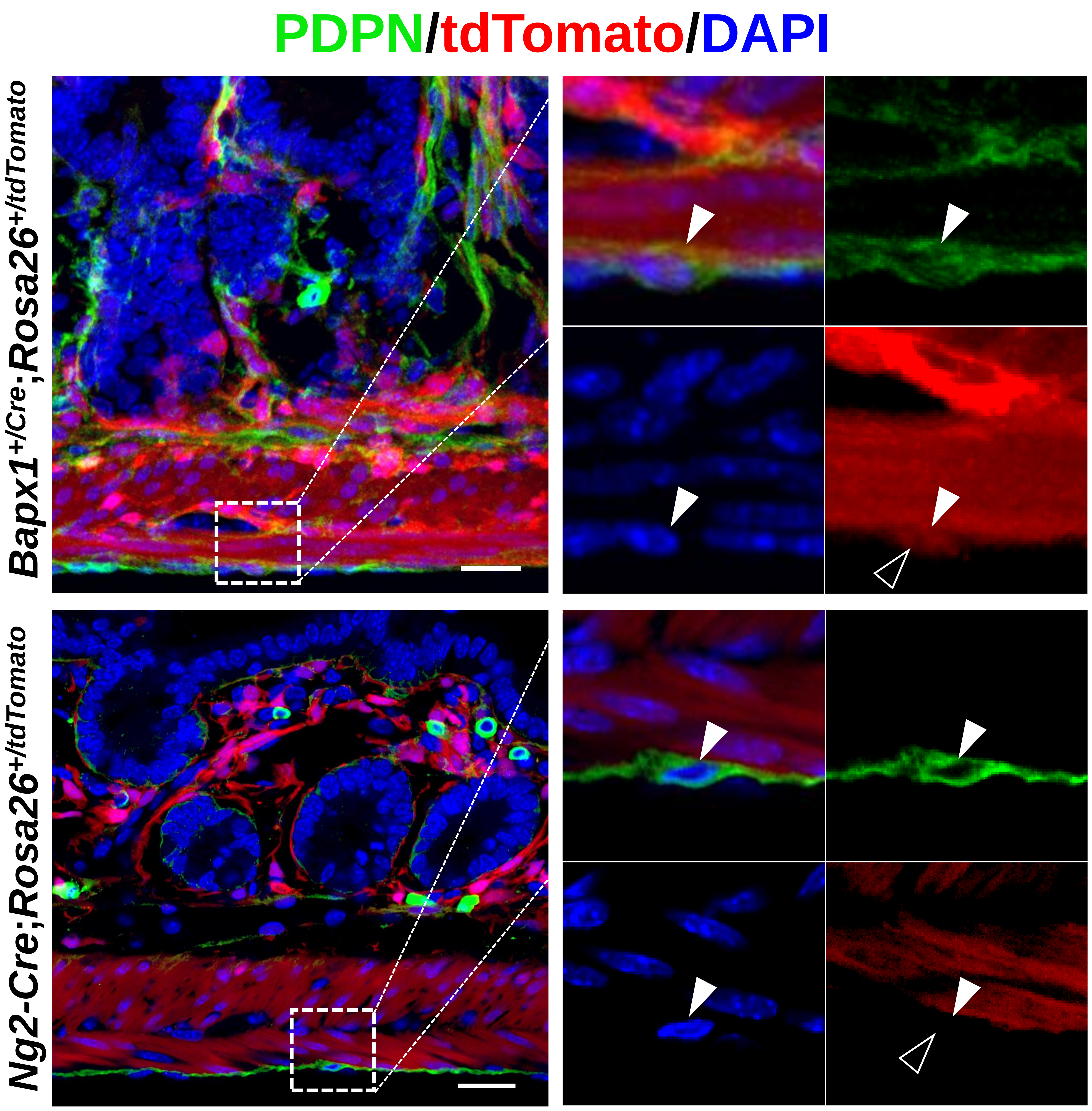

Supplementary Figure 38. Mesothelial cells are labeled by *Bapx1*$^{+/Cre}$ but not by *NG2-Cre* lineage tracing (related to Figure 7).

IF of PDPN (green), a mesothelial cell marker, in the *Bapx1*$^{+/Cre}$;*Rosa26*$^{+/tdTomato}$ (upper panel) and *Ng2-Cre*;*Rosa26*$^{+/tdTomato}$ (lower panel) mice. Filled arrow heads indicate mesothelial cells stained for PDPN, while empty arrow heads indicate tdTomato labeled mesothelial cells. *Ng2-Cre* lineage cells are not co-labeled with PDPN (right lower panel, empty arrow heads). Scale bar indicates 20 µm.

Supplementary Figure 39. related to figure 7

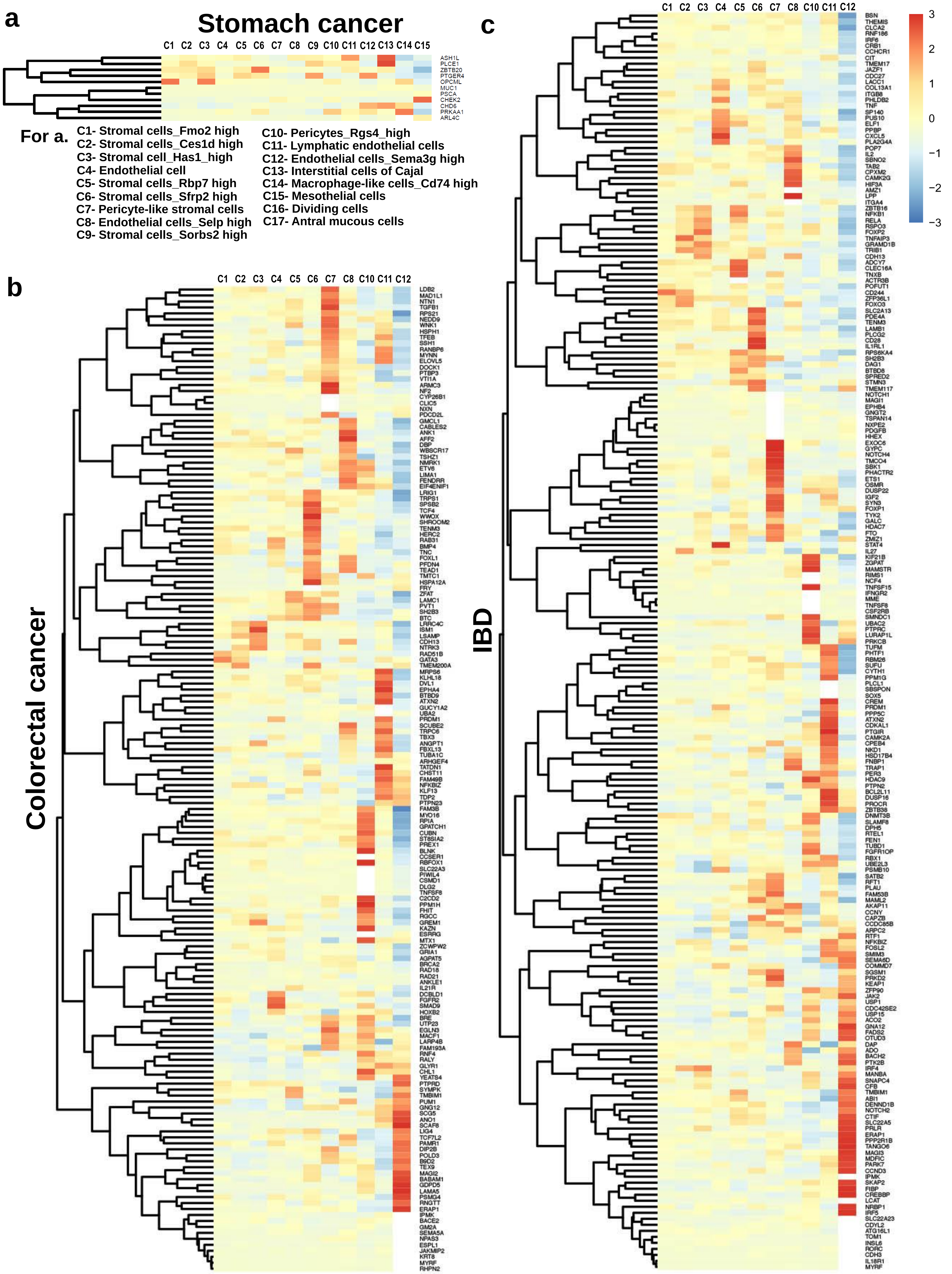

Supplementary Figure 39. continued. related to figure 7

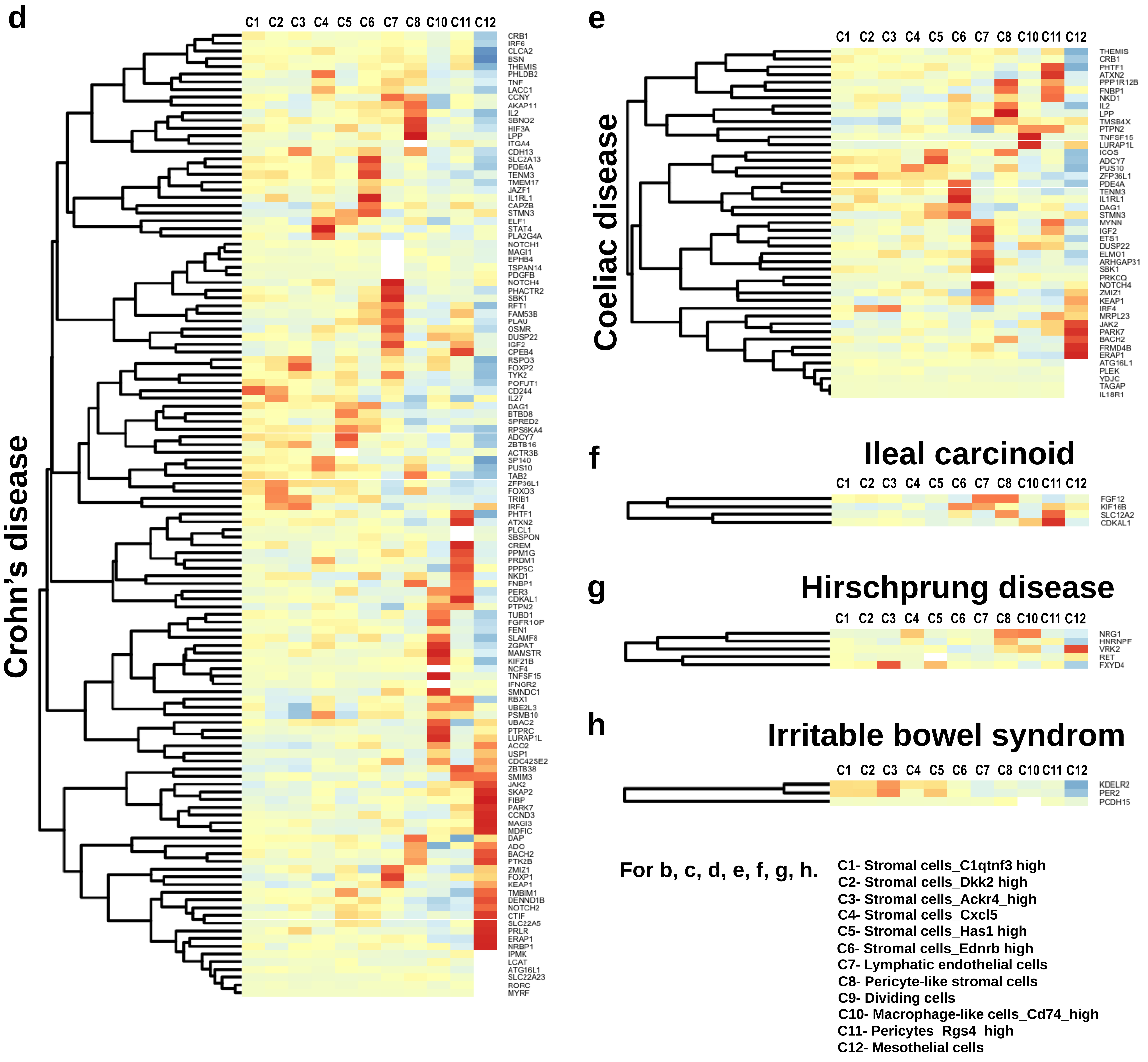

Supplementary Figure 39. Heatmap of gastrointestinal stromal cluster expression of genes correlated to GWAS data (related to Figure 7).

(a) Stomach stromal cluster expression related to stomach cancer-linked loci. (b-h) Intestinal stromal cluster expression related to colorectal cancer (b)-, inflammatory bowel disease (IBD) (c)-, Crohn's disease (d)-, Coeliac disease (e)-, ileal carcinoid (f)-, Hirschprung disease (g)- and irritable bowel syndrome (h)-linked loci. The color scheme is based on z-transformed gene expression among the clusters (the map shows genes with z > 1.65). Each row represents one gene and each column is a single cell type.

Supplementary Figure 40. related to figure 7

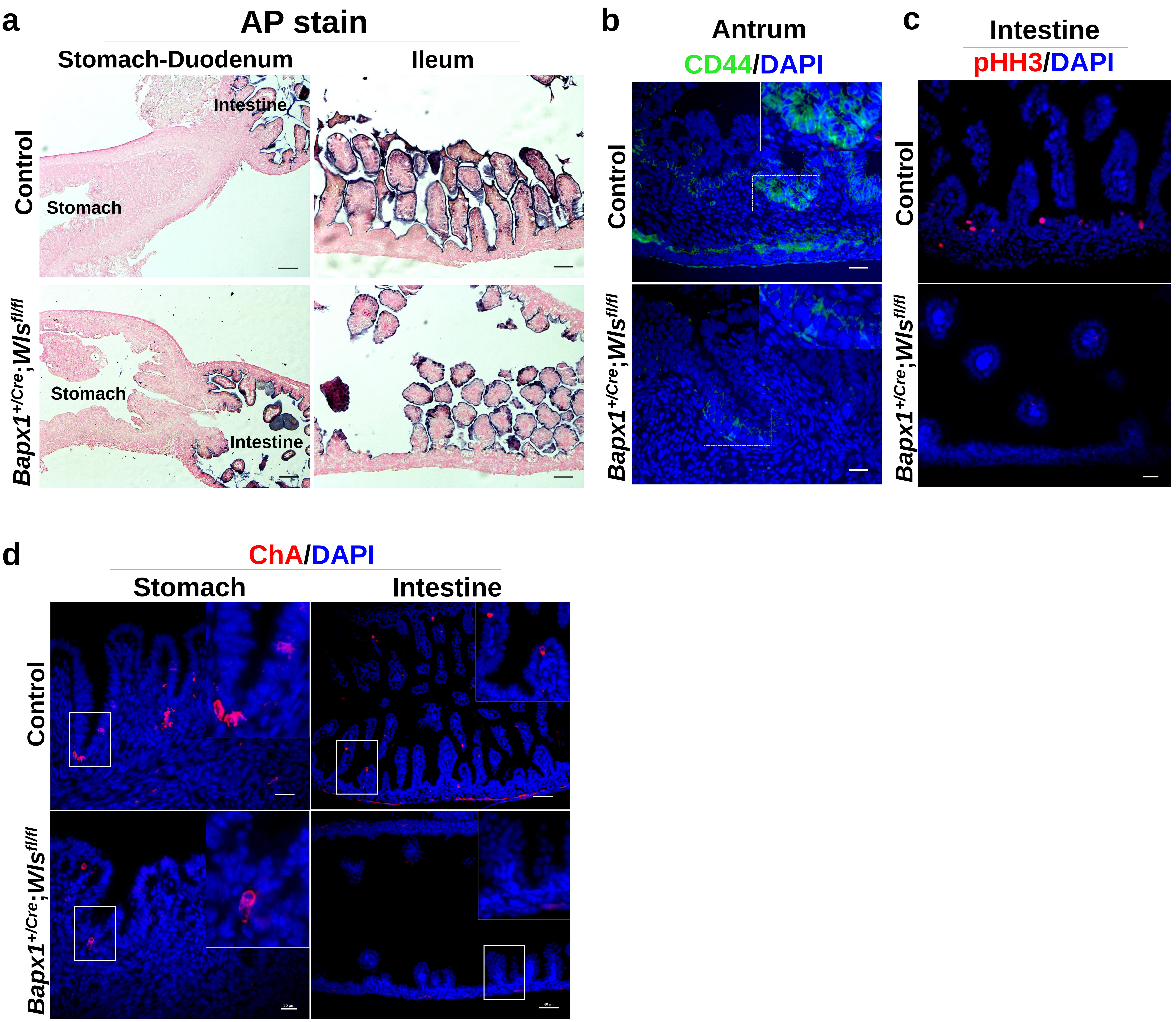

Supplementary Figure 40. Normal gastrointestinal specification and stem cell defects in *Bapx1*$^{+/Cre}$;*Wls*$^{fl/fl}$ mice (related to Figure 7).

(a) The expression of alkaline phosphatase was properly restricted to the intestine in *Bapx1*$^{Cre/+}$;*Wls*$^{fl/fl}$ mice. Scale bars indicate 200 µm (left panel) and 100 µm (right panel). (b) IF of CD44 shows its dramatically reduced expression in the *Bapx1*$^{+/Cre}$;*Wls*$^{fl/fl}$ antrum compared to the control. Scale bars indicate 25 µm. (c) IF of pHH3 (phospho-Histone H3) shows its significantly reduced expression in the *Bapx1*$^{+/Cre}$;*Wls*$^{fl/fl}$ intestine compared to controls. Scale bars indicate 25 µm. (d) The number of enteroendocrine cells stained by ChA antibody was reduced in *Bapx1*$^{+/Cre}$;*Wls*$^{fl/fl}$ stomachs and intestines compared to the controls. Scales indicate 25 µm (left panel) and 50 µm (right panel). n=3 per group.

Supplementary Figure 41. related to figure 7e and 7i

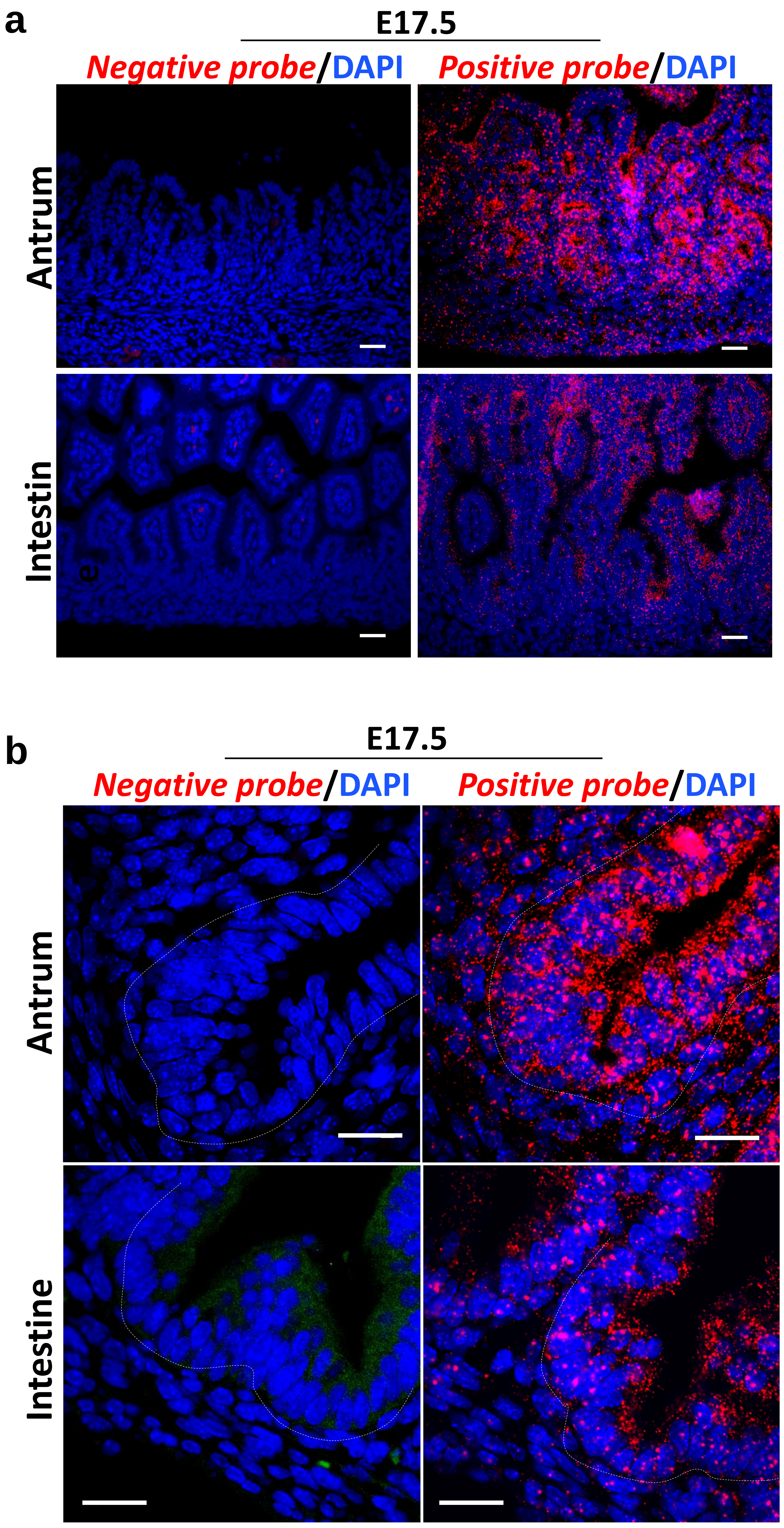

Supplementary Figure 41. smFISH images of negative and positive probes in gastrointestinal tissues (related to Figure 7e and 7i).

(a, b) Low and high magnification images of negative control (left) with a probe targeting *DapB* and of positive control (right) with a probe targeting *Ubc* (red) in the antrum (upper panel) and ileum (lower panel) in gastrointestinal tissues related to Fig. 7e and 7i. Scale bars indicate 20 μm.

Supplementary Table 1

Primer list	Sequence
Wnt2b	Fwd-CGAGGTGGCAAACATCCTAT, Rev-CTTTGAAGGCTCCACTCCTG
Wnt4	Fwd-AGACGTGCGAGAAACTCAAAG, Rev-GGAACTGGTATTGGCACTCCT
Wnt9a	Fwd-GGCCCAAGCACACTACAAG, Rev-AGAAGAGATGGCGTAGAGGAAA
Gli2	Fwd-GCTCAGCCTTTGGACACACA, Rev-GGGTAGGCATGGTGCTGATG
Foxl1	Fwd-TGCCGCATTCCACAGCATAGTC, Rev-CAAAGTGAGTTCCAGGACAGCCAG
aSMA	Fwd-GAACACGGCATCATCACCAAC, Rev-CTCCAGAGTCCAGCACAATACC

SUPPLEMENTARY REFERENCES

1. Yamashita, S. The post-embedding method for immunoelectron microscopy of mammalian tissues: a standardized procedure based on heat-induced antigen retrieval. Methods Mol Biol 657, 237-248, 19 (2010).

2. MacArthur, J. et al. The new NHGRI-EBI Catalog of published genome-wide association studies (GWAS Catalog). Nucleic Acids Res 45, D896-D901 (2017).
