# Supplementary material for: Single cell and genetic analyses reveal conserved populations and signaling mechanisms of gastrointestinal stromal niches
Source: Nat Commun. 2020 Jan 17;11:334. doi: 10.1038/s41467-019-14058-5 (PMC6969052; doi:10.1038/s41467-019-14058-5)
Supplement: Supplementary file 2 — Reporting Summary [file 41467_2019_14058_MOESM2_ESM.pdf]

nature research

Corresponding author(s): Tae-Hee Kim

Last updated by author(s): Dec 9, 2019

Reporting Summary

Nature Research wishes to improve the reproducibility of the work that we publish. This form provides structure for consistency and transparency in reporting. For further information on Nature Research policies, see Authors & Referees and the Editorial Policy Checklist .

Statistics

For all statistical analyses, confirm that the following items are present in the figure legend, table legend, main text, or Methods section.

n/a	Confirmed	
☐	☒	The exact sample size (*n*) for each experimental group/condition, given as a discrete number and unit of measurement
☐	☒	A statement on whether measurements were taken from distinct samples or whether the same sample was measured repeatedly
☐	☒	The statistical test(s) used AND whether they are one- or two-sided<br>*Only common tests should be described solely by name; describe more complex techniques in the Methods section.*
☒	☐	A description of all covariates tested
☒	☐	A description of any assumptions or corrections, such as tests of normality and adjustment for multiple comparisons
☐	☒	A full description of the statistical parameters including central tendency (e.g. means) or other basic estimates (e.g. regression coefficient) AND variation (e.g. standard deviation) or associated estimates of uncertainty (e.g. confidence intervals)
☐	☒	For null hypothesis testing, the test statistic (e.g. *F*, *t*, *r*) with confidence intervals, effect sizes, degrees of freedom and *P* value noted<br>*Give P values as exact values whenever suitable.*
☒	☐	For Bayesian analysis, information on the choice of priors and Markov chain Monte Carlo settings
☐	☒	For hierarchical and complex designs, identification of the appropriate level for tests and full reporting of outcomes
☒	☐	Estimates of effect sizes (e.g. Cohen's *d*, Pearson's *r*), indicating how they were calculated

Our web collection on statistics for biologists contains articles on many of the points above.

Software and code

Policy information about availability of computer code

Data collection	No software was used for data collection
Data analysis	Read trimming: trimmomatic v0.32<br>Genome alignment: STAR v2.5.1b (scRNA-Seq), kallisto 0.42.2 (bulk RNA-Seq)<br>Gene-level counting: featureCounts v1.5.0 (scRNA-Seq), tximport (bulk RNA-Seq)<br>Single cell analysis: Seurat v2.3.4 for clustering and visualization, Magic for recovering gene interaction, and modified RAFSIL for random forest based similarity<br>Differential expression analysis: DESeq2 v1.14.1<br>GO Enrichment: gProfiler rev 1741 build date 2017-10-19<br>Enrichment Map of GO terms: Cytoscape v3.6.0 using the EnrichmentMap app v2.0.1<br>ImageJ for image analysis

For manuscripts utilizing custom algorithms or software that are central to the research but not yet described in published literature, software must be made available to editors/reviewers. We strongly encourage code deposition in a community repository (e.g. GitHub). See the Nature Research guidelines for submitting code & software for further information.

Data

Policy information about availability of data

All manuscripts must include a data availability statement. This statement should provide the following information, where applicable:

- Accession codes, unique identifiers, or web links for publicly available datasets
- A list of figures that have associated raw data
- A description of any restrictions on data availability

Single cell RNA-seq data generated in this study is available in the GEO database repository under accession number GSE116514.
ChIP-seq data generated in this study have been deposited in GEO under the accession GSE114449 (stomach) and GSE103690 (intestine).

RNA-seq data generated in this study have been deposited in GEO under the accession GSE114450 (stomach) and GSE103683 (intestine).
All other relevant data supporting the key findings of this study are available within the article, in the supplementary files, or from the corresponding author upon reasonable request.

Field-specific reporting

Please select the one below that is the best fit for your research. If you are not sure, read the appropriate sections before making your selection.

[x] Life sciences [ ] Behavioural & social sciences [ ] Ecological, evolutionary & environmental sciences

For a reference copy of the document with all sections, see nature.com/documents/nr-reporting-summary-flat.pdf

Life sciences study design

All studies must disclose on these points even when the disclosure is negative.

Sample size	Sample size was dependent on litter sizes produced from genetic crosses. A minimum sample number of 3 was used for all analyses of genetic mouse models.
Data exclusions	No data was excluded
Replication	Bulk mRNA-seq for E17.5 stomach, intestine, and mutants: 2 replicates each. Single-cell RNA-seq for adult stomach and intestine: 1 replicate after pulling 3 mice each.
Randomization	This study does not contain groups that require randomization.
Blinding	Samples were analyzed in random order and the investigators were blinded during quantification. However, due to the dramatic phenotypes observed in our mutants, complete blinding was not always possible.

Reporting for specific materials, systems and methods

We require information from authors about some types of materials, experimental systems and methods used in many studies. Here, indicate whether each material, system or method listed is relevant to your study. If you are not sure if a list item applies to your research, read the appropriate section before selecting a response.

Materials & experimental systems

n/a	Involved in the study	
[ ]	[x]	Antibodies
[x]	[ ]	Eukaryotic cell lines
[x]	[ ]	Palaeontology
[ ]	[x]	Animals and other organisms
[x]	[ ]	Human research participants
[x]	[ ]	Clinical data

Methods

n/a	Involved in the study	
[ ]	[x]	ChIP-seq
[ ]	[x]	Flow cytometry
[x]	[ ]	MRI-based neuroimaging

Antibodies

Antibodies used	For IF: anti-Cdx2 (MU392A-UC, Biogenex, 1:300), anti-BrdU (BDB347580, Fisher scientific, 1:200), anti-CD44 (550538, BD pharmigen, 1:100), anti-Sox9 (AB5535MI, EMD Millipore, 1:200), anti-p63 (619001, BioLegend, 1:300), anti-H,K-ATPase (D032-3H, MBL, 1:400), anti-pHH3 (05-806, Millipore, 1:200), anti-PCNA (18-0110, Invitrogen, 1:200), anti-αSMA (ab124964, Abcam, 1:2000), anti-Desmin (Ab32362, Abcam, 1:1000), anti-PDGFRβ (Ab32570, Abcam, 1:100), anti-PDGFRα (SC-338, Santa Cruz, 1:200), anti-Nestin (Ab6142, Abcam, 1:200), anti-s100β (ab41548, Abcam, 1:200), anti-GFAP (Z0334, Dako, 1:300), anti-Lyve1 (11-034, Angiobio, 1:400), anti-CD34 (ab81289, abcam, 1:300), anti-CD31 (550274, BD Bioscience, 1:300) and E-Cadherin (610183, BD Bioscience, 1:300)<br>For CyTOF: Ly6c-115In (128002, HK1.4, Biolegend, 1:300), CD44-141Pr (103002, IM7, Biolegend, 1:800), Anti-Lyve-1-PE (D225-5, ALY7, MBL international, 1:300), Anti-PE-45Nd (408202, PE001, Biolegend, 1:100), αSMA-146Nd (14-9760-82, 1A4, Invitrogen, 1:500), CD24-150Nd (101829, M1/69, Biolegend, 1:800), PDGFRβ-151Eu (136002, APB5, Biolegend, 1:50), PDGFRα-156Gd (14-1401-82, APA5, Biolegend, 1:300), Sca1-164Dy (3164005B, D7, Fluidigm, 1:200), CD31-165Ho (102425, 390, Biolegend, 1:800), CD9-166Er (NBP1-44876, EM-04, Novusbio, 1:300), CD177-169Tm (553352, 2B8, BD Biosciences, 1:300), CD34-173Yb (119302, MEC14.7, Biolegend, 1:100), Anti-PDPN-FITC (127415, 8.1.1, BioLegend, 1:300), Anti-FITC-174Yb (3174006B, FIT22, Fluidigm, 1:200)<br>Secondary antibodies: Alexa Fluor 594-, 488- conjugated anti-rabbit, rat or mouse IgG (Invitrogen)
Validation	All antibodies for IF were validated on control stomach or intestinal tissues prior to use for experiment.<br>All antibodies used for CyTOF were validated with positive and negative cell line or tissues for each antibody to find proper concentration.

For mouse anti-PDX1 (DSHB, F109-D12), mouse anti-EpCam [G8.8] (DSHB, G8.8) from DSHB, see company website for originating publication of the antibody.

Animals and other organisms

Policy information about studies involving animals; ARRIVE guidelines recommended for reporting animal research

Laboratory animals

Bapx1Cre (a gift from Dr. Warren Zimmer), Sufuf/f (a gift from Dr. Chi-chung Hui), Spopf/f (obtained from the KOMP Repository), Ng2-Cre (Stock number: 029926), Wlsf/f (Stock number: 012888), ROSA26-tdTomato (Stock number: 007914) and ROSA26-SmoM2 (Stock number: 005130), and Rosa26-mTmG (Stock number: 007676) mice were purchased from The Jackson Laboratory.

Wild animals

Our study did not involve wild animals.

Field-collected samples

Our study did not involve samples collected from the field.

Ethics oversight

All procedures involving animals were performed in compliance with the Animals for Research Act of Ontario and the Guidelines of the Canadian Council on Animal Care. The Toronto Centre for Phenogenomics (TCP) Animal Care Committee reviewed and approved all procedures conducted on animals at TCP

Note that full information on the approval of the study protocol must also be provided in the manuscript.

ChIP-seq

Data deposition

☒ Confirm that both raw and final processed data have been deposited in a public database such as GEO.

☒ Confirm that you have deposited or provided access to graph files (e.g. BED files) for the called peaks.

Data access links
May remain private before publication.

https://www.ncbi.nlm.nih.gov/geo/query/acc.cgi?acc=GSE103690
https://www.ncbi.nlm.nih.gov/geo/query/acc.cgi?acc=GSE114449

Files in database submission

1A_WCE_st1_R1.fastq.gz
1A_WCE_st1_R2.fastq.gz
2A_WCE_st2_R1.fastq.gz
2A_WCE_st2_R2.fastq.gz
6A_gli2_st2_R1.fastq.gz
6A_gli2_st2_R2.fastq.gz
11A_k27_st1_R1.fastq.gz
11A_k27_st1_R2.fastq.gz
12A_k27_st2_R1.fastq.gz
12A_k27_st2_R2.fastq.gz
15A_k36_st1_R1.fastq.gz
15A_k36_st1_R2.fastq.gz
16A_k36_st2_R1.fastq.gz
16A_k36_st2_R2.fastq.gz
Gli2_St2_peaks.narrowPeak
K27_St1_peaks.broadPeak
K27_St2_peaks.broadPeak
K36_St1_peaks.broadPeak
K36_St2_peaks.broadPeak

7A_gli2_int1_R1.fastq.gz
7A_gli2_int1_R2.fastq.gz
8A_gli2_int2_R1.fastq.gz
8A_gli2_int2_R2.fastq.gz
13A_k27_int1_R1.fastq.gz
13A_k27_int1_R2.fastq.gz
14A_k27_int2_R1.fastq.gz
14A_k27_int2_R2.fastq.gz
18A_k36_int1_R1.fastq.gz
18A_k36_int1_R2.fastq.gz
19A_k36_int2_R1.fastq.gz
19A_k36_int2_R2.fastq.gz
3A_WCE_int1_R1.fastq.gz
3A_WCE_int1_R2.fastq.gz
4A_WCE_int2_R1.fastq.gz
4A_WCE_int2_R2.fastq.gz

7A_gli2_int1_filtered_intersect.bigwig
8A_gli2_int2_filtered_intersect.bigwig
13A_k27_int1_filtered_intersect.bigwig
14A_k27_int2_filtered_intersect.bigwig
18A_k36_int1_filtered_intersect.bigwig
19A_k36_int2_filtered_intersect.bigwig
3A_WCE_int1_filtered_intersect.bigwig
4A_WCE_int2_filtered_intersect.bigwig
K27_Int_merged_nodup.BED
K36_Int_merged_nodup.BED
Gli2_int_merged_nodup.BED

Genome browser session
(e.g. UCSC)

https://genome.ucsc.edu/cgi-bin/hgTracks?db=mm9&lastVirtModeType=default&lastVirtModeExtraState=&virtModeType=default&virtMode=0&nonVirtPosition=&position=chr13%3A63637092%2D63691080&hgsid=741891861_vagao1ecZrCQOBufKvngSgww8EXo

Methodology

Replicates

Intestine and stomach from two E17.5 Sufu;Spop DKO embryos were used for each experiment of the two experimental replicates. The samples were lysed and split into GLI2, H3K27ac and H3K36me3 ChIP-Seq experiments. For intestine, the replicates had similar numbers of reads and mapped reads. GLI2 ChIP had 400 overlapping peaks between the two replicates (each with ~1000 peaks called). Comparison of ChIP-Seq signals at the GLI2 peak regions merged from the two replicates revealed 94% Pearson correlation score between the two replicates (this was calcuated by multiBigWigsummary tool from DeepTools). H3K27ac ChIP had 20774 overlapping peaks between the two replicates (each with ~25000 and ~32000 peaks called). H3K36me3 ChIP had 57634 overlapping peaks between the two replicates (each with ~80000 peaks called). For stomach, one of the Gli2 ChIP experiments produced very weak signal (less than 10 peaks called), and was not included in downstream analysis. The remaining sample had 6376 out of 7378 peaks (q<0.05) with fold enrichment >5. H3K27ac ChIP had 25452 overlapping peaks between the two replicates (each with ~37000 and ~38000 peaks called). H3K36me3 ChIP had 55617 overlapping peaks between the two replicates (each with ~107000 peaks called).

Sequencing depth

Adaptor-ligated DNAs were amplified with index primer, universal primer, and NEBNext Q5 Hot Start HiFi PCR Master Mix. After incubation at 98C for 30s, DNAs were subjected to 16 repeated cycles of incubation—98C for 10s and 65C for 75s—followed by extension at 65C for 5 minutes.
All reads were 126bp, paired-end.
Each sample had 14 million to 22 million total reads and ~90% high quality reads (uniquely mapped, mapping quality >5).

Antibodies

GLI2 antibody was generated by our laboratory. Validation: This homemade antibody has been used extensively in the lab for immunoprecipitation and western blot, demonstrating high specificity (PMID 231668383, PMID 25581370, PMID 23034632 and PMID 21795282). For ChIP-Seq, we tested this antibody by performing ChIP followed by qPCR analysis for known GLI2 binding sites in the promoters of Ptch1 and Gli1, in comparison with qPCR analysis for the regions without Gli binding consensus. After confirmation of significant enrichment, we proceed with ChIP-Seq. GLI2 ChIP-Seq libraries also were subjected to qPCR validation for the known GLI binding regions.
H3K27ac antibody: Millipore 05-1334, lot 2489078. This antibody has been used in numerous published data sets, including GSE64055, GSE67875, and GSE97837. The library was subjected to qPCR analysis for the promoter of Actb, where enrichment of H3K27ac is expected; this result was compared to qPCR analysis for gene desert regions.
H3K36me3 antibody: Abcam ab9050, lot GR204353-1. This antibody is provided by a validation statement, which appears on the manufacture's website, where hundreds of ChIP-Seq related citations can be found. The library was subjected to qPCR analysis for the first exon of Actb, where enrichment of H3K36me3 is expected; this result was compared to qPCR analysis for gene desert regions.

Peak calling parameters

The mm9 reference genome was downloaded from http://hgdownload.soe.ucsc.edu/goldenPath/mm9/bigZips/chromFa.tar.gz and then concatenated.
All reads were aligned by: bwa mem mm9.fa forward.fastq.gz reverse.fastq.gz > aligned.sam
The aligned reads were filtered, using bamtools to retain uniquely mapped, mapping quality >5, and properly paired reads. Then, ENCODE blacklist regions were filtered. The resulting filtered bam files were converted into BigWig format for visualization and used for peak calling.
Peak calling was performed on individual replicates with their corresponding input DNAs as a control: macs2 callpeak -t ChIP_filtered.bam -c input_filtered.bam -n name -g mm -f BAMPE --keep-dup auto -q 0.05
--broad and --broad-cutoff 0.05 were used for histone marks.
To merge peaks detected in the biological replicates, the narrowPeak or broadPeak files were concatenated, sorted, then merged, using "mergeBed" from BedTools.

Data quality

Intestine
GLI2: 1686 out of 1833 peaks (q<0.05) had fold enrichment >5.
H3K27ac: 14885 out of 38566 peaks (q<0.05) had fold enrichment >5.
H3K36me3: 1590 out of 133318 peaks (q<0.05) had fold enrichment >5.

Stomach:
GLI2: 6376 out of 7378 peaks (q<0.05) had fold enrichment >5.
H3K27ac: 19318 out of 37943 peaks (q<0.05) had fold enrichment >5.
H3K36me3: 15909 out of 94985 peaks (q<0.05) had fold enrichment >5.

Software

Adaptor sequences were trimmed from reads, using Trimmomatic 0.32, and aligned to the mouse mm9 reference genome, using BWA-MEM10 with default parameters. Bamtools and BEDTools were used to filter reads. MACS2 was used to call peaks. Genes were mapped with GREAT. Super enhancers were called, using ROSE.

Flow Cytometry

Plots

Confirm that:

- [x] The axis labels state the marker and fluorochrome used (e.g. CD4-FITC).
- [x] The axis scales are clearly visible. Include numbers along axes only for bottom left plot of group (a 'group' is an analysis of identical markers).
- [x] All plots are contour plots with outliers or pseudocolor plots.
- [x] A numerical value for number of cells or percentage (with statistics) is provided.

Methodology

Sample preparation

Murine adult gut tissue: Mesenchymal cell isolation from the stomach and intestinal tissues was modified from the previously described method53. Mice were dissected in ice-cold Wash Buffer; HBSS (Gibco) with 2% (vol/vol) of fetal calf serum (Gibco) and 10 mM HEPES (15630-080, Gibco). Intestines were cut into small pieces and moved to pre-warmed Predigestion Buffer: 30 ml of HBSS with 10% (vol/vol) inactivated FBS (080-105, Wisent Bioproducts), 10 mM HEPES and 5mM EDTA (15575-038, Invitrogen). This solution was then incubated for 20 min at 37˚C under rotation (220 rpm) in a thermal incubator in a 50 ml tube, followed by filtering through a 100 μm cell strainer. The flow-through contains stomach or intestinal contents decanted off epithelial cells. These gut pieces were incubated for 20 min at 37 ˚C under slow rotation, followed by filtering through a 70 μm cell strainer. After wash off the remaining EDTA with Wash Buffer, the tissues were collected into 50 ml tubes containing 20 ml of Digestion Buffer; 1% (vol/vol) penicillin-streptomycin antibiotics (P4333, Sigma), 10% inactivated FBS, 15 mM HEPES, 100U/ml of DNaseI (LS002139, Worthington), 25U/ml of collagenase IV (LS004186, Worthington), 0.3g/100ml of Dispase (04942078001, Roche). The tissues were digested by incubation at 37 ˚C for 30 min under slow rotation. After vortexing the cell solution intensely for 20 s every 10 minutes, the tissues were passed through a 40 μm cell strainer. After collecting them into 50 ml tubes with 20 ml fresh Digestion Buffer, the previous incubation process was repeated. After combining the supernatants from digestion steps and centrifugation for 5 min at 400g at 20 ˚C, the pellets were resuspended in Wash Buffer for FACS. CytoxBlue (S34857, Invitrogen) staining was used to distinguish dead cells. Tdtomato positive cells were sorted using MoFlo Astrios (Beckman Coulter).

Instrument

Beckman Coulter Mo Flo XDP and MoFlo Astrios sorter

Software

FlowJo (version 9.8.3)

Cell population abundance

tdTomato+ mesenchymal cells in adult: Bapx1Cre: 30-40% of live cells, Ng2-Cre: 5% of live cells

Gating strategy

Debris excluded by FSC-A vs. BSC-A. Doublets excluded by FSC-W vs. FSC-H. Live cells were gated using SYTOX Blue Dead Cell Stain. tdTomato+/- was determined using tdTomato negative littermate controls from the same batch of samples.

- [x] Tick this box to confirm that a figure exemplifying the gating strategy is provided in the Supplementary Information.
