# Supplementary material for: Single cell and genetic analyses reveal conserved populations and signaling mechanisms of gastrointestinal stromal niches
Source: Nat Commun. 2020 Jan 17;11:334. doi: 10.1038/s41467-019-14058-5 (PMC6969052; doi:10.1038/s41467-019-14058-5)
Supplement: Supplementary file 3 — Description of Additional Supplementary Files [file 41467_2019_14058_MOESM3_ESM.pdf]

**Description of Additional Supplementary Files**

**Supplementary Data 1.** List of differentially expressed marker genes for each cluster in the stomach and the intestine reported in Figure 1a -d.
